# Soluble CD95L triggers Caspase-10-driven reactive oxygen species production in neutrophils and aggravates anti-neutrophil cytoplasmic antibody-vasculitis

**DOI:** 10.1038/s41467-026-74452-8

**Published:** 2026-06-20

**Authors:** Andréa Boizard-Moracchini, Dhouha Msalbi, Sarah Huot-Marchand, Layla Haymour, Eden Lebrault, John Tchen, Titia Jekel, Gaël Galli, Benoît Brilland, Nathalie Mérillon, Coralie Josensi, Clément Lozano, Juliana Mazars, Matthieu Douard, Yannick Danger, Franck Vérité, Thomas Barnetche, Estibaliz Lazaro, Pierre Duffau, Pierre Pfirmann, Aude Magerus-Chatinet, Mickael Jean, Jean-François Augusto, Jean Armengaud, Christophe Richez, Pierre Vacher, Patrick Legembre, Patrick Blanco

**Affiliations:** 1 University of Bordeaux, CNRS, ImmunoConcEpT, UMR, Bordeaux, France; 2 Department of Immunology and Immunogenetics, Bordeaux University Hospital, Bordeaux, France; 3 INSERM U1262, UMR 7276, CRIBL, University of Limoges, Limoges, France; 4 Centre national de référence maladie auto-immune et systémique rares Est/Sud-Ouest (RESO), Bordeaux University Hospital, Bordeaux, France; 5 Service de Néphrologie-Dialyse-Transplantation, University of Angers, CHU Angers, Angers, France; 6 Univ Angers, Nantes Université, Inserm, CNRS, SFR ICAT, CRCI2NA, Angers, France; 7 University of Paris-Saclay, CEA, INRAE, Département Médicaments et Technologies pour la Santé (DMTS), SPI, Bagnols-sur-Cèze, France; 8 University of Bordeaux, INSERM U1045, Centre de Recherche Cardio-Thoracique de Bordeaux, Pessac, France; 9 EFS Rennes, Rue Pierre Jean Gineste, Rennes, Cedex France; 10 Internal Medicine Department, Haut-Leveque, Bordeaux University Hospital, Pessac, France; 11 Internal Medicine Department, Saint André, Bordeaux University Hospital, Bordeaux, France; 12 Nephrology Department, Bordeaux University Hospital, Bordeaux, France; 13 INSERM U1163. IMAGINE - Institut des Maladies Génétiques, Paris, France; 14 University of Rennes, CNRS, ISCR – UMR 6226, Rennes, France; 15 Department of Rheumatology, Pellegrin, Bordeaux University Hospital, Bordeaux, France; 16 Present Address: CIRI-Centre International de Recherche en Infectiologie, Team GIMAP, Inserm, U1111, CNRS, UMR5308, Université Saint-Etienne, Bordeaux, France

**Keywords:** Autoimmunity, Ion channel signalling, Innate immune cells, Inflammation

## Abstract

Anti-neutrophil cytoplasmic antibody (ANCA)-associated vasculitis (AAV) is a severe autoimmune disease that lacks effective targeted therapies. T cell and neutrophil activation are associated with tissue lesions in ANCA-associated vasculitis responsible for the necrotizing vasculitis of small blood vessels. Although the aberrant release of reactive oxygen species (ROS) by neutrophils contribute to the disruption of the endothelial barrier, the underlying molecular mechanisms of this oxidative burst remain unclear. Here, we observe that blood vessels in the inflamed organs of patients with AAV express CD95L, which is cleaved by metalloproteases to release soluble CD95L (sCD95L). sCD95L stimulates ROS production in AAV neutrophils via a caspase-driven mechanism. Proteomic analysis reveals that the deubiquitinase OTULIN is a caspase substrate in sCD95L-exposed neutrophils. Caspase-10 cleaves OTULIN after its aspartates at positions 31 and 54 to unleash the activity of E3 ligase complex LUBAC and trigger mitochondrion-dependent ROS production in AAV neutrophils. Inhibition of the CD95-mediated non-apoptotic signaling abrogates ROS production in AAV neutrophils and alleviates clinical symptoms in AAV and crescentic glomerulonephritis mouse models, indicating that CD95 and CD95L represent attractive molecular targets for patients with AAV.

## Abstract (web summary)

Excessive Reactive oxygen species (ROS) production by neutrophils drives necrotizing inflammation of small blood vessels in Anti-neutrophil cytoplasmic antibody (ANCA)–associated vasculitis (AAV). However, the mechanism responsible for the dysregulated ROS production remains uncharacterized. Here, the authors report the contribution of soluble CD95L to neutrophil activation and ROS production and show that sCD95L—exposed AAV neutrophils undergo Caspase-10 cleavage of OTULIN, LUBAC activation and downstream mitochondrion-dependent ROS production and propose targeting of CD95 signaling as a promising therapeutic strategy in AAV.

## Introduction

Anti-neutrophil cytoplasmic antibody (ANCA)–associated vasculitis (AAV) comprises a group of autoimmune diseases characterized by necrotizing inflammation of small blood vessels. AAV is defined by the presence of autoantibodies directed against myeloperoxidase (MPO-ANCA) or proteinase 3 (PR3-ANCA)^1^. Although multiple organs may be involved, the kidneys and lungs are most commonly affected in patients with AAV^2^. ANCA-associated glomerulonephritis (GN) is the leading cause of rapidly progressive GN^3^ and frequently progresses to end-stage renal disease^4^. Diffuse alveolar hemorrhage represents one of the most severe complications of AAV, often leading to respiratory failure that requires intensive care and invasive mechanical ventilation^5^. Growing evidence implicates neutrophil dysregulation as a key driver of small-vessel injury in AAV^6^.

In particular, excessive production of reactive oxygen species (ROS) by neutrophils contributes to vascular damage^7,8^ through mechanisms that remain incompletely understood. A deeper understanding of these pathogenic pathways is therefore essential to improve disease monitoring and to develop novel therapeutic strategies.

CD95L (FasL/CD178) is a member of the tumor necrosis factor (TNF) family, and when expressed on the surface of activated T lymphocytes and natural killer (NK) cells, it regulates the immune response^9^. Early studies attempted to induce immune tolerance via CD95L expression in grafted tissues; however, this approach unexpectedly triggered a neutrophil-driven inflammatory response that promoted graft rejection^10–13^. These findings suggest a functional interplay between CD95L and aberrant neutrophil chemotaxis, although the underlying molecular mechanisms remain poorly understood.

Most studies on CD95L and its receptor CD95 (also known as Fas) have focused on deciphering how transmembrane CD95L (mCD95L) induces apoptosis^9^. However, mCD95L can be cleaved by metalloproteases (MMPs) to release soluble-CD95L (sCD95L)^14^, which does not trigger cell death but instead activates pro-inflammatory signaling pathways^15^. CD95 itself lacks enzymatic activity^16,17^; to implement cell signaling, it dynamically recruits different enzymes, including proteases, kinases, and phosphatases in a dynamic fashion through protein–protein interactions (PPIs)^14^. Binding of mCD95L to CD95 recruits Fas-associated death domain (FADD) at the level of the CD95 death domain (DD), which in turn aggregates caspase-8 to form the “death-inducing signaling complex” (DISC)^18^ and induces apoptosis. Caspase-8 contributes to caspase-10 recruitment, which modulates both apoptotic and non-apoptotic signals^19^ via mechanisms that remain to be elucidated. Because rodents lack the caspase 10 ortholog^20^, its pathophysiological role remains elusive. The interaction of sCD95L with CD95 does not trigger the DISC formation, but activates non-apoptotic signaling pathways, including a calcium (Ca^2+^) response that contributes to oncogenesis^21,22^ and inflammation^23–26^. The Ca²⁺ response is initiated through the intracellular domain of CD95, known as the calcium-inducing domain (CID), which interacts with the SH3 domain of phospholipase Cγ1 (PLCγ1)^27^. Based on CID identification, we synthesized a peptidomimetic mimicking the CID sequence, termed DB550^27^. DB550 crosses the plasma membrane and binds to PLCγ1, preventing its recruitment to CD95 and specifically inhibiting non-apoptotic signaling in sCD95L-exposed cells^27^.

Deubiquitinase (DUB) OTULIN modulates the activity of the linear ubiquitin assembly complex (LUBAC) in a cell-type-specific fashion, either positively or negatively^28^. The LUBAC complex comprises three proteins: Heme-oxidized IRP2 ubiquitin ligase 1 (HOIL-1L), HOIL-1-interacting protein (HOIP), and Shank-associated RH domain-interacting protein (SHARPIN) performing linear ubiquitination^29^, which promotes different inflammatory signaling pathways, including the inflammasome^30^, Toll-like receptor^31,32^ and TNF-R1^29^. Although LUBAC is a key player in the TNF-R1 signaling pathway^28,33^, its role in CD95 signaling is elusive. Nevertheless, its activity appears to be modulated by CD95 because the conditional elimination of HOIL-1 or HOIP in keratinocytes causes lethal inflammatory skin disease that can only be prevented by simultaneous elimination of TNF-R1, TRAIL-R, and CD95 in mice^33^. However, the mechanism through which CD95 activates the OTULIN/LUBAC axis and the pathophysiological consequences of this activation remain unclear.

Here, we identify an unexpected function of sCD95L in promoting neutrophil activation in anti-neutrophil cytoplasmic antibody-associated vasculitis. We show that sCD95L induces caspase-10-dependent cleavage of OTULIN, unleashing LUBAC activity and driving aberrant reactive oxygen species production in neutrophils. We further demonstrate that inhibition of CD95-mediated non-apoptotic signaling suppresses this pathway and alleviates clinical symptoms in AAV animal models. These findings establish a mechanistic link between CD95 signaling and neutrophil-driven oxidative stress in vasculitis. In addition, this study highlights CD95 and CD95L as potential therapeutic targets in inflammatory vascular diseases.

## Results

### CD95L is upregulated in patients with AAV

**Fig. 1:**
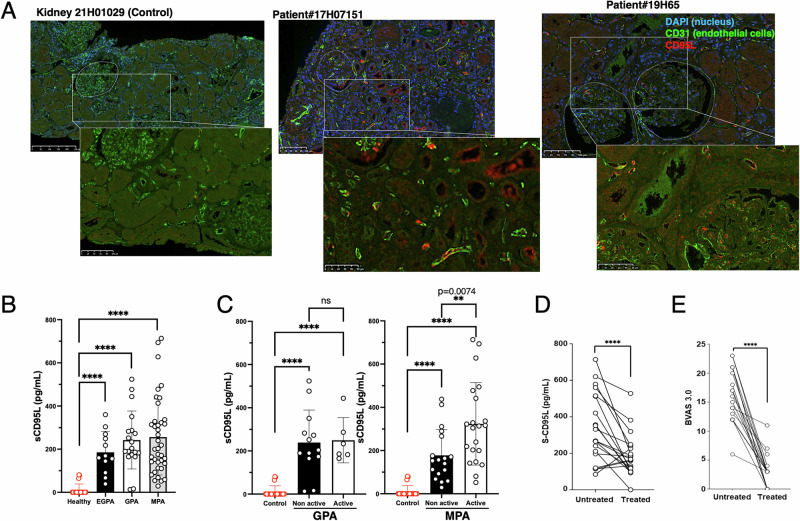
CD95L expression is increased in AAV Patients. **A** CD95L *(in red)* and CD31 (marker of endothelial cells) *(in green)* staining were performed on kidney biopsies from one healthy control (21H01029, ***left panel***) or two AAV patients (17H07151 and 19H65, ***right panel***) by immunofluorescence. Dashed thin white lines indicate glomeruli. Nuclei are stained using DAPI in blue. Scale bars represent 250 and 100 µm in the magnified images for the control kidney, 100 and 50 µm in the magnified images for Patient#17H07151 and 100 and 50 µm in the magnified images for Patient#19H65 kidney. **B**,** C** Dosage of serum sCD95L was performed by ELISA in indicated patients (EGPA: Eosinophilic Granulomatosis with Polyangiitis; GPA: Granulomatosis with Polyangiitis; MPA: Microscopic Polyangiitis). Healthy donors, *n* = 20; EGPA, *n* = 12; GPA, *n* = 19; MPA patients, *n* = 39. Active GPA, *n* = 6; non active GPA, *n*= 13. Active MPA, *n* = 21; non active MPA, *n* = 18. Data represent mean ± SD; ** *p*<0.01, **** *p* < 0.0001, using two-tailed Mann-Whitney test. **D** Comparison of serum CD95L concentrations between AAV patients before (M0) and after treatment (M6). *N* = 20. *** *p* < 0.001, **** *p* < 0.0001 using two-sided Wilcoxon test. **E**. Comparison of BVAS 3.0 between AAV patients before and after treatment of AAV patients. *N* = 20. **** *p* < 0.0001 using two-sided Wilcoxon test.

To evaluate whether the CD95/CD95L pair exerts a biological function in patients with AAV, we analyzed CD95L expression in the tissues of patients using multiplex immunofluorescence. Kidney biopsies from patients with ANCA-associated GN revealed that CD31^+^ endothelial cells expressed CD95L (Fig. 1A and Supplementary Fig. S1A), whereas no CD95L was detected in control kidneys (Fig. 1A and Supplementary Fig. S1A). Endothelial cells in skin biopsies from patients with AAV (i.e., patients with vascular purpura) also expressed CD95L (Supplementary Figure 1B), whereas no such staining was detected in a control skin (Supplementary Figure 1B).

We next investigated whether this transmembrane CD95L was cleaved by metalloproteases and accumulated in the bloodstream of patients with AAV compared to that of healthy donors (HD). AAV is a clinically heterogeneous group, which comprises patients affected by microscopic polyangiitis (MPA), granulomatosis with polyangiitis (GPA) and eosinophilic granulomatosis with polyangiitis (EGPA)^34^. By assessing the serum sCD95L concentration in patients in each group (Supplementary Table 1), we observed that sCD95L was significantly increased in the sera of EGPA (185.7 pg/mL), GPA (242.7 pg/mL), and MPA (256.4 pg/mL; Fig. 1B and Supplementary Table 1) compared to healthy participants (11.2 pg/mL; Fig. 1B). Notably, the serum CD95L levels were higher in patients with active MPA (323.5 pg/mL) than in those with inactive MPA (178.1 pg/mL; Fig. 1C). Next, we measured sCD95L concentrations in a second longitudinal cohort of patients with MPA (Supplementary Table 2). We confirmed that serum CD95L was increased in patients with MPA and revealed that it was significantly decreased upon effective immunosuppressive treatments (300.5 pg/mL *versus* 166 pg/mL, *p* < 0.0001; Fig. 1D), paralleling the decrease in the disease activity as assessed by the Birmingham Vasculitis Activity Score (BVAS 3.0) (Fig. 1E). A correlation was observed between the serum concentrations of CD95L in MPA patients and BVAS 3.0 (Supplementary Fig. 1C; Pearson *r* = 0.31, *p *< 0.05). Analyses of the sCD95L concentration with different clinical factors did not reveal any evident confounding factors. Specifically, no correlations were found with renal function (creatinine, eGFR calculated by MDRD), proteinuria, or inflammatory status (CRP) (Supplementary Fig. 2). Collectively, these findings raise questions regarding the potential implications of sCD95L in AAV pathogenesis. Given that the concentration of sCD95L was significantly increased in patients MPA (Fig. 1B) and correlated with the severity of this pathology (Fig. 1C–E), the remainder of the study focused on cells and sera isolated from MPA patients.

### sCD95L stimulates AAV neutrophil-mediated ROS production

**Fig. 2:**
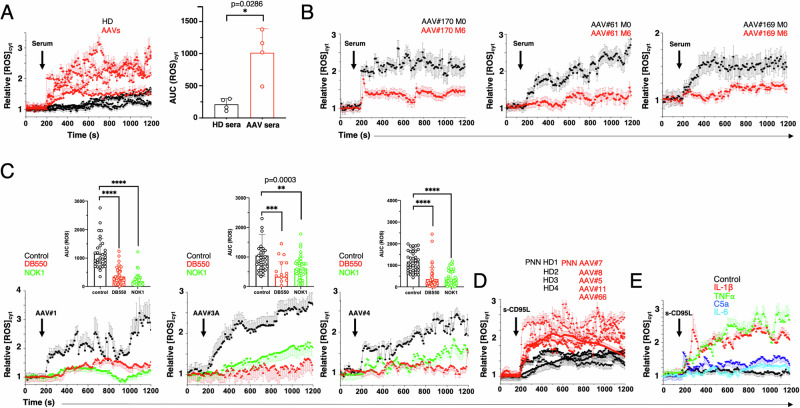
AAV neutrophils are primed to respond to sCD95L. Cytosolic ROS production ([ROS]_cyt_) was measured with the fluorescent probe H2DCFDA, Ratio values (*R*) were normalized to pre-stimulated values (*R*_0_) to yield *R*/*R*_0_ values (relative [ROS]). Sera from healthy donors and AAV patients were diluted (1/1000) in the extracellular recording medium (HBSS). Data are representative of three independently performed experiments. **A** left panel: Relative cytosolic ROS production in PNN (polynuclear neutrophils) from HDs after stimulation with sera from AAV patients (*N* = 4, red) or healthy donors (*N* = 4, black). For each condition, data represent mean ± SD of *n* = 41 cells. Right panel: Representation of areas under the curve (AUC) of the ROS production. Data represent mean ± SD; * *p* < 0.05 using two-tailed Mann-Whitney test. **B** Relative cytosolic ROS in PNN from HD after stimulation with sera from AAV patients before (M0) or after treatment (M6). For each condition, data represent mean ± SD of *n* = 41 cells. Data are representative of three independently performed experiments. **C** HD PNN were pre-treated with or without DB550 (1 µM) and then bathed with sera from AAV patients. To block CD95L/CD95 interaction, the neutralizing anti-CD95L mAb NOK-1 or isotypic control (1 µg/ml) was pre-incubated in sera for 10 min prior to incubation with HD neutrophils and relative cytosolic ROS production was measured. *Inset:* histograms represent mean ± SD of areas under the curve (AUC) of the cytosolic ROS response. ** *p* < 0.01, *** p < 0.001, **** *p* < 0.0001 using unpaired and two-tailed Mann-Whitney test. **D** Cytosolic ROS in PNN isolated from HD or AAV patients exposed to sCD95L (100 ng/ml). For each condition, data represent mean ± SD of *n* = 41 cells. Data are representative of three independently performed experiments. **E** HD neutrophils were pre-treated in the presence or absence (control) of the pro-inflammatory cytokines TNF (5 ng/ml), IL-1β (10 ng/ml), IL-6 (10 ng/ml) or C5a (10 ng/mL) for 15 min and then stimulated with sCD95L (100 ng/ml). The cytosolic ROS was assessed. For each condition, data represent mean ± SD of *n* = 41 cells. Data are representative of three independently performed experiments.

In patients with AAV, neutrophils release high levels of ROS involved in necrotizing vasculitis^7^. Consistently, neutrophils isolated from healthy donors and exposed to AAV sera underwent a stronger cytosolic ROS production (Fig. 2A) than that observed in the same cells bathed in sera from healthy donors (Fig. 2A). In addition, ROS production was associated with disease activity as this signal was reduced when neutrophils were exposed to sera isolated from the same patients after effective immunosuppressive treatment (Fig. 2B). The neutralizing anti-CD95L antibody NOK-1 inhibited this signal (Fig. 2C), indicating that sCD95L in patients with AAV contributes to the oxidative burst of neutrophils. In addition, DB550, a selective inhibitor of the CD95-mediated calcium signaling pathway^25,27^, abrogated cytosolic ROS production in HD neutrophils exposed to AAV sera (Fig. 2C), suggesting that the CD95/PLCγ1 interaction is involved in ROS signaling.

On the other hand, neutrophils isolated from patients with AAV and exposed to sCD95L underwent a stronger ROS production than that observed in neutrophils from HD (Fig. 2D), suggesting that AAV neutrophils were primed to respond to sCD95L. This process was not associated with CD95 upregulation in AAV neutrophils (Supplementary Fig. 3A). Of note, the oxidative effect of sCD95L in AAV neutrophils was dose-dependent (Supplementary Fig. 3B), with a significant effect starting at 100 pg/mL (Supplementary Fig. 3B), a concentration mainly detected in patients with AAV (Fig. 1B) but not in healthy participants (Fig. 1B). Because the pro-inflammatory cytokines IL-6^35^, TNF^36^, and IL-1β^37^ or the product of complement factor C5 cleavage, C5a^38^ are increased in patients with AAV, we investigated whether pre-incubation of HD neutrophils with these molecules could sensitize the cells to CD95 stimulation. A short pre-treatment (15 min) of HD neutrophils with TNF or IL-1β dramatically enhanced CD95-mediated ROS production in HD neutrophils, whereas IL-6 and C5a failed to do so (Fig. 2E), indicating that the CD95-driven oxidative burst in AAV neutrophils could be associated with the priming of these cells by TNF and/or IL-1β.

### sCD95L triggers Ca^2+^-dependent ROS production

**Fig. 3:**
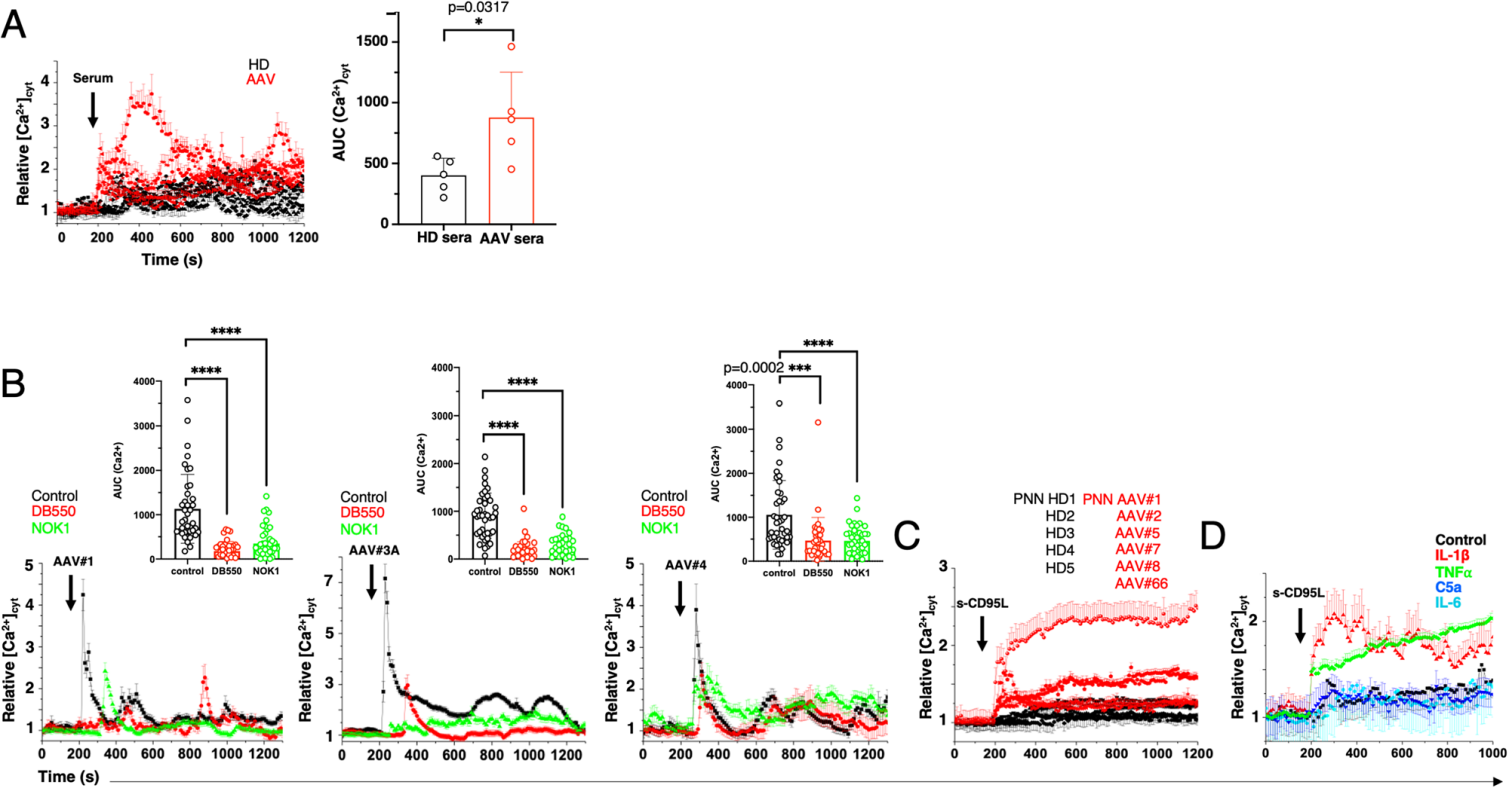
AAV neutrophils are primed to respond to sCD95L. Cytosolic calcium concentration ([Ca^2+^]_cyt_) was assessed using Cal-520-AM. Ratio values (*R*) were normalized to pre-stimulated values (*R*_0_) to yield *R*/*R*_0_ values (relative [Ca^2+^]). Data represent the mean ± SD (*n* = 41 cells). Data are representative of three independently performed experiments. Sera were injected, and calcium responses were monitored in neutrophils isolated from healthy donors and AAV patients. **A** Left panel: Relative Cytosolic Ca^2+^ concentration in neutrophils isolated from HD and exposed to sera of AAV patients or healthy donors. For each condition, data represent mean ± SD of *n* = 41 cells. Right panel: histograms of areas under the curve (AUC) of the cytosolic Ca^2+^ concentration measured in the left panel. Data represent mean ± SD; * *p* < 0,05 using two-tailed Mann-Whitney test. **B** Cytosolic Ca^2+^ concentration was assessed in HD neutrophils stimulated with sera from AAV patients. Briefly, neutrophils were pre-incubated in the presence or absence of DB550 (1 µM) for 30 min and then incubated with sera of AAV patients. To block CD95L/CD95 interaction, the neutralizing anti-CD95L mAb NOK-1 or isotypic control (1 µg/ml) was pre-incubated in the indicated sera for 10 min prior to incubation with HD neutrophils. For each condition, data represent mean ± SD of *n* = 41 cells*. Insets*: Cytosolic Ca^2+^ concentration AUC of indicated conditions. Data represent mean ± SD; *** *p* < 0.001, **** *p* < 0.0001 using unpaired and two-tailed Mann-Whitney test. **C** Neutrophils isolated from AAV patients or healthy subjects were stimulated with sCD95L (100 ng/mL) and cytosolic Ca^2+^ concentration was monitored. For each condition, data represent mean ± SD of *n* = 41 cells. Data are representative of three independently performed experiments. **D** Cytosolic Ca^2+^ concentration was assessed in PNN from HD, which were pre-incubated pre-treated in the presence or absence (control) of the pro-inflammatory cytokines TNF (5 ng/ml), IL-1β (10 ng/ml), IL-6 (10 ng/ml) or C5a (10 ng/mL) for 15 min and then stimulated with sCD95L (100 ng/ml). The cytosolic Ca^2+^ concentration was assessed. For each condition, data represent mean ± SD of *n* = 41 cells. Data are representative of three independently performed experiments.

Because DB550 inhibits CD95-mediated Ca^2+^ signal^27^, we next analyzed the Ca^2+^ response in neutrophils from patients with AAV. In HD neutrophils, the intracellular Ca^2+^ response after exposure to AAV sera was increased compared to that obtained from sera harvested from healthy subjects (Fig. 3A). In agreement with the ROS data, this Ca^2+^ signal relied on CD95 stimulation since NOK-1 and DB550 abrogated this effect (Fig. 3B). The Ca^2+^ response was amplified in AAV neutrophils exposed to sCD95L compared to neutrophils isolated from HDs (Fig. 3C). This enhanced response was phenocopied by pre-incubation of HD neutrophils with TNF or IL-1β (Fig. 3D), whereas pre-incubation with IL-6 and C5a failed to do so (Fig. 3D). Collectively, these findings indicated that AAV neutrophils undergo an increased Ca^2+^ response and strong ROS production when stimulated with sCD95L.

To identify the molecular link between sCD95L-mediated PLCγ1 activation and ROS production in the neutrophils of patients with AAV, we evaluated the effect of modulators of Ca^2+^ signaling on ROS production. Incubation of AAV neutrophils with sCD95L in Ca^2+^-free medium prevented ROS production (Supplementary Fig. 3C). Similarly, incubation of AAV neutrophils with the permeant Ca^2+^ chelator BAPTA-AM inhibited the CD95-mediated ROS production in neutrophils (Supplementary Fig. 3D), suggesting that the CD95-mediated Ca^2+^ influx was mandatory for the oxidative stress in neutrophils.

### sCD95L triggers mitochondria-dependent ROS production

Because mitochondria can produce ROS^39^, we investigated whether this organelle was responsible for CD95-mediated ROS production in AAV neutrophils. Mitotempo, a mitochondrion-targeted antioxidant, abrogated the CD95-mediated ROS production (Supplementary Fig. 3E), indicating that the AAV neutrophils exposed to sCD95L underwent a mitochondrion-driven ROS production. We previously established that the anti-apoptotic factor Bcl2, in association with the voltage-dependent anion channel (VDAC) 1, can orchestrate Ca^2+^ fluxes from the endoplasmic reticulum (ER) to the mitochondria in breast cancer cells exposed to sCD95L^40^. Therefore, we evaluated whether this Bcl2/VDAC1-driven mitochondrial Ca^2+^ uptake was involved in CD95-mediated mitochondrial ROS production in neutrophils. The Bcl2 inhibitor ABT-263 and cell-permeant L14-15 peptide (amino acids 208–218 of VDAC1), which impinges on the binding of Bcl2 to VDAC1^41^ blocked ROS production in sCD95L-exposed AAV neutrophils (Supplementary Fig. 3F). Overall, these findings indicate that sCD95L triggered Bcl2/VDAC1-driven transfer of Ca^2+^ from the ER to the mitochondria in neutrophils from patients with AAV to promote mitochondrial ROS production.

### ROS production relies on CD95-mediated caspase activation in neutrophils

**Fig. 4:**
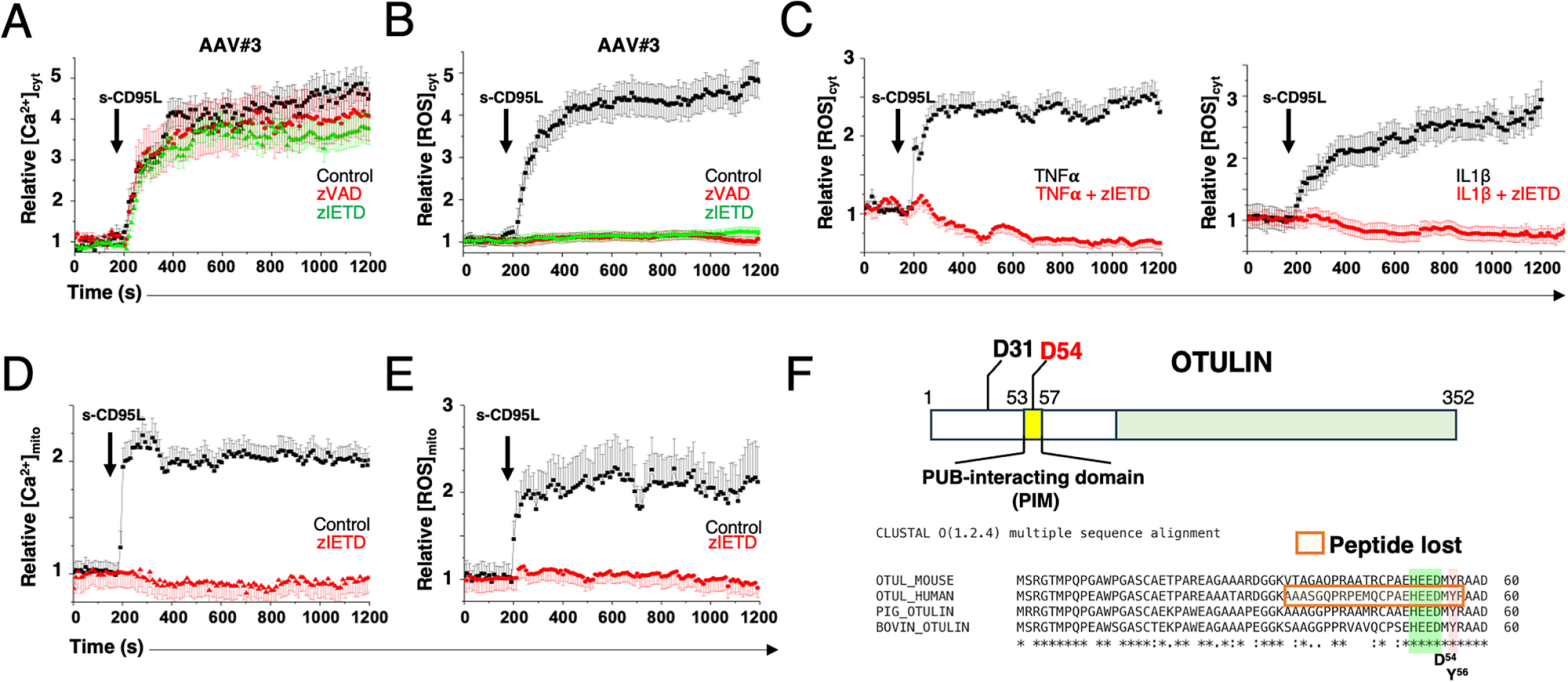
The CD95-mediated ROS production in AAV neutrophils relies on a caspase-8-driven Ca^2+^ flux from ER to mitochondria. Cytosolic ROS production ([ROS]_cyt_) was measured with the fluorescent probe H2DCFDA, the cytosolic Ca^2+^ concentration ([Ca^2+^]_cyt_) with Cal-520-AM, the mitochondrial ROS ([ROS]_mito_) and the mitochondrial calcium ([Ca^2+^]_mito_) were measured using MitoSOX and Rhod2-AM probes, respectively. Ratio values (*R*) were normalized to pre-stimulated values (*R*_0_) to yield *R*/*R*_0_ values (relative [Ca^2+^] and relative [ROS]). Data represent the mean ± SD (*n* = 41). **A** The caspase-8 inhibitor, zIETD-fmk (50 μM) and the pan-caspase inhibitor z-VAD-fmk (100 μM) were pre-incubated for 30 min with AAV neutrophils, and then sCD95L was added (100 ng/mL). Cytosolic Ca^2+^ concentration was measured. For each condition, data represent mean ± SD of *n* = 41 cells. Data are representative of three independently performed experiments. **B** The caspase-8 inhibitor, zIETD-fmk (50 μM) and the pan-caspase inhibitor, z-VAD-fmk (100 μM) were pre-incubated for 30 min with AAV neutrophils, and then sCD95L was added (100 ng/mL). Cytosolic ROS concentration was measured. For each condition, data represent mean ± SD of *n* = 41 cells. Data are representative of three independently performed experiments. **C** TNF- (Left panel) and IL-1β- (Right panel) primed HD neutrophils (5 ng/mL, 15 minutes) were pre-incubated with zIETD-fmk (50 μM) for 30 minutes and then exposed to sCD95L (100 ng/mL). The cytosolic ROS response was assessed. For each condition, data represent mean ± SD of *n* = 41 cells. Data are representative of three independently performed experiments. **D** The caspase-8 inhibitor, zIETD-fmk (50 μM), was pre-incubated for 30 min with AAV neutrophils and then sCD95L was added (100 ng/mL). Mitochondrial Ca^2+^ concentration was measured. For each condition, data represent mean ± SD of *n* = 41 cells. **E** zIETD-fmk (50 μM) was pre-incubated for 30 min with AAV neutrophils, and then sCD95L was added (100 ng/mL). Mitochondrial ROS concentration was measured. For each condition, data represent mean ± SD of *n* = 41 cells. **F** The indicated peptide in OTULIN (orange) encompasses an aspartic acid (D) and was identified as lost in HL60 exposed to sCD95L and IgCD95L and restored when cells were pre-incubated with the pan-caspase inhibitor zVAD-fmk.

In agreement with our previous observation^25^, inhibition of caspase activity with either the selective caspase-8 inhibitor zIETD-fmk or the pan-caspase inhibitor zVAD-fmk did not affect the CD95-induced cytoplasmic Ca^2+^ response (Fig. 4A). Nonetheless, both inhibitors abrogated ROS production in AAV neutrophils stimulated with sCD95L (Fig. 4B and Supplementary Fig. 4A). Similarly, CD95 activation in TNF- and IL-1β-primed HD neutrophils triggered caspase-dependent ROS production (Fig. 4C). These findings indicated that AAV neutrophils exposed to sCD95L underwent caspase-dependent ROS production. Because the CD95-mediated Ca^2+^ signal was mandatory for mitochondrial ROS production in AAV neutrophils (Supplementary Fig. 3C and Supplementary Fig. S3D) and cytosolic Ca^2+^ was not affected by caspase inhibitors, we hypothesized that the mitochondrial Ca^2+^ load was the target of caspase activity. In agreement with this hypothesis, CD95-mediated mitochondrial Ca^2+^ load in AAV neutrophils was inhibited by zIETD-fmk pretreatment (Fig. 4D). zIETD-fmk also inhibited CD95-mediated ROS production by mitochondria in AAV neutrophils (Fig. 4E), indicating that caspase activity in AAV neutrophils contributed to the induction of the Ca^2+^ load in the mitochondria and subsequent ROS production.

To identify caspase substrates involved in CD95-mediated ROS production, we conducted a comprehensive proteomic assay. To reduce inter-donor genetic variabilities and compare more easily each replicate, we performed this analysis using a human promyelocytic HL-60 cell line that can differentiate into neutrophil-like cells using a low concentration of dimethyl sulfoxide (DMSO)^42^. We validated that AAV serum induced mitochondrial Ca^2+^ uptake (Supplementary Fig. 4B) and ROS production (Supplementary Fig. 4C) in DMSO-differentiated HL60 cells, and these signals were abrogated by NOK-1 and DB550 (Supplementary Fig. 4B, C). Both mitochondrial Ca^2+^ uptake (Supplementary Fig. 4D) and ROS production (Supplementary Fig. 4E) were abrogated by zIETD treatment in DMSO-differentiated HL60 cells. Finally, Bcl2 and VDAC1 inhibitors, ABT-263 and L14-15, also blocked the mitochondrial Ca^2+^ uptake (Supplementary Fig. 4F) and mitochondrial ROS production (Supplementary Fig. 4G) in DMSO-differentiated HL60 cells exposed to sCD95L. These findings indicated that, similar to primary neutrophils, DMSO-differentiated HL60 cells exposed to sCD95L underwent Bcl2/VDAC1 and caspase-driven mitochondrial Ca^2+^ loading, which in turn promoted ROS production.

Because mitochondrial Ca^2+^ load and ROS production occurred rapidly after incubation with sCD95L, a proteomic assay was performed using DMSO-differentiated HL60 cells stimulated for a short period of time (30 min) (Supplementary Data 1; doi:10.6084/m9.figshare.26300509). A similar approach was performed using IgCD95L, a homemade dodecameric ligand^43^, which induced a strong caspase-dependent cell death signal (Supplementary Data 1; doi:10.6084/m9.figshare.26300509). We selected HL60 cells because they recapitulated the whole signaling pathway observed in AAV neutrophils and primary neutrophils pre-activated with IL1β or TNF, and their use minimized the inter-donor or inter-patient variability, which could have complicated the proteomic data analyses. We first monitored whether HL60 cells exposed to different CD95 stimuli underwent an apoptotic signal, using an early marker of cell death, the loss of mitochondria depolarization (Δψm)^44^. We confirmed that up to 1 hour, no drop of Δψm was observed in neutrophils exposed to a weak (sCD95L) or a strong (IgCD95L) CD95 stimulus (Supplementary Fig. 5A) and only after 24 hours, IgCD95L triggered apoptosis in HL60 cells (Supplementary Fig. 5A). These data indicated that the caspase activation induced by both sCD95L and IgCD95L-mediated signals did not kill HL60 cells during the proteomic analysis. Second, to identify caspase substrates involved in CD95-mediated ROS production, we selected peptides that were lost in neutrophils exposed to two types of CD95 stimulation - IgCD95L and sCD95L - but whose expression was restored by zVAD-fmk pretreatment (proteomic data from unstimulated cells and from cells stimulated with IgCD95L or sCD95L, with or without zVAD-fmk, are shown in Supplementary Data 1; doi:10.6084/m9.figshare.26300509). Next, from this set of peptides, we retained only those containing an aspartic acid (D) residue (Supplementary Fig. 5B and Supplementary Data 2), as caspases are known to cleave after this amino acid^45^. Finally, using the BioVenn software^46^, we identified peptides common to both sCD95L- and IgCD95L-treated conditions and further restricted the analysis to those whose expression was lost in the presence of sCD95L and IgCD95L stimuli and restored in the presence of zVAD-fmk (Supplementary Fig. 5B and Supplementary Data 2). This stringent analysis revealed that only two peptides, derived from STXBP3 and OTULIN, were targets of CD95-induced caspase activity in the HL60 cell line (Supplementary Fig. 5B and Supplementary Data 2).

OTULIN is a potent regulator of inflammation^47^, and the peptide analysis revealed that the amino acid sequence 35 to 57 of OTULIN was lost in HL-60 cells exposed to sCD95L or IgCD95L, suggesting that this region, harboring an aspartic acid at position 54 (D54, Fig. 4F), could be cleaved by caspases.

### Caspase-10 cleaves OTULIN at D54 in neutrophils

**Fig. 5:**
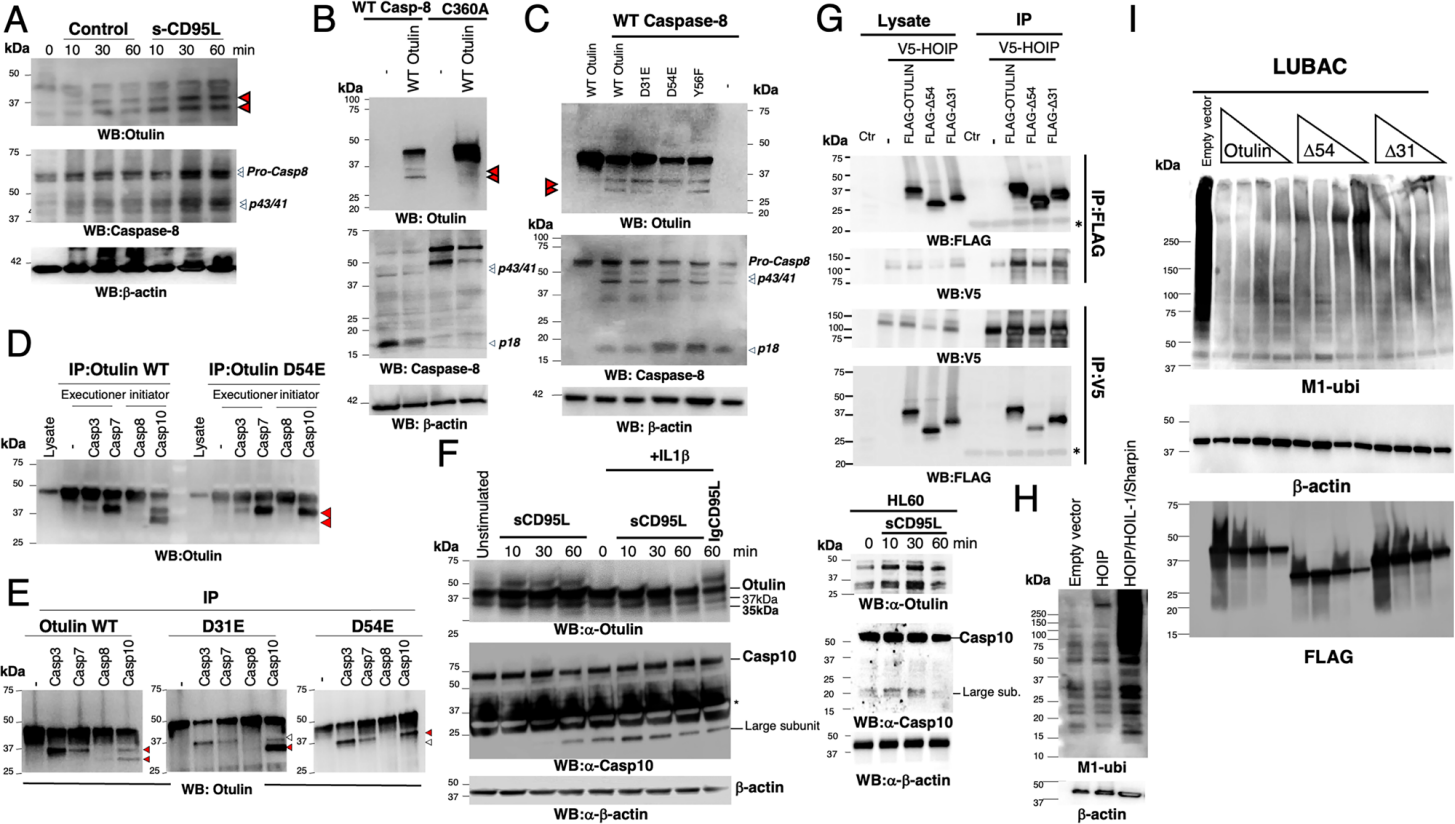
Caspase-10-mediated cleavage of OTULIN at D54 in neutrophils. **A** DMSO-differentiated HL60 cells were stimulated with sCD95L (100 ng/mL) for the indicated times. Cell lysates were analyzed by immunoblotting; β-actin served as a loading control. **B** HEK293T cells co-transfected with wild-type (WT) or inactive (C360A) caspase-8 with WT OTULIN were analyzed 24 h post-transfection. Activation of WT caspase-8 induced OTULIN cleavage, generating two fragments ( ~ 37 and ~ 35 kDa) (red arrows), whereas C360A did not. Caspase-8 activation is monitored by the detection of processed p43/p41 and p18 fragments. These cleavages only occured for WT. **C** HEK293T cells were co-transfected with WT caspase-8 and indicated OTULIN mutants. The ~ 35 kDa cleavage product observed with WT OTULIN was abolished with the D54E mutant. **D** Flag-tagged WT OTULIN or D54E mutant was immunoprecipitated with anti-Flag-coated beads and incubated with indicated recombinant caspases for 90 min, followed by SDS–PAGE and immunoblotting. The ~ 35 kDa cleavage product (red arrows) was detected for WT OTULIN incubated with active caspase-10 but not for D54E. **E** Flag-tagged WT, D54E, or D31E OTULIN constructs were processed as in (D). The two cleavage products (red arrows) observed for WT OTULIN incubated with caspase-10 were reduced or absent in mutants. **F** Left: Primary human neutrophils, pre-treated or not with IL-1β, were stimulated with sCD95L or IgCD95L (100 ng/mL). Right: HL60 cells were treated with sCD95L. Lysates were analyzed by immunoblotting; β-actin served as a loading control. **G** HEK293T cells co-transfected with V5-HOIP and Flag-OTULIN constructs were subjected to anti-V5 or anti-FLAG immunoprecipitation followed by immunoblotting to assess interactions. Input and immunoprecipitates are shown. **H** HOIP alone or the whole LUBAC complex (HOIP/HOIL-1/Sharpin) was expressed in HEK293T cells, and linear ubiquitination (M1-ubi) was analyzed by immunoblotting 48 h post-transfection. **I** HEK293T cells were co-transfected with LUBAC components and increasing amounts of Flag-OTULIN cDNA (3, 1, 0.3 or 0.1 μg). Lysates were analyzed by immunoblotting. All data are representative of three independent experiments.

To further investigate whether the deubiquitinase (DUB) OTULIN was involved in CD95-mediated mitochondrial ROS production, DMSO-differentiated HL60 cells were stimulated with sCD95L, and we observed that in addition to the full-length OTULIN ( ~ 45 kDa), two additional bands (∼ 37 and 35 kDa) appeared (Fig. 5A), suggesting that DUB could undergo two cleavages in cells exposed to sCD95L. We also observed activation of the initiator caspase-8 in these cells when stimulated with sCD95L, with the appearance of p43/p41 fragments (Fig. 5A). Although to a lesser extent and in agreement with pioneering studies^48^, low caspase activity was observed in DMSO-differentiated HL60 cells, which could account for the basal OTULIN cleavage observed in untreated cells (Fig. 5A).

Since the caspase-8 inhibitor, zIETD-fmk, inhibited CD95-mediated ROS production in neutrophils exposed to sCD95L (Fig. 4B), we postulated that this initiator caspase could be involved in OTULIN cleavage. Therefore, we co-expressed wild-type (WT) caspase-8 and OTULIN in HEK/293 T cells (Fig. 5B, C). Caspase-8 overexpression in HEK/293 T cells resulted in its catalytic autoactivation, with the appearance of a large subunit fragment (∼ 18 kDa) (Fig. 5B, C) and the induction of an apoptotic signal that was inhibited by zVAD-fmk (Supplementary Fig. 5C). No large subunit fragment (Fig. 5B, C) and apoptotic signal (Supplementary Fig. 5C) were detected when cells were transfected with the catalytically inactive caspase-8 mutant (C360A) as previously described^49^. WT caspase-8 overexpression in HEK/293 T cells induced double cleavage of WT OTULIN (∼37 and 35 kDa) (Fig. 5B), similar to that observed in DMSO-differentiated HL60 cells exposed to sCD95L (Fig. 5A). Co-expression of OTULIN with the C360A mutant did not result in any cleavage of WT OTULIN (Fig. 5B). To confirm that OTULIN was cleaved at residue D54, we generated a mutant in which aspartic acid 54 was replaced with glutamic acid (D54E). We compared its cleavage to that of Y56F, a mutant that prevents OTULIN/HOIP interaction^50^ and the D31E mutant, a cleavage site identified in cells exposed to TNF/CHX or UVB^51^. Caspase-8 overexpression in HEK/293 T cells led to the double cleavage of D31E and Y56F mutants, while the 35 kDa band was lost with the D54E mutants (Fig. 5C). These findings confirm that aspartic acid at position 54 of OTULIN is targeted by caspase 8, or downstream activated caspases. Notably, this cleavage occurred independently of the OTULIN/HOIP interaction, as the Y56F mutant was still cleaved upon the transfection of caspase-8 in HEK/293 T cells.

Because the caspase-8 overexpression in HEK/293 T cells triggered apoptosis (Supplementary Fig. 5C), which relies on initiator and executioner caspases, this assay did not allow us to precisely identify the caspase involved in OTULIN cleavage. To tackle this question, we developed a cell-free assay in which activated and purified caspases were mixed with purified WT, D31E, and D54E OTULIN constructs. For this purpose, a FLAG tag was fused to the N-terminal end of WT, D54E, and D31E OTULIN, the constructs were transfected into HEK/293 T cells, and immunoprecipitated. The purified OTULIN constructs were then mixed with the activated initiator caspase-8 and caspase-10 involved in CD95-mediated DISC formation^19^ or the downstream executioner caspases -3 and -7 (Fig. 5D, E). The effect of each caspase on the OTULIN constructs was analyzed by immunoblotting (Fig. 5D, E). While caspases -3 and 7 generated a single cleaved band at 37 kDa (Fig. 5D and Supplementary Fig. 5D, E), caspase-10 generated two bands (37 and 35 kDa), as observed in DMSO-differentiated HL60 cells exposed to sCD95L (Fig. 5A) and in caspase-8-transfected HEK/293 T cells (Fig. 5B, C). Strikingly, purified caspase-8 failed to cleave OTULIN (Fig. 5D, E). Although the OTULIN D54E mutant did not affect caspase-3 and -7 cleavage (Fig. 5D and Supplementary Fig. 5D, E), it prevented the generation of a 35 kDa band in the presence of caspase-10 (Fig. 5D, E). Similarly, the D31E mutant did not affect caspase-3 and -7 cleavage (Fig. 5E and Supplementary Figs. 5D, E), but impaired the generation of the 37 kDa band in the presence of caspase-10 (Fig. 5E and Supplementary Figs. 5E). These findings indicated that caspase-10 cleaves OTULIN at D31 and D54.

Because the D31E mutant did not prevent caspase-3 and 7 cleavages, but impinged on caspase-10 cleavage (Fig. 5D, E), we hypothesized that either this mutation was not efficient in preventing caspase-3 and -7 cleavage, as previously reported for different caspase substrates^45^ or these caspases cleaved OTULIN in its C-terminal region, engendering a cleaved DUB, migrating at the same size as the D31 cleavage. To address this, we took advantage of the FLAG tag at the N-terminal end of the OTULIN construct. Anti-FLAG immunoblots did not reveal an additional OTULIN band at 37 kDa in the presence of caspases -3 and -7 (Supplementary Fig. 5E), indicating that this DUB was not processed at its C-terminal end and it was probably cleaved at D31 and that the replacement of this aspartic acid by a glutamic acid residue did not affect its processing by caspases-3 and -7.

We next wondered whether caspase-10 was activated in primary neutrophils isolated from healthy donors and in DMSO-differentiated HL60 exposed to sCD95L (Fig. 5F). The activation of caspase-10 and the appearance of its large subunit was detected at 60 min in neutrophils from healthy donor (Fig. 5F). Interestingly, a basal amount of the caspase-10 large subunit was detected in unstimulated IL1β-primed neutrophils and this cleavage reached a peak of activation at 10 min after the addition of sCD95L (Fig. 5F). This increased in caspase-10 activation in IL1β-primed neutrophils could account for the stronger ROS/Ca^2+^ responses observed in these cells as compared to unprimed cells (Figs. 2E, 3D). Like sCD95L, the multi-aggregated IgCD95L construct^43^ triggered the cleavage of OTULIN (Fig. 5F). Similarly, a rapid caspase-10 activation occurred in DMSO-differentiated HL60 exposed to sCD95L (Fig. 5F), reaching a peak of activation similar to that observed in IL1β-primed neutrophils and paralleled to the cleavage of OTULIN (Fig. 5F). Although a basal level of the 37 kDa band was detected in untreated neutrophils, the intensity of 35 and 37 kDa bands increased when cells were exposed to sCD95L (Fig. 5F).

Overall, these findings combined with the cell-free caspase assay provided an explanation as to why the expression of the D31E mutant in caspase-8-transfected HEK/293 T cells did not prevent the generation of the upper cleaved band (Fig. 5C), because by activating downstream caspases -7 and/or -3 in these cells, caspase-8 overexpression engenders the 37 kDa fragment, covering the loss of caspase-10 cleavage at D31 (Fig. 5E).

To strengthen these data, we next evaluated whether caspase-10 and OTULIN were able to interact by using a protein-fragment complementation assay (PCA). Briefly, we split *Renilla* luciferase into two fragments (F1 and F2)^52^ and fused them to human caspase-8, caspase-10, or OTULIN. As caspase-8 can interact with itself through its death effector domain (DED)^53,54^, we used it as a positive control (Supplementary Fig. 5F). The co-expression of OTULIN with caspase-10 fused to F1 or F2 domains in HEK/293 T cells (Supplementary Fig. 5G) promoted the refolding of *Renilla* luciferase and subsequent light emission (Supplementary Fig. 5F), confirming the caspase-10/OTULIN interaction.

D54 is localized within the PUB interacting motif (PIM) domain of OTULIN (Fig. 4F) involved in HOIP interaction^55^, suggesting that caspase-10 cleavage could affect the OTULIN/HOIP interaction. We generated two truncated OTULIN constructs in which we deleted the first 31 (Δ31-OTULIN) and 54 amino acids (Δ54-OTULIN). Next, we co-transfected HEK/293 T cells with V5-tagged HOIP and WT, Δ31-, or Δ54-OTULIN fused to FLAG, and performed co-immunoprecipitation (co-IP) experiments (Fig. 5G). Anti-FLAG immunoprecipitation of WT and Δ31-OTULIN efficiently coimmunoprecipitated with V5-HOIP (Fig. 5G. In contrast, although the quantity of immunoprecipitated Δ54-OTULIN was similar to that of the WT and Δ31 constructs, less HOIP co-immunoprecipitated with this construct (Fig. 5G). In agreement with these data, HOIP immunoprecipitation revealed a reduced interaction with Δ54-OTULIN compared with the WT and Δ31 constructs (Fig. 5G). Although methionine 55 and tyrosine 56 within OTULIN were reported to be two key amino acids interacting with the HOIP PUB domain^55^ and these amino acids were conserved in our Δ54 construct, caspase-10-mediated cleavage after D54 decreased in the OTULIN/HOIP interaction, thereby altering LUBAC regulation. To test this hypothesis, we analyzed M1 linear ubiquitination in the presence of WT, Δ31 and Δ54 OTULIN. Although HOIP by itself performs Met1-linked ubiquitination^56^ (Fig. 5H), this activity was increased when combined with HOIL-1 and SHARPIN to form the LUBAC tripartite complex^57^ (Fig. 5H). Therefore, the ubiquitin ligase activity of LUBAC was analyzed in the presence of WT, Δ31-, or Δ54-OTULIN (Fig. 5I). A dose effect of WT, Δ31 and Δ54 OTULIN constructs revealed that WT and Δ31 OTULIN abrogated LUBAC-mediated M1 ubiquitination (Fig. 5I), while the Δ54 protein less efficiently counteracted the linear ubiquitination (Fig. 5I). Indeed, a densitometry analysis of the M1-ubiquitination in HEK cells co-transfected with HOIP and OTULIN constructs indicated that while WT and Δ31-OTULIN efficiently down-regulated the HOIP-mediated M1-ubiquitination, Δ54-OTULIN reduced it only by ∼ 50% (Fig. 5I). In summary, these data established that the caspase-10-driven cleavage of OTULIN after its aspartic acid at position 54 engenders a truncated DUB failing to interact with HOIP and down-regulating LUBAC activity.

### Caspase-10 activity is involved in CD95-mediated ROS and Ca^2+^ signaling

**Fig. 6:**
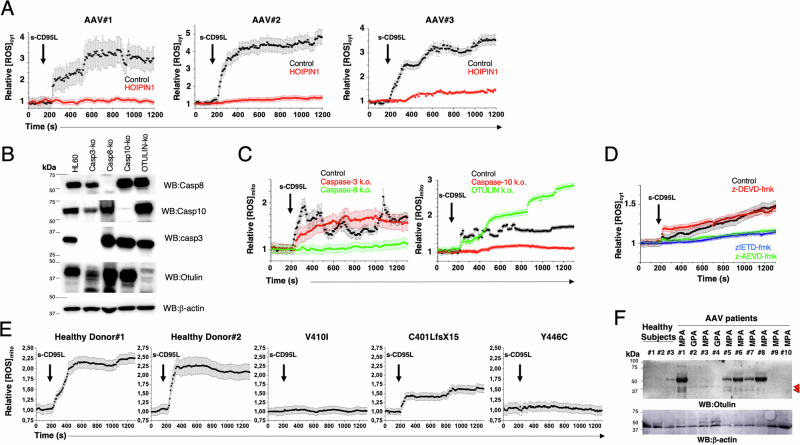
Caspase-10-mediated ROS production in neutrophils exposed to sCD95L. **A** Neutrophils isolated from three AAV patients were pre-incubated in the presence or absence of HOIPIN-1 (10 μM), then the cells were stimulated with sCD95L (100 ng/mL) and cytosolic ROS production was monitored with the fluorescent probe H2DCFDA. Ratio values (*R*) were normalized to pre-stimulated values (*R*_0_) to yield *R*/*R*_0_ values (relative [ROS]). For each condition, data represent mean ± SD of *n* = 41 cells. **B** Wild type (control), caspase-10 and OTULIN k.o. HL60 cells were lysed and loaded in a 12% SDS-PAGE. The indicated immunoblots were performed. β-actin served as a loading control. Data are representative of at least three independently performed experiments. **C** DMSO-treated wild type (mock), caspase-8, caspase-10, OTULIN or caspase-3 k.o. HL60 cells were stimulated with sCD95L (100 ng/mL), and the mitochondrial ROS ([ROS]_mito_) was assessed using MitoSOX. For each condition, data represent mean ± SD of *n* = 41 cells. Data are representative of three independently performed experiments. **D** IL-1β/TNF-primed neutrophils isolated from HD were pre-incubated for 30 min with low concentration (1 μM) of the indicated caspase inhibitors and then stimulated with sCD95L (100 ng/mL). Cytosolic ROS concentration was assessed with the fluorescent probe H2DCFDA. For each condition, data represent mean ± SD of *n* = 41 cells. Data are representative of three independently performed experiments. **E** Herpesvirus saimiri-immortalized T-cells isolated from ALPS-CASP10 patients were stimulated with sCD95L (100 ng/mL). Cytosolic ROS concentration was assessed with the fluorescent probe H2DCFDA. For each condition, data represent mean ± SD of *n* = 41 cells. Data are representative of three independently performed experiments. **F** Neutrophils were isolated from AAV patients and healthy subjects and lysed. Proteins were resolved in a 12% SDS-PAGE, and an OTULIN immunoblot was performed. Patients 1, 5, 6, 7 and 8 showed an up-regulation of OTULIN and its cleavage. β-actin serves as a loading control. Data are representative of three independently performed experiments.

Because OTULIN interacts with the ubiquitin ligase LUBAC to modulate its activity, we investigated whether caspase-10-mediated OTULIN cleavage in neutrophils released LUBAC, and whether this could be implicated in CD95-mediated ROS production in neutrophils. HOIPIN-1^58^, a selective inhibitor of the ubiquitin ligase activity of LUBAC, did not alter the CD95-mediated cytosolic Ca^2+^ signal in AAV neutrophils (Supplementary Fig. 6A), but abrogated cytosolic and mitochondrial ROS production, as well as mitochondrial Ca^2+^ (Fig. 6A and Supplementary Fig. 6A). Similar data were obtained with HOPIN-1 in IL-1β and TNF-primed neutrophils isolated from healthy donors (Supplementary Fig. 6B), confirming that the caspase-10-mediated OTULIN cleavage released LUBAC activity to promote the mitochondrial Ca^2+^ load and ROS production.

To further investigate the roles of caspase-10 and OTULIN in CD95-mediated ROS production in neutrophils, we generated caspase-10 and OTULIN-knockout (k.o.) HL60 cells using CRISPR/Cas9 (Fig. 6B). Elimination of caspase-10 in HL60 cells abrogated CD95-mediated ROS production (Fig. 6C). Similarly, caspase-8 k.o. HL60 cells failed to trigger mitochondrial ROS production in the presence of sCD95L (Fig. 6C), confirming that this caspase is probably upstream of caspase-10 in the CD95-mediated ROS signaling pathway. In contrast, elimination of caspase-3 did not affect the oxidative burst (Fig. 6C). Interestingly, the elimination of OTULIN in HL60 cells exposed to sCD95L generated a stronger ROS signal than control cells (Fig. 6C), supporting that OTULIN plays a negative function in this signaling pathway. In agreement with this genetic approach, pharmacological inhibition of caspase-10 (*i.e*., zAEVD-fmk) or caspase-8 (*i.e*., zIETD-fmk) also abrogated CD95-mediated ROS production (Fig. 6D), whereas the caspase-3/7 (*i.e*., zDEVD-fmk) inhibitor did not (Fig. 6D).

To further strengthen the evidence for the role of caspase-10 in CD95-mediated mitochondrial ROS signaling, we analyzed ROS production using herpes virus saimiri (HVS)-immortalized T cells generated from patients with ALPS-CASP10^59^ carrying Y446C, V410I, or C401LfsX15 homozygous caspase-10 mutations^60^. Mitochondrial ROS production observed in healthy donor T cells exposed to sCD95L (Fig. 6E) was dramatically impaired in T cells expressing mutated caspase-10 (Fig. 6E), supporting the notion that the CD95-mediated oxidative burst relied on a caspase-10-driven mechanism.

Finally, we evaluated whether neutrophils isolated from patients with AAV (Supplementary Table 3) showed OTULIN cleavage (Fig. 6F). We assessed the DUB status in AAV neutrophils and found that OTULIN expression was upregulated mainly in active MPA compared to patients with GPA (Fig. 6F), and that double cleavage occurred in these patients (Fig. 6F). These findings confirmed that the OTULIN signaling pathway was likely deregulated in neutrophils isolated from patients with MPA and that CD95-mediated cleavage of this DUB could hinder its inhibitory role in the oxidative burst.

### DB550 treatment alleviated clinical symptoms in AAV mouse model

**Fig. 7:**
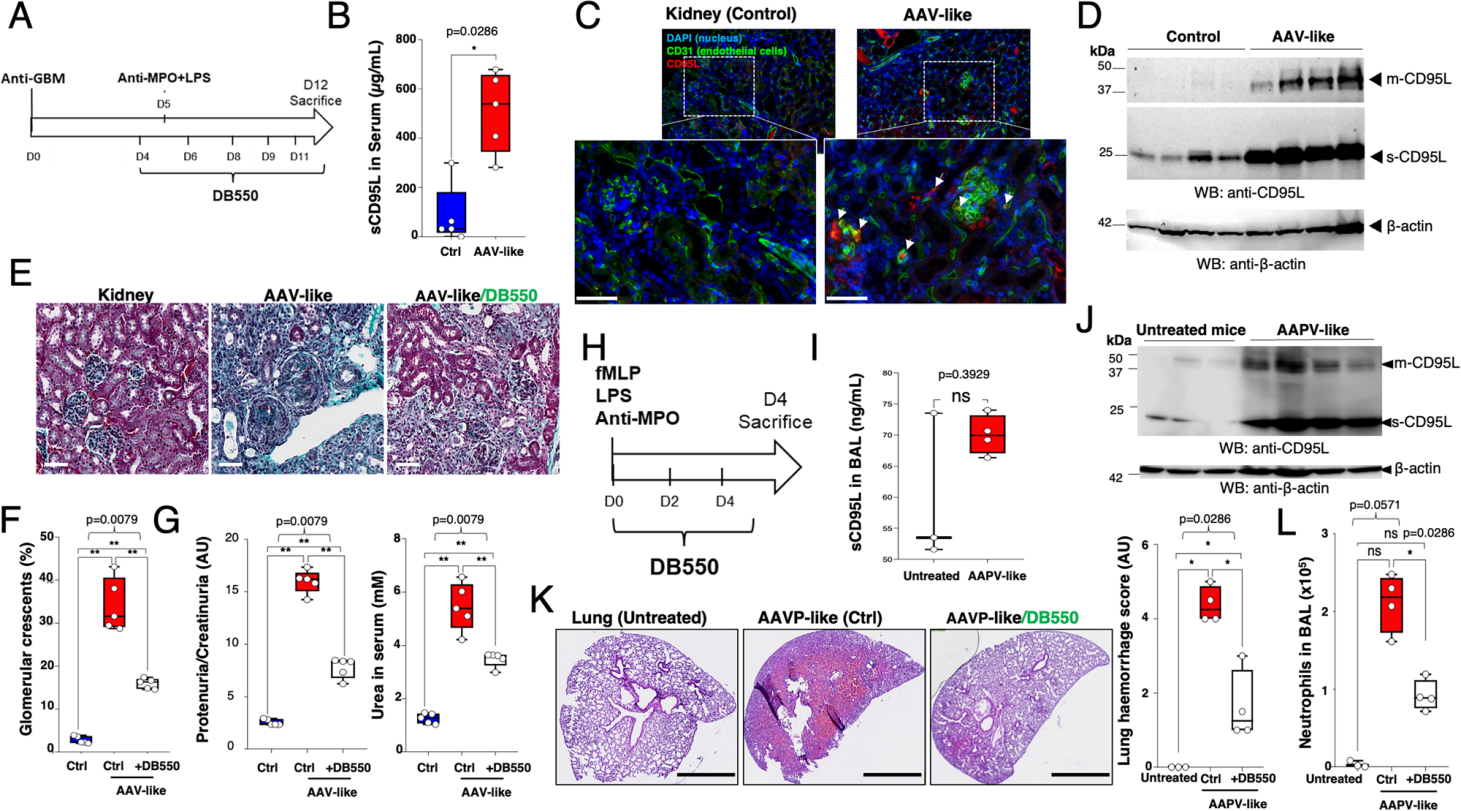
DB550 treatment alleviates clinical symptoms in two AAV-like mouse models. **A** Anti-MPO-induced ANCA vasculitis model. Mice (AAV-like, Ctrl) received anti-GBM serum (40 mg/kg, day 0) followed by anti-MPO (30 mg/kg) and LPS (0.5 mg/kg, day 5). Untreated controls received non-immune serum, isotype IgG, and PBS. AAV-like mice were treated or not with DB550 (10 mg/kg, three times weekly) (*n* = 5/group). **B** Serum soluble CD95L levels were quantified by ELISA at day 12 (mean ± SD; *p < 0.05, two-tailed Mann-Whitney). **C** Localization of CD95L (red) and CD31 (green) in kidney sections of AAV and control mice; nuclei stained with DAPI (blue). Scale bar, 50 µm. **D** Membrane-bound (48 kDa) and soluble (26 kDa) CD95L in kidney lysates were analyzed by immunoblotting. **E** Kidney histology assessed by Masson’s trichrome staining. Scale bar, 50 µm. **F** Glomerular injury (% crescents) was scored (mean ± SD; **p < 0.01, two-tailed Mann-Whitney; *n* = 5/group). **G** Renal function assessed by urine protein/creatinine ratio and serum urea (mean ± SD; **p < 0.01, two-tailed Mann-Whitney; *n* = 5/group). **H** ANCA pulmonary vasculitis (AAPV) model. Mice received intratracheal LPS (0.5 mg/kg) and fMLP (0.5 mg/kg), followed by anti-MPO (50 mg/kg). Controls received PBS and isotype IgG. AAPV mice were treated or not with DB550 (10 mg/kg, at D-1 and D3) (*n* = 3-4/group). **I** Soluble CD95L levels in bronchoalveolar lavage (BAL) were measured by ELISA at day 12 (mean ± SD; ns for not significant, two tailed Mann-Whitney). **J** Membrane-bound and soluble CD95L in lung lysates were analyzed by immunoblotting. **K** Left: Lung histology by hematoxylin/eosin staining (scale bar, 2 mm). Right: Lung hemorrhage scores (mean ± SD; *p < 0.05, two tailed Mann-Whitney; *n* = 3-4/group). **L** Neutrophil counts in BAL (mean ± SD; ns or *p < 0.05, two-tailed Mann-Whitney; *n* = 3-4/group). All data are representative of at least three independent experiments unless stated otherwise.

We first evaluated whether selective inhibition of the CD95-mediated non-apoptotic signal by DB550, a selective inhibitor of the CD95/PLCγ1 interaction^27^, could dampen the clinical symptoms in an anti-MPO-induced ANCA kidney vasculitis mouse model (AAV-like) (Fig. 7A)^61^. These mice developed focal necrotizing glomerulonephritis that progressed to a kidney architecture comparable to that observed in human AAV^61^. Like in AAV patients, the pathology induction in these mice engendered an increase in serum CD95L (Fig. 7B). In addition, kidneys from these mice exhibited an increase in CD95L (Fig. 7C), which seemed to be co-localized with endothelial cells (*i.e*., CD31^+^ cells) in the glomeruli and most of the blood vessels. Kidney lysates of AAV-like mice confirmed the ectopic expression of membrane-bound CD95L compared to that of untreated mice (Fig. 7D). This ligand was cleaved, resulting in the accumulation of its soluble counterpart resolved at 25 kDa (Fig. 7D). Analyses of kidney architecture revealed that DB550 treatment in AAV-like mice improved kidney architecture (Fig. 7E, F). Masson’s trichrome staining, which labels collagen fiber deposition, indicated that DB550-treated mice experienced a dramatic reduction in fibrosis (appearing blue upon Masson’s trichrome staining) compared to AAV-like mice (Ctrl) (Fig. 7E). Since the urinary protein-to-creatinine ratio reflects necrotizing and crescentic GN and AAV disease severity^62^, we used this parameter to estimate the therapeutic effect of DB550 in mice with ANCA vasculitis. This ratio was lower in the DB550-treated mice than in untreated AAV-like mice (Ctrl) (Fig. 7G). A comprehensive analysis of the immune landscape in the spleen of these animals revealed that only the percentage of neutrophils significantly increased in glomerulonephritis AAV animals as compared to untreated mice (gating strategy shown in Supplementary Fig. 7 and data depicted in Table 1), and this population was downregulated by the DB550 treatment (Table 1). These findings indicated that the selective inhibition of the CD95-induced non-apoptotic signal altered the trafficking and/or the sensitivity of these cells. Finally, both, neutrophil elastase staining (Supplementary Fig. 8A) and the dosage of lipocalin 2 (neutrophil gelatinase-associated lipocalin 2) (Supplementary Fig. 8B), confirmed that DB550 treatment reduced neutrophil recruitment in the kidney tissue compared to untreated AAV-like mice (Ctrl). The clinical improvement in DB550-treated mice was also associated with the recovery of body weight in DB550-injected animals compared with vehicle-treated counterparts (Supplementary Fig. 8C).

**Table 1:** Immune cell proportions in control, AAV, and AAV **+ **DB550-treated mice

		Control *n *= 3		AAV* n *= 3			AAV+ DB550 n=3		
Population	Gating strategy	Mean (%)	SD	Mean (%)	SD	*P*-Value	Mean (%)	SD	*P*-Value
Monocytes	CD19- CD3- CD11b + Ly6G- Ly6C + among CD45 +	0.7033	0.09866	0.7767	0.1518	> 0,9999	0.7833	0.07371	0.6611
Inflammatory Monocytes	CD19- CD3- CD11b + Ly6G- Ly6Chi among CD45 +	0.36	0.1015	1.903	0.4315	0.0738	1.83	0.2982	0.1053
Neutrophils	CD19- CD3- CD11b + Ly6G + LY6C- among CD45 +	0.63	0.3637	6.083	1.438	***0.0141***	3.907	0.185	0.3558
Conventional dendritic cells (cDC)	CD19- CD3- CD11chi MHCIIhi among CD45 +	1.52	0.5129	2.08	0.3132	0.3594	1.77	0.3274	> 0.9999
cDC1	CD19- CD3- CD11chi MHCIIhi CD11b + among CD45 +	0.64	0.2443	0.8067	0.1422	0.5231	0.7033	0.1484	> 0.9999
cDC2	CD19- CD3- CD11chi MHCIIhi CD11b- among CD45 +	0.88	0.2689	1.273	0.171	0.1993	1.063	0.1779	0.8206
Plasmacytoid DC	CD19- CD3- CD11c + MHCIIlow Ly6C + among CD45 +	0.1967	0.05508	0.44	0.1453	0.1053	0.3967	0.1815	0.2021
Follicular B cells	CD23 + CD21/35 + among CD19 +	81.03	1.06	81.13	2.635	> 0.9999	79.17	1.361	0.3594
Marginal zone B cell	CD23- CD21/35hi among CD19 +	10.54	1.909	1.909	2.248	0.1053	16.5	1.587	0.0738
Central memory CD4 T cell	CD44 + CD62L + among CD4 +	39.57	17.84	21.77	4.823	0.7422	16.67	3.536	0.0507
Effector memory CD4 T cell	CD44 + CD62L- among CD4 +	14.63	2.316	15.63	1.553	> 0.9999	14.13	1.002	> 0.9999
Naive CD4 T cell	CD44- CD62L + among CD4 +	33.9	8.302	35.47	1.501	0.7422	29.73	0.9504	0.7422
TH17	RORgt + among CD4 +	9.337	1.975	6.653	1.718	0.5934	4.96	0.03	0.0225
Treg	FoxP3 + among CD4 +	12.83	1.724	18.23	3.323	0.1053	16.87	1.888	0.2021
Central memory CD8 T cell	CD44 + CD62L +among CD8 +	26.8	5.209	21.27	3.182	0.4661	22.93	3.623	0.5934
Effector memory CD8 T cell	CD44 + CD62L- among CD8 +	1.85	0.4694	3.857	2.225	0.2021	3.817	0.1779	0.2721
Naive CD8 T cell	CD44- CD62L + among CD8 +	67.57	3.329	64.37	8.043	0.7422	59.53	5.229	0.1473

The relative proportions of splenic immune cell subsets were determined by multiparameter flow cytometry. Immune populations were defined according to the gating strategy described in the Methods. Statistical analysis was performed using a non-parametric one-way ANOVA followed by Dunn’s multiple-comparisons test. Indicated *P-*values correspond to comparisons *versus* control mice. Data are presented as mean ± SD. Bold italic values denote statistically significant differences. Experiments were performed independently three times.

To further investigate the therapeutic effect of DB550 in an aggressive ANCA lung disease, we developed an anti-MPO-induced ANCA pulmonary vasculitis (AAPV-like), mimicking symptoms of severe disease in patients (Fig. 7H)^63^. sCD95L showed a dramatic increase in the bronchoalveolar lavage (BAL) of AAV pulmonary mice as compared to untreated animals but this difference was not significant due to one outlier untreated mouse (Fig. 7I). As shown in Fig. 7J, the lysates of lungs isolated from AAPV mice exhibited a strong up-regulation of m- and sCD95L expression. The lung hemorrhages in AAPV-like mice (Ctrl) associated with diffuse alveolar damages were dramatically dampened by DB550 treatment (Fig. 7K). In addition, the recruitment of neutrophils in the broncho-alveolar space was significantly reduced in DB550-treated AAPV mice as compared to untreated AAPV-like mice (Ctrl) (Fig. 7L). In agreement with this, lipocalin 2, a marker of neutrophil activation, decreased in the lungs of DB550-treated compared to control AAPV-like mice (Supplementary Fig. 8D).

Finally, the “anti-GBM” mouse model is used as an AAV-like mouse model, despite the fact that this animal model does not recapitulate all clinical symptoms observed in ANCA-associated crescentic glomerulonephritis (cGN)^6^.

Nonetheless, this mouse model allows us to evaluate the therapeutic effect of DB550 on the late stages of ANCA vasculitis, meaning the formation of pauci-immune focal segmental cGN^6^ (Supplementary Fig. 9A). In this model, the accumulation of antibodies in the kidneys results in complement activation and Th17 trafficking, promoting neutrophil recruitment, and causing glomerular damage and renal dysfunction^64^. Serum CD95L levels increased in these anti-GBM treated mice compared to that in untreated mice (Supplementary Fig. 9B). In agreement with human observations, CD95L was expressed by endothelial cells in damaged kidneys (Supplementary Fig. 9C). To evaluate whether the CD95/CD95L axis participates in pathological progression in this animal model, we administrated DB550^27^. Because the DB550-interacting SH3-PLCγ1 domain is conserved between human and mouse (Supplementary Fig. 9D), DB550 inhibited the cytosolic Ca^2+^ response in mouse “anti-GBM” neutrophils exposed to sCD95L (Supplementary Fig. 9D). Although rodents do not express caspase-10, we confirmed that the caspase-8 inhibitor blocked ROS production in mouse neutrophils exposed to sCD95L (Supplementary Fig. 9E), and mouse TNF/IL1β-primed neutrophils exposed to sCD95L exhibited increased expression and cleavage of OTULIN (Supplementary Fig. 9F), confirming that the caspase-driven ROS production was conserved in mice. DB550 treatment dramatically improved the kidney architecture of anti-GBM treated mice (Supplementary Fig. 9G and S9H) and restored kidney function in terms of blood filtration, as monitored by the concentrations of blood creatinine and urea (Supplementary Fig. 9I). In association with the improvement in clinical scores, we observed that DB550 treatment significantly reduced the trafficking and accumulation of neutrophils in the kidneys (Supplementary Fig. 9J).

Overall, these findings demonstrated that the CD95-mediated non-apoptotic signal exacerbates clinical symptoms in AAV mice and highlight that inhibition of this signal represents an attractive therapeutic option to prevent kidney failure in patients with AAV.

## Discussion

AAV neutrophil-mediated ROS production contributes to blood vessel damage and organ failure by oxidizing a variety of targets, including proteins, lipids, and DNA^65^. This study reveals that the metalloprotease-cleaved CD95L concentration is increased in patients with AAV and is associated with disease severity. Moreover, AAV neutrophils exposed in vitro to sCD95L undergo an oxidative burst inhibited by DB550^27^, a peptidomimetic that prevents the CD95/PLCγ1 interaction and abrogates CD95-mediated Ca^2+^. Notably, inflammatory cytokines, such as IL1-β or TNF, can prime neutrophils to overrespond to sCD95L, suggesting that these cytokines constitute an amplificatory pathogenic loop.

Mechanistically, using in-depth proteomic analysis, we uncovered a novel CD95-mediated signaling pathway in which sCD95L triggers the caspase-10-mediated cleavage of OTULIN. OTULIN cleavage occurs after aspartic acid cleavage at positions D31 and D54, resulting in an increased LUBAC activity, which, in turn, induces mitochondrial ROS overproduction in AAV neutrophils. The PUB-interacting motif (PIM) of OTULIN encompasses the ED^54^MYR sequence (amino acids 53 to 57) (Fig. 4F), which is similar to the PIM motif in p97 (*i.e*., sequence DDLYG), interacting with the PUB domain of peptide N-glycanase (PNGase)^66^. Because the PIM domain of OTULIN is crucial for HOIP interaction and the regulation of LUBAC activity^55^, caspase-10 cleavage at D54 represents a novel mechanism to stimulate LUBAC activity by preventing OTULIN/HOIP interaction.

The strong interplay between caspase-8 activation and the downstream recruitment and activation of caspase-10 complicates the identification of the biological role of each initiator caspase *in cellulo*. However, using a cell-free assay, we established that caspase-10 was responsible for OTULIN cleavage at D54 and D31. Consistently, ROS production is impaired in immortalized T-lymphocytes from patients with ALPS-CASP10^59^, confirming the pivotal role played by caspase-10 in CD95-mediated non-apoptotic signaling. However, how sCD95L mainly activates the caspase-10 pathway at the expense of apoptotic caspase-8-dependent signaling remains to be elucidated. Whether single and double caspase-10-driven cleavage of OTULIN has different effects on the biological function of DUB also remains an open question. Nonetheless, our data may explain earlier observations showing that caspase-10 activation stimulates NF-κB signal^19^, as LUBAC activation stimulates NF-κB through linear ubiquitination of NF-κB Essential Modulator (NEMO)^67^. Notably, caspases -3 and -7 can also cleave OTULIN at a position close to D31. These findings are consistent with a recent study showing that caspases-3 is involved in OTULIN cleavage at aspartic acid 31 generating an inhibitor of the apoptotic signaling induced by either the RIG-I pathway or TNF in the presence of cycloheximide^51,68^.

Although caspase-10 is not expressed in rodents, our data reveal that caspase-8 and LUBAC activities are responsible for sCD95L-mediated ROS production in mouse neutrophils, suggesting that caspase-8, the caspase-10 ortholog in mice^69^, may substitute for its function in CD95-stimulated mouse neutrophils.

The upregulation of OTULIN observed in most AAV neutrophils, compared with healthy donor cells, requires further investigation to determine whether this upregulation represents a negative feedback loop aimed at counteracting LUBAC overactivation or whether increased otulin expression followed by caspase-10-driven cleavage instead contributes to LUBAC overactivation and CD95-mediated ROS overproduction in AAV neutrophils. Biallelic loss-of-function (LoF) mutations in OTULIN cause otulipenia, also known as OTULIN-related autoinflammatory syndrome (ORAS)^70,71^, a pathology characterized by neutrophilic dermatitis, panniculitis, lymphadenopathy, and gastrointestinal inflammation. From a clinical perspective, this study suggests a possible pathophysiological continuum between ORAS and AAV. In addition, the ability of caspase-10 to cleave OTULIN and thereby release the LUBAC activity raises broader questions regarding its role in inflammatory and autoimmune disorders. For instance, caspase-10 activity inhibition has been described in many cancers^72–74^. Because LUBAC activation is pivotal for mounting an efficient immune response to self (damage-associated molecular patterns or DAMPs) and pathogen (pathogen-associated molecular patterns or PAMPs) danger signals^31^, one can envision that, in contrast to inflammatory disorders, abrogation of caspase-10-mediated OTULIN cleavage in transformed cells may restrain LUBAC activity and represent a molecular mechanism limiting pro-inflammatory and anti-tumor responses.

Although this study shows that caspase-10 cleaves OTULIN at D54 in neutrophils exposed to sCD95L, the cleavage appears weak, as the 35 kDa fragment is faint compared with the uncleaved and 37 kDa forms (Fig. 5A, B). Notably, OTULIN regulates multiple cellular signaling pathways, including Met1-linked ubiquitination of TNFR1, Toll-like receptors (TLRs), and nucleotide-binding oligomerization domain-containing protein 2 (NOD2). In addition, this deubiquitinase can modulate mTOR signaling and endosome-to-plasma membrane trafficking^75^. Therefore, CD95-driven OTULIN cleavage may target only a limited pool of the enzyme, which could explain the relatively low level of cleavage observed in neutrophils.

In an AAV mouse model, neutrophils bind to anti-MPO antibodies and undergo modification of their adhesion molecules, promoting their adhesion to the glomerular endothelium^76^. Because membrane-bound CD95L expressed by endothelial cells promotes the adhesion and endothelial transmigration of neutrophils^77^, we hypothesize that mCD95L and sCD95L may act together to exacerbate AAV pathology. Specifically, mCD95L on the surface of endothelial cells in inflamed kidneys facilitates neutrophil rolling, adhesion, and extravasation, whereas its MMP-cleaved counterpart stimulates ROS production, increasing vessel permeability and damage. The accumulation of sCD95L in the sera of patients with AAV raises the question of whether metalloproteases and/or ADAMs are responsible for CD95L cleavage at the plasma membrane of endothelial cells in inflamed organs. In conclusion, this study highlights the pivotal role of sCD95L in the pathophysiology of AAV, particularly its impact on neutrophils, and identifies a potent therapeutic strategy that warrants clinical evaluation. Although many AAV-like mouse models exist, they do not recapitulate all features of human AAV^6^. Most models were chosen because they develop pauci-immune focal segmental crescentic glomerulonephritis^6^. In this study, we generated three ANCA vasculitis-like mouse models to strengthen our conclusions: sCD95L acts as a cytokine contributing to disease severity, and DB550 represents an attractive therapeutic candidate.

Although DB550, a selective inhibitor of the CD95-mediated non-apoptotic signal^27^, alleviates clinical symptoms in ANCA-like mouse models, we did not selectively eliminate CD95 in neutrophils. Future studies could overcome this limitation by using Ly6G-CRE mice crossed with their CD95-floxed. For some of the mechanistic studies, we employed the neutrophil-like HL-60 cell line because primary neutrophils are non-dividing cells and have a short half-life, making genetic manipulations challenging. While this cell line reproduces key aspects of CD95-mediated pro-inflammatory signaling in primary cells (Supplementary Fig. S4), the use of HL60 represents a potential limitation.

Of note, it has been reported that neutrophils from patients with AAV produce less ROS than those from healthy donors^78–80^. However, these studies focused on the phagocytic process, and the reduced ROS production was attributed to defects in NADPH oxidase–driven ROS generation^80,81^. We therefore propose that this apparent discrepancy may reflect different mechanisms of ROS production: a down-regulated NADPH oxidase–dependent ROS generation contributing to phagocytosis defects in patients with AAV *versus* an upregulated sCD95L-induced ROS production which is mitochondria dependent and implicated in the pathology severity. Finally, although we have identified a caspase-10-dependent mechanism contributing to ROS generation, the precise molecular interplay between this caspase-driven LUBAC activation and Ca²⁺ signaling remains to be fully elucidated.

## Methods

### Study participants

Fresh healthy adult donor blood samples were obtained from a blood bank (“Etablissement Français du Sang Nouvelle-Aquitaine”; EFS-NAQ, Bordeaux, France). The EFS-NAQ displays an agreement with the French Ministry of Health and Research (agreement number AC 2018-3149) to collect additional blood volume from healthy donors who do not oppose the use of these samples for research purposes, and signed an informed consent. Patients with ANCA-associated vasculitis were recruited from the rheumatology, nephrology, and internal medicine departments of Bordeaux University Hospital. The patients were diagnosed with vasculitis using the 2012 Revised International Chapel Hill Consensus Conference Nomenclature criteria^34^. The retrospective serum collection came from the Bordeaux University Hospital biobank. To enroll additional patients, an interventional research protocol named VASC-FAS conforming to French and European Ethics standards was reviewed and approved by an independent ethics committee (Bordeaux cohort, ethical approval authorization number: 18/07/23/39007). The VASC-FAS research protocol was registered at ClinicalTrials.gov (number: NCT03698071). To confirm the results obtained in the Bordeaux cohort, sCD95L was assessed in a longitudinal cohort of patients with AAV before and after immunosuppressive treatment (at 0 and 6 months after treatment) from the Angers University Hospital (Cohort Maine-Anjou, Ethical authorization number 2018-MR03-02, Personal Protection Committee CB 2015/09). In the two patient cohorts, AAV activity was assessed using the 3.0 version of the Birmingham Vasculitis Activity Index (BVAS 3.0), which is the most commonly used AAV activity score^82^. Control skin biopsies were obtained via plastic breast reduction surgery. Control kidneys were obtained from nephrectomies performed for small local adenocarcinomas, and slides were removed from the tumor site.

### Reagents and antibodies

The selective CD95 inhibitor DB550 was synthesized as previously described^27^. Caspase inhibitors including zIETD-fmk (caspase-8 inhibitor; #FMK-007), zAEVD-fmk (Caspase-10 inhibitor; #FMK-009), and the pan-caspase inhibitor zVAD-fmk (#FMK001) were obtained from Biotechne (Tocris Bioscience, Bristol, UK). The caspase-3 inhibitor, zDEVD-fmk, was purchased from Euromedex (#TA-T6005; TargetMol, Boston, MA, USA). Coelenterazine-h was purchased from Thermo Fisher Scientific (#C6780, Waltham, Massachusetts, USA). Human soluble CD95L (#310-03H), human TNF (#300-01 A), human IL1β (♯200-01B), murine IL-1β (#211-11B) and human C5a (#300-70B) were from Peprotech (Thermofisher, Waltham, MA, USA). Human IL-6 (#130-093-931), viability 450/520 Fixable dye (#130-130-404), and anti-mouse CD11b VioBright FITC (clone REA592, #130-113-243) were purchased from Miltenyi Biotec (Bergisch-Gladbach, Germany). Anti-caspase-10 polyclonal antibody (#ab2012) and anti-CD31 (clone EPR17259, #AB182981) were purchased from Abcam (Cambridge, UK). Monoclonal anti-FLAG M2 (clone M2, #F3165), anti-Linear Ubiquitin (clone 1E3 ZooMAb, #ZRB2114) and anti-β-actin (clone AC-15, #A5441), were from sigma-Aldrich (Sigma Aldrich Chimie, Saint-Quentin-Fallavier, France). V5-Tag antibody (clone SV5-Pk1, #MCA1360) was purchased from Biorad (Marnes-la-Coquette, France). Anti-CD95L (G247-4, #556387) and anti-FADD (clone A66-2, #556402) antibodies were purchased from BD Biosciences (San Jose, CA, USA). Anti-caspase-8 mAb (clone 1C12, #9746), polyclonal anti-OTULIN antibodies (#14127), β-actin mAb (#8457) and monoclonal anti-caspase 3 mAb (clone D3R6Y, #14220), were purchased from Cell signaling (Beverly, MA, USA). Alexa Fluor 488-conjugated anti-human CD95 (clone DX2, #305616) was purchased from BioLegend (San Diego, CA, USA). Recombinant and active caspase-3 (#707-C3/CF), caspase-7 (#823-C7/CF), caspase-8 (#705-C8/CF) and caspase-10 (#834-CP) were purchased from R&D Systems (Minneapolis, MN, USA). Anti-rabbit IgG-HRP (#4090-05), anti-mouse IgG2a human ads-HRP (#1080-05), anti-mouse IgG1human ads-HRP (#1070-05) were purchased from Southern biotech (Birmingham, AL, USA). Anti-CD31 (#AB182981, Abcam), Mouse On Mouse (MOM) Blocking Reagent (#MKB-2213-1) was purchased from Vectorlabs (Newark, Californie, USA). Rat anti-mouse CD16/CD32 Fc Block (Clone 2.4G2, #553142) was from BD Bioscience (Franklin Lakes, NJ, USA). A full list of reagents and dilutions is provided in Supplementary Table 4.

### Cells and transfection

Human neutrophils were isolated from healthy donors or patients with AAV through negative selection by incubating whole blood with a cocktail of magnetically labeled antibodies targeting non-target cells (#130-104-434, Miltenyi Biotec). The cells were removed by immunomagnetic depletion using a MACSxpress Separator (Miltenyi Biotec). The remaining cells were recovered, and erythrocytes were eliminated using a MACSxpress Erythrocyte Depletion Kit (#130-098-196, Miltenyi Biotec). Neutrophil-enriched supernatant was collected, centrifuged and resuspended in RPMI complemented with 10% fetal calf serum (FCS), 2 mmol/L L-glutamine and penicillin-streptomycin.

Murine neutrophils were isolated by flushing the bone marrow from the femur and tibia. The erythrocytes were eliminated using ACK Buffer (#A1049201, Thermo Fisher Scientific, Waltham, MA, USA). Living neutrophils (SSC^high^/ Viability-dye^low^/ CD11b^high^) were sorted using a BD FACS Melody Cell Sorter (BD Biosciences). Cells were resuspended in RPMI supplemented with 10% FCS, 2 mM L-glutamine, and penicillin-streptomycin.

HEK/293 T and HL60 cells were purchased from ATCC. HEK/293 T cells were cultured in DMEM supplemented with 10% heat-inactivated FCS, HL60 cells and ko counterparts were cultured in IMDM 10% FCS and 2 mmol/L L-glutamine at 37 °C 5% CO_2_. HEK-293 cells were counted and seeded (1 × 10^6^ cells) in 10 cm dishes in 10 mL DMEM medium complemented with 10% FCS and 2 mmol/L L-glutamine. After 24 h, cells were transfected using 3 μg of cDNA (Ratio 2:1 for OTULIN/caspase-8-encoding vectors) using the calcium/phosphate protocol. The pan-caspase inhibitor zVAD-fmk was added after transfection at a concentration of 10 μM. 24 h post-transfection, cells were collected, washed twice with phosphate-buffered saline (PBS), and lysed using radioimmunoprecipitation assay (RIPA; #sc-24948A, Santa Cruz Biotechnology, Heidelberg, Germany) buffer.

### Vectors

Human OTULIN sequences (WT, D54E (codon GAA), and Y56F (codon TTC) were fused in frame with a FLAG tag (DYKDDDDK) and inserted into the pLVX-IRES-TdTomato vector (TaKaRa, Kusatsu, Japan) using XhoI/BamHI restriction sites. Similarly, the truncated Delta31 and Delta54 OTULIN constructs started at amino acids 32 (glycine) and 55 (methionine), respectively, and were fused in-frame with a FLAG tag. A methionine codon (ATG) was inserted prior to ^32^. G and ^55^. M for the Delta31 and Delta54 OTULIN constructs, respectively, and the sequences were inserted into the pLVX-IRES-TdTomato vector using the XhoI/BamHI restriction sites. For each construct, a Kozac sequence was inserted between the XhoI site and the start codon ATG.

For the HOIP, HOIL-1, and Sharpin constructs, a methionine codon (ATG) was inserted prior to the HA tag (GKPIPNPLLGLDST) and coding sequences of HOIP (NM_017999), HOIL-1 (NM_006462), and Sharpin (NM_030974). These inserts were inserted into pcDNA3.1(+) using the BamHI/XbaI cloning sites.

Human C360A (cysteine at position 360 replaced by an alanine-codon TGC replaced by GCC) and wild-type caspase-8 (NM_033355.4) were inserted into pLVX-IRES-tdTomato using XbaI/BamhI. Human OTULIN, caspase-8 (C360A), wild-type, and C358A caspase-10 (NM_032977.4) sequences were fused to a linker (GGGGS)_2_, either the F2 or F1 fragments from Renilla luciferase as described here^27,52^ and a stop codon (TAA). These constructs were inserted into pcDNA3.1(+) using the XbaI/BamhI sites. A FLAG-tag (DYKDDDDK) was inserted at the N-terminus of the caspase-10 constructs. The pLenti-Flag-WT-OTULIN and pLenti-Flag-D31E OTULIN constructs were kindly provided by Dr. Maya Saleh (University of Bordeaux, France) and are described in this manuscript^51^.

### Soluble CD95L and IgCD95L production

HEK/293 T cells maintained in an 10% FCS-containing medium were transfected using the calcium/phosphate precipitation method with 3 μg of empty plasmid (control pLVX-IRES-tdTomato vector), wild-type human CD95L (full-length CD95L was cleaved by metalloproteases to generate sCD95L), or IgCD95L^43^-containing pLVX-IRES-tdTomato vectors. Twenty-four hours after transfection, the medium was replaced with Opti-MEM (Thermo Fisher Scientific). After 5–7 days, the media containing metalloprotease-cleaved CD95L (sCD95L) or control (pLVX-IRES-tdTomato) were harvested. Dead cells and debris were eliminated through a first step of centrifugation (4000 rpm for 10 min) followed by a second step, for the elimination of exosomes by ultracentrifugation at 100,000 × *g* for 1 h. The control medium (pLVX-IRES-tdTomato vector-transfected cells) served as the untreated cells. The different media were dialyzed overnight against PBS at 4 °C using membrane tubing (Spectra/por, standard RC tubing, MW 6-8 kDa, # 132660, ThermoFisher scientific) and next concentrated using Amicon Ultra Centrifugal Filter, 10 kDa MWCO (Sigma). To quantify the concentration of sCD95L, we used an anti-human CD95L ELISA kit (#ab45892), following the manufacturer’s instructions (Abcam).

### Generation of k.o. HL60 cells

Mixed guide RNAs targeting exon 5 of caspase-3 (AUUGUGGAAUUGAUGCGUGA; AGUUUCUGAAUGUUUCCCUG; UGUUCCAAGGAAUGACAUCU), exon 2 of caspase-8 (CAGAAAUCUUUAUGAUAUUG; GCGGAAUGUAGUCCAGGCUC ; CCUCCAACAUUCUCUUUUCC), exon 1 of OTULIN (CGCGGACUCACUGCUCGGCC; CGCGAGCGACCGCAUGAGUC; CGGCCACGGCGCGGGACGGC) and exon 2 of caspase-10 (UGAAAUCUCAAGGUCAACAU; CUGAUUAUUGAUUCAAACCU; AUUGGUCCCCAACAAGAAGC) were used to generate knockout pools of HL60 cells using CRISPR/Cas9 (Synthego, Redwood City, CA, USA). For each cell population, ko efficiency was tested by western blotting, and these cell populations served for the different experiments to avoid clonal effects.

### Caspase assay

HEK/293T cells (1 × 10^6^) were transfected using the calcium/phosphate method using OTULIN constructs, and 24 h after transfection, the cells were lysed using 1 mL of RIPA buffer. After elimination of the debris by centrifugation (15 000 rpm/10 min), 100 μL of anti-FLAG mAb-coated magnetic beads (clone M2, #8823, Sigma) was added to the clarified lysate and rotated 1 h at room temperature (RT). Beads were washed five times with TBS (50 mM Tris, 160 mM NaCl, pH 7.5) and an additional wash was performed using caspase buffer (10 mM HEPES (pH 7.2), 5 % glycerol, 100 mM NaCl, 0.1% CHAPS hydrate (w/v), 1 mM EDTA, 10 mM DTT). The beads were resuspended in 100 μL caspase buffer. 20 μL of the washed beads were mixed with 1 μL of activated caspase-8 (705-C8/CF), -10(834-CP), -3(707-C3/CF) or -7 (823-C7/CF) and incubated for 90 min at 37 °C in a shaker (300 rpm). The reaction was terminated using 5 μL of loading buffer (200 mM Tris-HCl, 8% (w/v) SDS, 6 mM bromophenol blue, 30% Glycerol) complemented with 5% β-mercaptoethanol, heated for 5 min at 95 °C and loaded in sodium dodecyl sulfate-polyacrylamide gel electrophoresis (SDS-PAGE) and an anti-OTULIN immunoblot was performed.

### Mice experiments

Female C57BL/6 J mice (6–8 weeks were purchased from Charles River Laboratories (Les Oncins, France) were housed in a pathogen-free environment (Bordeaux University, approval n° B33-063-916 (Biosafety level 2 facility) with access to *ad libitum* supply of standard chow and tap water. They were kept under a standard cycle of 12 h day and night and in a controlled temperature between 18 to 23 °C and humidity from 40 to 60%. The mice were randomized into different groups before treatment and bred separately. The treatment protocol was reviewed and approved by the Institutional Animal Ethics Committee (authorization numbers: APAFIS #19506 and #48633). Although no major sex difference exists in the occurrence of AAV^83^, we used female mice since the two well-characterized mouse models of severe ANCA-induced vasculitis in lungs^63^ and kidneys^61^ have been originally developed using only females (Female mice at 8–12 wk of age).

To induce glomerulonephritis (GN), mice were intravenously injected (retro orbital sinus injection) with 100 µL of a sheep anti-GBM nephrotoxic serum (PTX-001S, Probetex Inc., San Antonio, USA) for 3 days in a row. The control group received 100 µL of sheep non-immune serum (PTX-000S, Probetex Inc.) for 3 days. At day 0, the mice were injected intraperitoneally (i.p.) with 0.5 mg/kg of lipopolysaccharide (LPS, *Escherichia coli* O55:B5, #L6529, Sigma Aldrich). Mice (*n* = 5 per group) were treated intraperitoneally with DB550 (10 mg/kg) or vehicle (10% ethanol in water) for 3 days a week. The DB550 and control treatments were applied 24 h after GN induction. All mice were euthanized at day 14 by controlled CO_2_ inhalation, and their urine, peripheral blood, and kidneys were collected.

To induce an AAV-like kidney model, mice were intravenously injected with 100 µL of sheep anti-GBM nephrotoxic serum at day 0. At day 5, mice were i.p. administered with 0.75 mg of each murine monoclonal anti-myeloperoxidase (anti-MPO) antibody clones 6D1 and 6G4 (#BX-BE0393-50MG and #BX-BE0392-50MG, Euromedex) and 1 h later, with 150 µL of LPS (0.5 mg/kg). As a control for anti-GBM, mice were intravenously administered with 100 µL of sheep non-immune serum at day 0. As a control for anti-MPO, mice were i.p. administered with 0.75 mg of each isotypes IgG2a and IgG2b (#BE0086-50MG and #BE0085-50MG, Euromedex) at day 5. As control for LPS, 1 h after isotype control injections, mice received 150 µL PBS in i.p. Mice (*n* = 5 per group) and were treated i.p. with DB550 (10 mg/kg) or vehicle (10% ethanol in water) at days 4, 6, 8, 10, and 11. All mice were euthanized by controlled CO_2_ inhalation at day 12, and their urine, peripheral blood, and kidneys were collected.

To induce an AAPV-like model, mice were intratracheally (i.t.) instilled with 50 μl of PBS containing 10 μg LPS (Serotype 026:B6; #L8274, Sigma-Aldrich) plus 10 μg fMLP (#59880-97-6, Sigma-Aldrich), followed by i.p. injection of 1 mg anti-MPO (clones 6D1 and 6G4, 0.5 mg of each clone) in 200 μl PBS. Control groups were treated by PBS and isotypes. Mice (*n* = 3-5 per group) were treated i.p. with DB550 (10 mg/kg) or vehicle (10% ethanol in water) at days 1, 2, 3. All mice were euthanized by controlled CO_2_ inhalation on day 4. Weight loss was monitored daily.

For these 3 models, kidneys or lungs were snap frozen and stored at -80 °C. A portion of the organs was fixed overnight in 4% paraformaldehyde (PFA) for histological and immunofluorescence. Serum creatinine and urea levels were quantified using the CRI (Plateforme de Biochimie UMR 1149; Université Paris Diderot, France). Histopathological analyses were realized in a blinded manner.

For the AAPV-like model, Broncho-Alveolar Lavages (BAL) were harvested using two washes of PBS (500 µL)*.* Cell counting was performed by counting an average of 250 cells using cytospin (Shandon CytoSpin 3, ThermoFisher Scientific, Illkirch, France) after May-Grünwald-Giemsa staining (Diff Quick, Medion Diagnostics, Düdingen, Switzerland) according to the manufacturer’s instructions.

### Human immunohistopathology

Analysis of patients with AAV kidneys and skins was performed using 4-µm-thick whole-slide sections, obtained with a microtome from FFPE tissue blocks transferred onto plus charged slides, followed by Discovery Fluorescence IHC multiplex. Staining was performed using anti-CD31 (#Ab28364, Abcam) and anti-human CD95L (clone G247-4, #556387, BD Pharmingen, Franklin Lakes, NJ, USA) antibodies. Expressions were visualized by Rhodamine for CD95L and FAM for CD31, and sections were counterstained with DAPI and cover-slipped using fluoromount (#ADI-950-260-0025, Enzo Life Sciences, Farmingdale, NY, USA). Whole-slide scans of the fluorescent-labeled sections were obtained at magnification X20 using a Pannoramic Confocal digital slide scanner (3D-Histech Ltd., HU). For neutrophil quantification in paraffin-embedded kidneys, cells were stained with an elastase antibody (#NBP2-57576, Novus Biologicals, Centennial, CO, USA), and quantification was performed by image analysis using NIS element software (Nikon, Japan). The threshold was applied to the hue-saturation-intensity (HSI) to discriminate between hematoxylin counterstaining (blue) and 3,3’-Diaminobenzidine (DAB) immunostaining (brown, elastase-positive neutrophils), allowing the assessment of the number of cells per mm^2^.

### Mouse immunohistopathology

For morphological studies in mice, kidney and lung sections from paraffin-embedded tissues (2.5 μm) were stained with a Masson trichrome or a hematoxylin/eosin protocol, respectively and analyzed by light microscopy. The sections were read by three operators using an NDP Viewer (Hamamatsu, Japan). In one kidney section, 60–80 glomeruli were counted and assessed in a blinded manner to determine glomerular crescent formation. Cellular glomerular crescents are defined as two or more layers of proliferating cells in Bowman’s space and can be circumferential (more than 1/3 of the glomerulus) or segmental.

For the kidney, immunofluorescent staining was performed using anti-CD31 (#AB182981, Abcam) and anti-human CD95L antibodies (clone G247-4). Tissues were blocked using M.O.M Blocking Reagent following manufacturer instructions, purified rat anti-mouse CD16/CD32 Fc Block (Clone 2.4G2, #553142, BD Bioscience) and 10% SVF. CD31 was detected by incubating tissues with HRP-conjugated goat anti-rabbit IgG (#MP-7451, Eurobio Scientific) and TSA #Fluorescein reagent pack (#SAT701001EA, Quanterix, Billerica, MA, USA), and CD95L was detected with an Alexa Fluor 633-congated goat anti-Mouse IgG (H + L) secondary antibody(#A21052, ThermoFisher). The slides were scanned using Evident VS200 and analyzed using QuPath software.

### Spleen handling and flow cytometry

Spleen were harvest in PBS and dissociated by mechanical disruption on a 70 µm cell strainer. Splenocytes were incubated for 5 min in ACK lysis buffer (150 mM of NH4Cl, 10 mM of KHCO3, 1 mM of EDTA, pH 7) and neutralized with twice the volume of PBS. Staining was performed on 5.106 cells, and dead cells stained with Viability 405/520 Fixable Dye (#130-130-404, Miltenyi Biotec) in PBS. Non-specific Fc binding site were saturated with 10 µg/ml of anti-CD16/CD32 antibody (2.4G2) (#BE0224, BioXcell). Cells were incubated with following fluorophores conjugated antibody (clone): FITC Ly6G (1A8), APC CD3 (17A2), Alexa Fluor 700 CD45 (30-F11), APC-Cyanine7 CD11b (M1/70), Pacific Blue I-Ab (AF6-120.1), Brilliant Violet 785 CD19 (6D5), PE CD11c (N418), PE-Dazzled 594 Ly6C (HK1.4), Alexa Fluor 700 CD23 (B3B4), PE-Cyanine7 CD21/35 (7E9), FITC CD4 (RM4-5), PerCP-Cy5.5 CD44 (IM7), Brilliant Violet 421 CD3 (17A2), Brilliant Violet 605 CD8 (53-6.7), PE-Cyanine7 CD62L (MEL-14) at an optimized concentration. For intranuclear staining, Foxp3 / Transcription Factor Staining Buffer Set (eBioscience, ThermoFisher) were used following manufacturer protocol, and staining was performed using the following fluorophores conjugated antibody (clone) Brilliant Violet 785 RoRγT (Q31-378), PE FoxP3 (FJK-16s) at an optimized concentration, and gating were performed using fluorescence minus one (containing the control isotype)

### Flow cytometry

Isolated PNN were stained with viability marker (Fixable Viability Dye eFluor780 (#65-0865-14, eBiosciences, Santa Clara, CA, USA), anti-CD95 Pacific Blue (clone DX2, #305619 BioLegend), or control isotype (IgG1k-Pacific Blue, #400151, BioLegend) in 5% BSA-PBS for 15 min (dark, room temperature) before washing and flow cytometry (Canto II, BD Biosciences) analysis.

To select CD95-k.o. HL60 clones, we performed a limiting dilution experiment and the next clones were labeled with an Alexa Fluor 488-conjugated anti-human CD95 (clone DX2, #305616, Biolegend) for 30 min at 4 °C before washing with 5% BSA-PBS twice and analysis by flow cytometry (CytoFLEX, Beckman Colter, Brea, CA, USA).

### DMSO-treated HL60

HL60 cells were cultured in IMDM medium supplemented with 10% FBS in a humidified atmosphere containing 5% CO_2_ at 37 °C. HL60 cells were differentiated into neutrophils by incubation cells for 72 h with 1.25% of DMSO. Neutrophil-like HL60 cells were then treated with recombinant human sCD95L (100 ng/mL) at the indicated time points to measure calcium response and ROS production. Cells were lysed, and immunoblotting was performed.

### Renilla luciferase-based protein-fragment complementation assay

HEK/293 T cells were transfected by the calcium/phosphate method using 3 μg for each F1- and F2-encoding pcDNA3.1(+) vectors. Transfected cells were cultured for 24 h prior to PCA analysis as previously described^25^. Briefly, transfected cells (10^6^) were washed and resuspended at 10^6^ cells/mL in PBS, and 100 μL was placed in a white flat-bottom 96-well plate. Coelenterazine-h (2 µg/mL, #C6780, Thermo Fisher Scientific) was added to each well, and Renilla luciferase activity was monitored for the first 30 s using FLUOstar omega (BMG Labtech, Champigny s/Marne, France).

### Cytosolic calcium

Cells were seeded on glass coverslips and mounted in an Attofluor cell chamber (#A7816, Thermo Fisher Scientific) positioned on the stage of an inverted epifluorescence microscope (Olympus, IX70) equipped with a × 40 UApo/340 1.15 W objective. Cells were loaded with Cal-520 (1 μM, #21130, AAT Bioquest, Pleasanton, CA, USA) at 37 °C (95% O_2_; 5% CO_2_) in Hanks’ balanced salt solution (HBSS, #14175053, Gibco, Thermofisher) supplemented with 2 mM CaCl_2_ and 2 mM MgCl_2_ for 30 min. Cal-520 exhibits limited compartmentalization in intracellular stores and is relatively resistant to leakage compared with other classic Ca^2+^ dyes. The cells were rinsed with HBSS and incubated in the absence of the Ca^2+^ probe for 15 min to complete the dye de-esterification. Cal-520 was excited at 485 ± 22 nm, and images were captured at 530 ± 30 nm at constant 10 s intervals, at 12-bit resolution, by a fast-scan camera Photometrics, Huntington Beach, CA, USA). All images were background-subtracted. Regions of interest corresponding to the recorded cells were drawn to analyze the fluorescence signal. Imaging was controlled using Universal Imaging software, including MetaFluor and MetaMorph. Fluorescence intensity changes were normalized to the initial fluorescence value *F*_0,_ expressed as *F*/*F*_0_ (relative [Ca^2+^]_cyt_) and summarized as mean +/− S.D. Each experiment was independently repeated thrice, and for each experimental condition, we displayed the mean ± SD of 41 single-cell traces. Calcium traces were quantified by determining the curve (AUC) using the OriginPro 7.5 software (OriginLab).

CD95 activation and intracellular Ca^2+^ signaling were assessed using recombinant human sCD95L (100 ng/mL, #310-03H; Peprotech). In some experiments, cells were placed in a Ca^2+^-free medium consisting of HBSS, in which CaCl_2_ was omitted and 100 µM EGTA was added to chelate residual Ca^2+^ ions. This medium was added to the cells immediately before recording to avoid leakage of intracellular calcium stores. To evaluate the role of inflammatory cytokines in the CD95-mediated Ca^2+^ signal and ROS production, cells were pre-treated with TNF (5 ng/mL), IL-1β (10 ng/mL), C5a (10 ng/mL), or IL-6 (10 ng/mL) for 30 min before experiments. To appreciate the ubiquitin ligase activity of LUBAC, cells were treated with LUBAC inhibitor HOIPIN-1 (10 µM, #JTP-0819958 MedChemExpress, Monmouth Junction, NJ, USA).

### Mitochondrial calcium

Single-cell mitochondrial calcium imaging was performed using Rhod2-AM- and MitoTracker-loaded cells. Experiments were conducted using the set-up described above, except that a Cy3 set filters (excitation: 535 ± 20 nm, emission: 605 ± 30 nm) was used for Rhod2 and a GFP set filters (excitation: 485 ± 22 nm, emission: 530 ± 30 nm) for mitotracker green.

The cells were loaded with 3 μM Rhod2-AM (#R1245MP, Thermo Fisher Invitrogen) and MitoTracker Green (#M46750, Thermo Fisher Invitrogen) at room temperature in HBSS for 45 min. In some experiments, cells were placed in a Ca^2+^-free medium (HBSS without CaCl_2_ and complemented with 100 μM EGTA). This medium was added to the cells immediately before recording to avoid leakage of intracellular calcium stores. To analyze the molecular mechanism responsible for mitochondrial Ca^2+^ loading, Bcl2 inhibitor ABT263 (100 nM, #CT-A263, Chimietek, Indianapolis, IN, USA) and Bcl2/VDAC1/3 interaction inhibitor L14-15 peptide (LAWTAGNSNTR) (10 µM, LifeTein, Somerset, NJ, USA) were pre-incubated 30 min before experiments.

### Cytosolic ROS

The H_2_O_2_ concentration was determined using H2DCFDA (#D399, Thermo Fisher Invitrogen) as a fluorescent probe. Oxidation of 2’, 7’-dihydrodichlorofluorescein (H2DCF, non-fluorescent) by ROS converts the molecule to 2’, 7’ dichlorofluorescein (DCF), which is highly fluorescent. To avoid cell toxicity, the cells were loaded with a low concentration of H2DCFDA (0.1 μM) for 15 min at room temperature. These loading conditions allowed us to obtain a stable baseline and to detect changes in ROS production in individual cells upon stimulation. The experimental set-up was identical to that described above, except that a GFP set filters (excitation: 485 ± 22 nm, emission: 530 ± 30 nm) was used. To monitor intracellular Ca^2+^ role in cytosolic ROS production, cells were pre-incubated at least 30 min with MitoTEMPO (10 µM, #SML0737, Sigma-Aldrich).

### Mitochondrial ROS

Mitochondria-specific superoxide was visualized using fluorescence microscopy with the MitoSOX Red reagent (#M36008, ThermoFisher Invitrogen). The MitoSOX Red reagent is a cationic derivative of dihydroethidium that permeates living cells and selectively targets mitochondria. Once in the mitochondria, the MitoSOX Red reagent is oxidized by superoxide and exhibits red fluorescence. Cells were incubated with 1 μM MitoSOX Red reagent for 15 min at 37 °C.

The experimental set-up was identical to that described above, except that a Cy3 set filters (excitation: 535 ± 20 nm, emission: 605 ± 30 nm) was used. ROS production was quantified by measuring the area under the curve (AUC). To evaluate the role of calcium in the CD95-mediated mitochondrial ROS production, cells were pre-incubated at least 30 min with BAPTA-AM (10 µM, #A1076, Sigma-Aldrich). To determine whether the ubiquitin ligase activity of LUBAC was involved in ROS production, cells were treated with LUBAC inhibitor HOIPIN-1 (10 µM).

### Immunoprecipitation

HEK/293 T cells were co-transfected by the calcium phosphate method with V5-tagged HOIP and flag-tagged wild-type, ∆54 or ∆31-OTULIN constructs. Forty-eight hours after transfection, cells were lyzed in a non-ionic detergent (Triton X-100)-containing HEPES buffer (25 mM HEPES, 150 mM NaCl, 2 mM NaF, 1 mM NaVO_4_, 2 mM EGTA and 1% TritonX-100). After removing debris (15,000 rpm, 10 min), immunoprecipitation was performed by adding 30 µL anti-FLAG M2 (#M8823) or anti-V5 (#SAE0203) magnetic beads overnight at 4 °C with rotation. Beads were washed twice with PBS and proteins were eluted in 30 µL Laemmli sample buffer (#1610747), boiled at 95 °C for 5 min. Proteins were resolved by 12% SDS-PAGE, and immunoblotting were performed using anti-FLAG and anti-V5 antibodies.

### Immunoblotting

Western blotting of the HEK cell supernatant was performed as previously described^25^. For the characterization of IgCD95L and sCD95L expression, 30 μL of supernatants was loaded and resolved in a 12% SDS-PAGE. Proteins were transferred to polyvinylidene fluoride (PVDF) membranes using a Trans-blot Turbo RTA transfer kit (#1704272, Life Science Bio-Rad, Hercules, CA, USA). Non-specific binding sites were blocked by incubating membranes for 60 min with TBST-Milk (M) (50 mM Tris, 160 mM NaCl, 0.1% (v/v) Tween 20 and 5% (w/v) dried skimmed milk at pH 7.4) and later incubated overnight at 4 °C with the corresponding primary antibody (anti-CD95L clone G247-4, #556387, BD Bioscience). To visualize the bound primary antibody, the membranes were washed with TBST and incubated with an appropriate peroxidase-labeled secondary antibody (anti-mouse IgG1-HRP #1070-05 was from Southern Biotech, Birmingham, USA) for 30 min. Membranes were revealed by enhanced chemiluminescence using Immobilon Forte Western HRP Substrate (#WBLUF0500, Millipore, Burlington, MA, USA) on Bio-Rad ChemiDoc Touch Imaging System.

For the detection of CD95L in the kidney and lung, the organ was homogenized manually in 900 µL of T-Per protein extraction buffer (#78510, ThermoFisher Scientific) mixed with protease and phosphatase inhibitor (ThermoFisher Scientific). The extract was centrifuged 20 min at 10000 rpm and the supernatant was stored at − 20 °C before immunoblotting analysis. Protein concentrations were determined in lung tissue by using the DC protein Assay (#5000111, Bio-Rad). Lysates were boiled at 95 °C, 5 min in reducing SDS sample buffer. Proteins (60 µg) were resolved on 12% polyacrylamide gel. Proteins were immunoblotted to a 0.2 µm PVDF membrane (Bio-Rad) using a Trans-blot Turbo RTA transfer kit (#1704272, Life Science Bio-Rad, Hercules, CA, USA) for 30 min. Membranes were blocked for 2 h in TBST-M at room temperature. Then, membranes were incubated with primary antibodies, anti-CD95L in TBST-M, over nigh at 4 °C. For mouse β-actin, membranes were incubated with anti-β-actin in TBST for 1 h at room temperature. The membranes were then washed 3 times in TBST and incubated with the appropriate secondary antibody conjugated to horseradish peroxidase (anti-mouse IgG, #1706516, Bio-Rad, France) for 1 h at room temperature. Protein bands were visualized following exposure of the membrane to ECL (#1705061, Bio-Rad, France) substrate solution using the iBright Imaging System (Invitrogen).

For detection of OTULIN, Caspase-10 and Caspase-8 in human and mice primary neutrophils, after incubation with sCD95L, the neutrophils were incubated with Diisopropylfluorophosphatase (DFP, #D0879, Sigma Aldrich) in ice for 15 minutes, centrifuged at 1500 rpm for 5 minutes. Cells were then lyzed using a neutrophil lysis buffer (HEPES 20 mM, NaCl 150 mM, 0.1% of 100X Triton and H2O) complemented with a Protease Inhibitor Cocktail (1 mM ATP, 400 µM Leupeptin, 400 µM Pepstatin, 3 mM PMSF, 1 mM Orthovanadate, 1 mM EDTA, 1 mM EGTA) in ice for 15 min. The extract was centrifuged 10 min at 13000 rpm and the supernatant was stored at − 20 °C. Proteins were dosed using the BCA (Bicinchoninic Acid) Assay. For each condition, 50 μg of proteins were resolved in a 12% SDS-PAGE and transferred on PVDF membranes. Membranes were incubated in TBST + 5% Milk with either anti-Otulin (#CST74125S, Cell Signaling Technology Inc), anti-Caspase 10 (clone 63131, #MAB834, Biotechne), anti-Caspase 8 (clone 1C12, #9746S, Cell Signaling Technology Inc). Protein bands were visualized following exposure of the membrane to ECL (Bio-Rad, France) substrate solution using iBright Imaging System (Invitrogen).

### ELISA assays

Soluble human CD95L was dosed using ELISA (#ab45892, Abcam) following the manufacturer’s instructions. The quantity of murine soluble CD95L was assessed using 50 μL tested without dilution by ELISA (#ab270894, Abcam), following the manufacturer’s instructions.

Kidney and lung lysates were analyzed by ELISA for Lipocalin-2 (#DY1857, Mouse DuoSet, R&D Systems) according to the manufacturer’s instructions.

### Proteomic analysis

HL-60 pelleted cells (10^7^) were supplemented with 100 µL of lithium dodecylsulfate (LDS #NP0007, 1X reagent from ThermoFisher), heated for 5 min at 99 °C, and briefly centrifuged (16,000 × *g* for 1 min). A volume of 25 µL of supernatant containing the soluble proteins was loaded on a NuPAGE 4–12% Bis-Tris gel (ThermoFisher). The proteins were subjected to electrophoretic migration for 5 min, stained with Coomassie SimplyBlue SafeStain (#LC6060, Thermo Fisher Scientific) for 5 min, and washed overnight with water with gentle agitation. Proteins were subjected to proteolysis using trypsin Gold (#V5280, Promega) in the presence of ProteaseMax detergent (#V2071, Promega) as previously described^84^. The resulting peptides were analyzed using an Orbitrap Exploris 480 high-resolution tandem mass spectrometer (Thermo Electron) operated in data-independent acquisition mode, essentially as described^85^. Peptides were identified with DIA-NN 1.8 software^86^ using a predicted spectral library built from the Swissprot Human database (downloaded 2022/04/04) with a maximum of two missed cleavages and one variable modification.

### TMRM apoptotic assay

DMSO-differentiated HL60 cells ($2\times {10}^{6}$ cells/mL) were incubated with TMRM reagent at a final concentration of 400 nM for 30 minutes at 37 °C in a humidified CO₂ incubator. Following staining, control medium, IgCD95L (100 ng/mL) or sCD95L (100 ng/mL) was added, and cells were incubated for 24 h. Cells were then transferred to FACS tubes for flow cytometry analysis.

### Statistical analyses

Statistical tests were performed using GraphPad Prism (unpaired and two-tailed Mann–Whitney or Student’s *t* test). Statistical tests are described in the respective figure legends.

### Reporting summary

Further information on research design is available in the Nature Portfolio Reporting Summary linked to this article.

## Supplementary information

Supplementary Information file

Transparent Peer Review file

Reporting Summary

Description of Additional Supplementary Files

Supplementary Data 1

Supplementary Data 2

## Source data

Source data

## Data Availability

Mass spectrometry proteomics data were deposited in the ProteomeXchange Consortium via the PRIDE partner repository with dataset identifiers PXD053681. Furthermore, the interpreted proteomic results are available in Figshare with the doi:10.6084/m9.figshare.26300509 [https://figshare.com/articles/dataset/Table_3_xlsx/26300509?file=47675464]. All other data are included in the Supplementary Information or available from the authors, as are unique reagents used in this Article. The raw numbers for charts and graphs are available in the Source Data file whenever possible. Source data are provided in this paper.
