# Supplementary material for: Soluble CD95L triggers Caspase-10-driven reactive oxygen species production in neutrophils and aggravates anti-neutrophil cytoplasmic antibody-vasculitis
Source: Nat Commun. 2026 Jun 20;17:7759. doi: 10.1038/s41467-026-74452-8 (PMC13434393; doi:10.1038/s41467-026-74452-8)
Supplement: Supplementary file 1 [file 41467_2026_74452_MOESM1_ESM.pdf]

SUPPLEMENTARY INFORMATION FILE

Soluble CD95L triggers Caspase-10-driven reactive oxygen species production in neutrophils and aggravates anti-neutrophil cytoplasmic antibody-vasculitis

Andréa Boizard-Moracchini[1,2,*], Dhouha Msalbi[3,*], Sarah Huot-Marchand[1, 4*], …, Patrick Legembre[3,16,‡,¶] and Patrick Blanco[1,2, 4, ‡,¶]

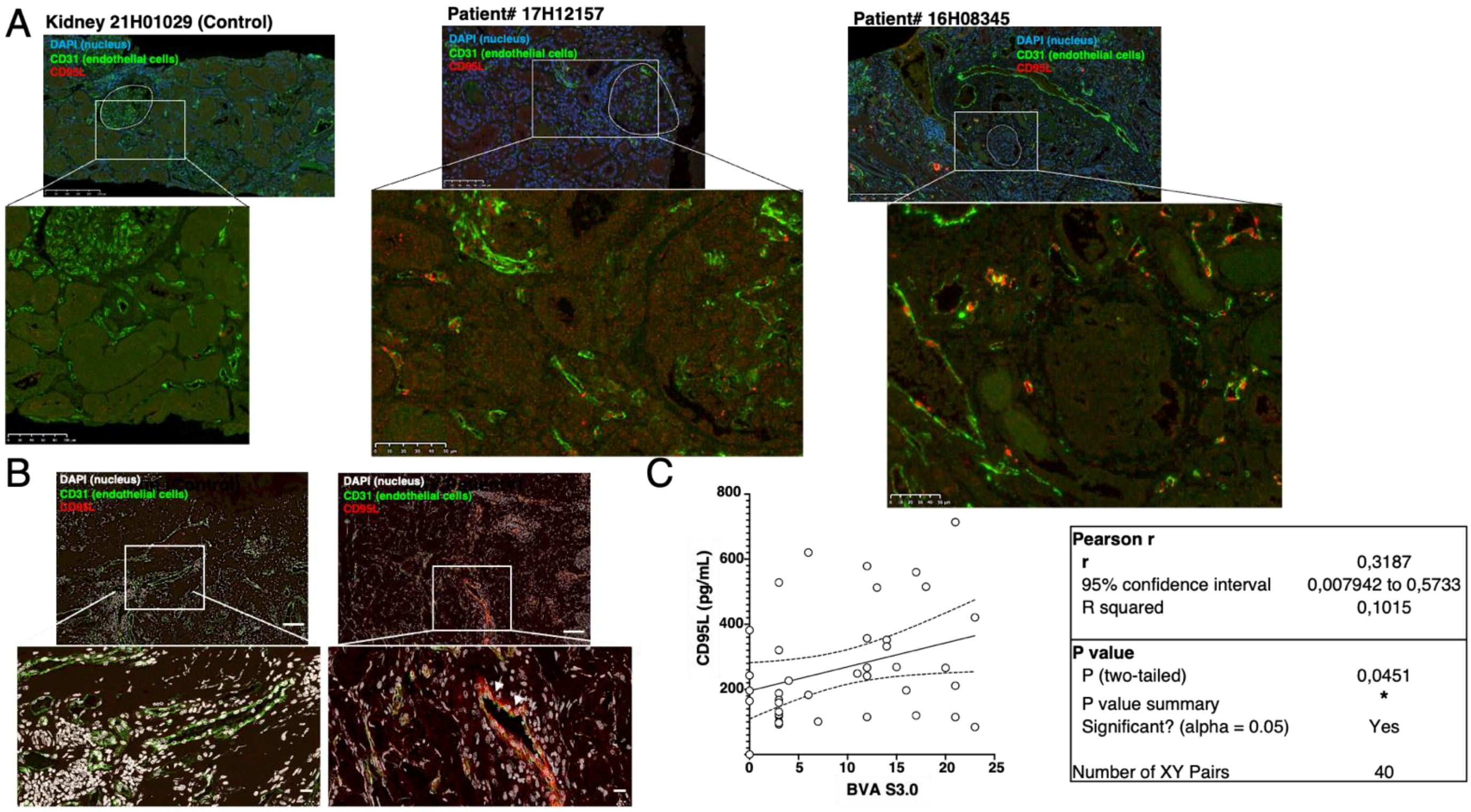

Supplementary Figure 1. Transmembrane CD95L is expressed in inflamed tissues from AAV patients.

A. CD95L *(in red)* and CD31 (marker of endothelial cells) *(in green)* staining were performed on the kidney of healthy control (21H01029, ***left panel)*** and on the damaged kidney of AAV patients (16H08345 and 17H2157*,* ***right panels)*** by immunofluorescence. Nuclei are stained in blue. Scale bars represent 250 and 100 µm in the magnified images for control kidney, 100 and 50 µm in the magnified images for Patient# 17H12157 and 250 and 50 µm in the magnified images for Patient# 16H08345 kidney.
B. CD95L *(in red)* and CD31 (marker of endothelial cells) *(in green)* staining were performed on control skin and AAV patient by immunofluorescence. Nuclei are stained in white. Scale bars represent 100 or 20 µm in the magnified images.
C. ***Left panel:*** Representation of the concentrations of sCD95L in AAV patients regarding their BVAS3.0 (disease activity score). ***Right panel:*** Pearson correlation coefficient and p value measured with linear correlation between sCD95L concentrations and BVAS3.0.

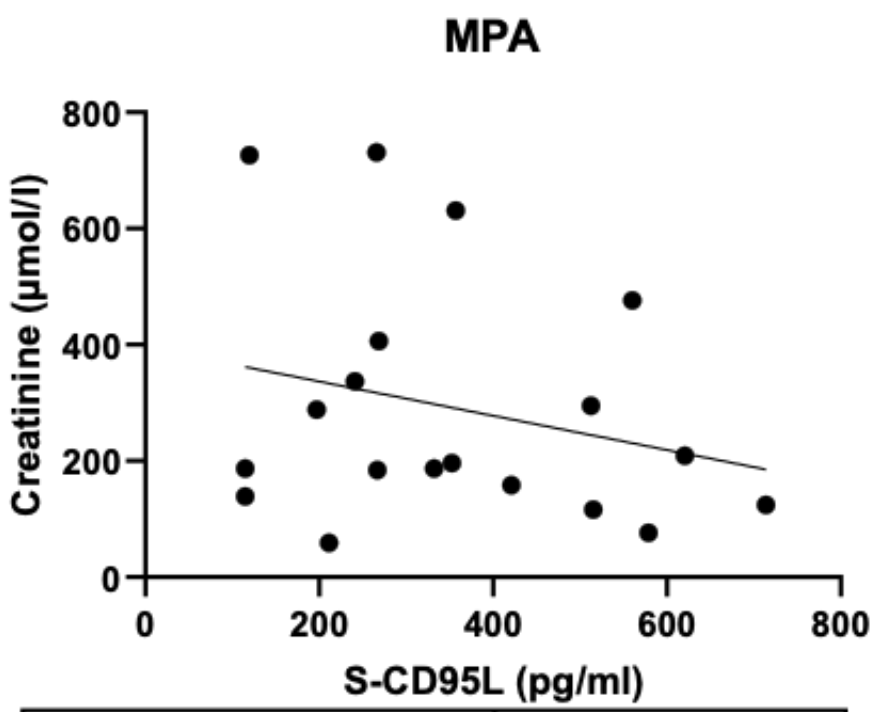

Spearman r	
r	-0.1667
95% confidence interval	-0.5868 to 0.3240
P value	
P (two-tailed)	0.4951
P value summary	ns

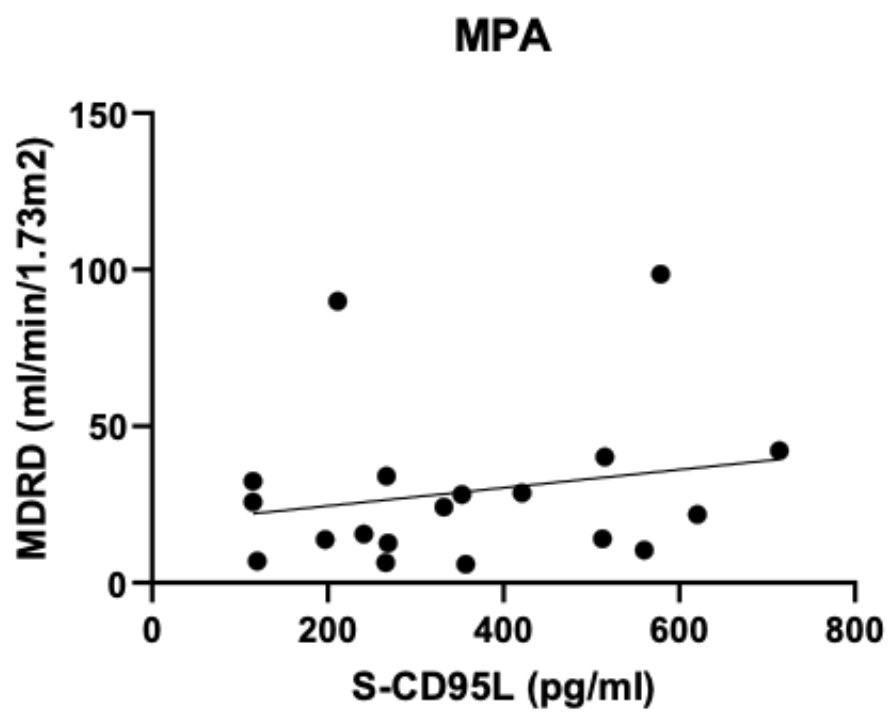

Spearman r	
r	0.1912
95% confidence interval	-0.3012 to 0.6032
P value	
P (two-tailed)	0.4329
P value summary	ns

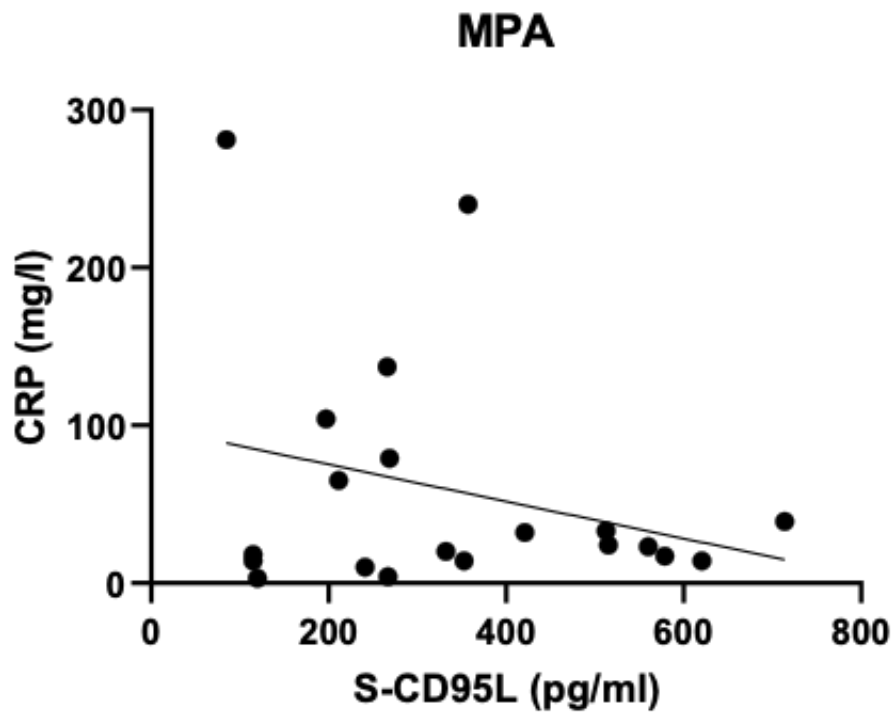

Spearman r	
r	-0.01958
95% confidence interval	-0.4692 to 0.4381
P value	
P (two-tailed)	0.9347
P value summary	ns

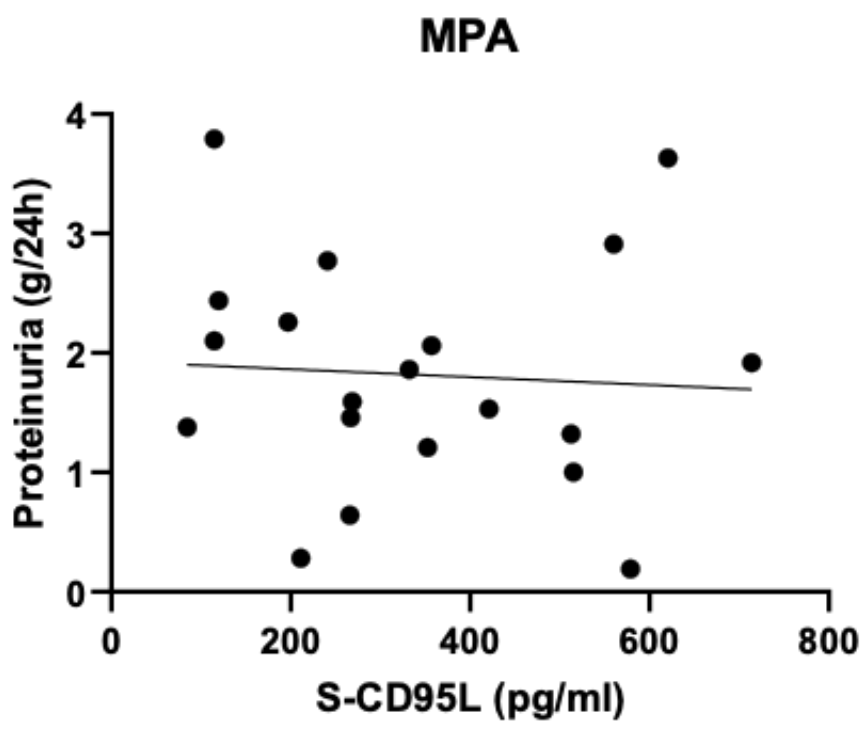

Spearman r	
r	-0.1429
95% confidence interval	-0.5603 to 0.3324
P value	
P (two-tailed)	0.5480
P value summary	ns

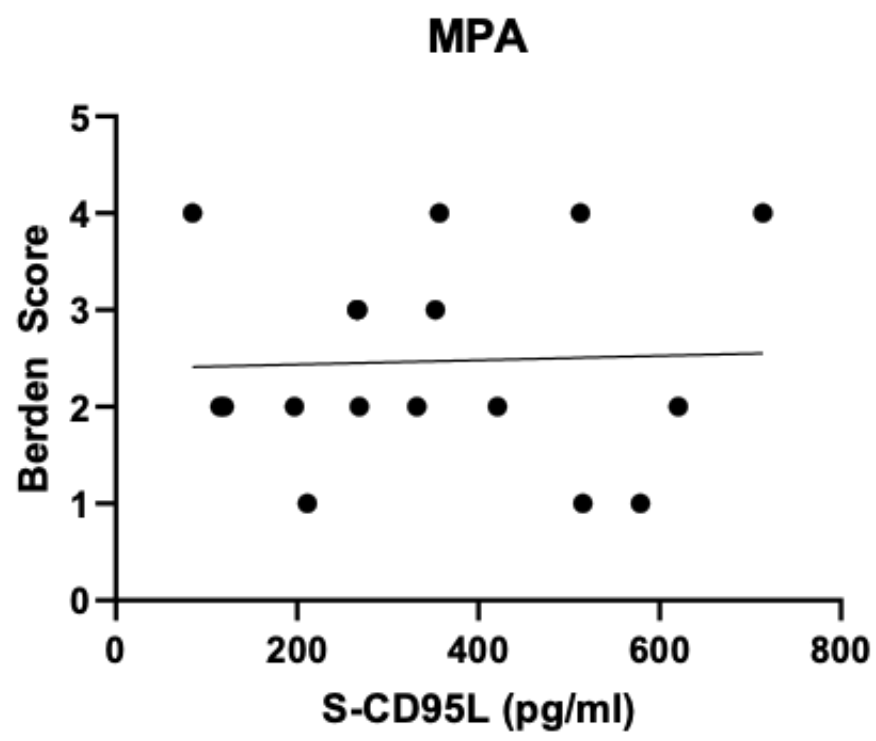

Spearman r	
r	-0.01028
95% confidence interval	-0.5002 to 0.4846
P value	
P (two-tailed)	0.9704
P value summary	ns

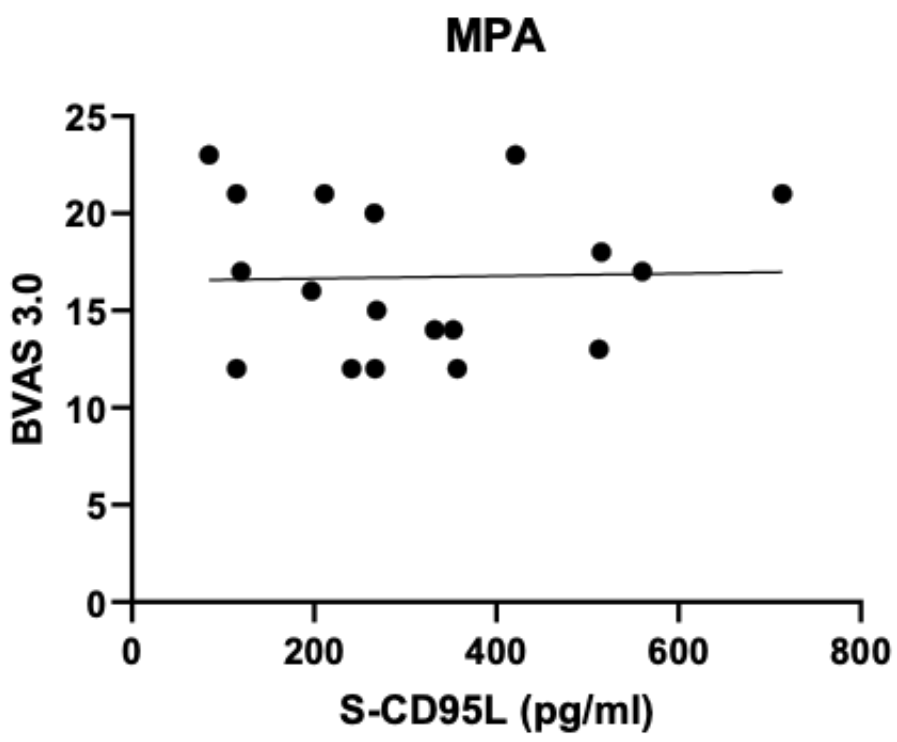

Spearman r	
r	-0.05414
95% confidence interval	-0.5192 to 0.4356
P value	
P (two-tailed)	0.8310
P value summary	ns

Supplementary Figure 2. No correlation was observed between sCD95L concentration and ANCA vasculitis clinical factors.

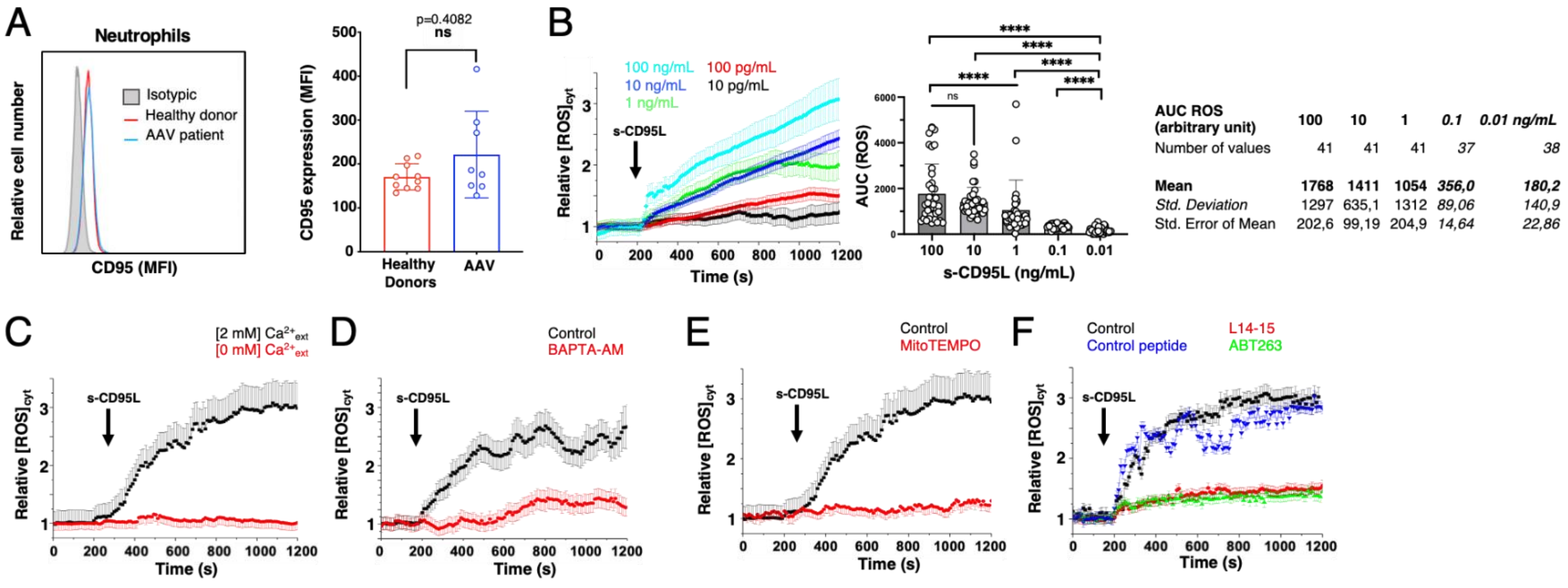

Supplementary Figure 3. The CD95-mediated Ca^{2+} response is mandatory for the ROS production.

A. ***Left panel:*** Representative histogram of the mean fluorescence intensity (MFI) of CD95 on PNN from healthy donor and AAV patient analyzed by flow cytometry. ***Right panel:*** Histogram represents mean ± SD of CD95 MFI in neutrophils from healthy donors (n=10) and AAV patients (n=8). Statistic was performed using two-tailed Mann-Whitney test.

B. ***Left panel:*** ROS production by AAV neutrophils exposed to a dose effect of soluble CD95L. Data represent the mean ± SD (n=41 cells). Data are representative of three independently performed experiments. ***Right panel:*** representation of the area under the curve (AUC) of cytosolic ROS measured in AAV neutrophils exposed to a dose effect of soluble CD95L. Data represent mean ± SD; **** p<0.0001, using two-tailed Mann-Whitney test.

C. AAV neutrophils were stimulated with sCD95L (100 ng/mL) in a calcium-free extracellular medium. The cytosolic ROS response was assessed. Data represent the mean ± SD (n=41 cells). Data are representative of three independently performed experiments.

D. Intracellular calcium was chelated using BAPTA-AM pretreatment (10 µM, 30 minutes) in AAV neutrophils, then cells were exposed to sCD95L (100ng/ml). The cytosolic ROS response was assessed. Data represent the mean ± SD (n=41 cells). Data are representative of three independently performed experiments.

E. AAV neutrophils were pre-incubated with the mitochondrion-targeted antioxidant, MitoTEMPO (10 µM, 30 minutes) and then stimulated with sCD95L (100 ng/mL). The cytosolic ROS production in AAV neutrophils was assessed. Data represent the mean ± SD (n=41 cells). Data are representative of three independently performed experiments.

F. Inhibition of the calcium transfer from ER to mitochondria with the Bcl2 inhibitor, ABT-263 (100 nM, 30 minutes), or the Bcl2/VDAC1 interaction inhibitor, L14-15 (10 µM, 30 minutes), blocks CD95-mediated (sCD95L, 100ng/ml) oxidative stress in neutrophils from vasculitis patients. Data represent the mean ± SD (n=41 cells). Data are representative of three independently performed experiments.

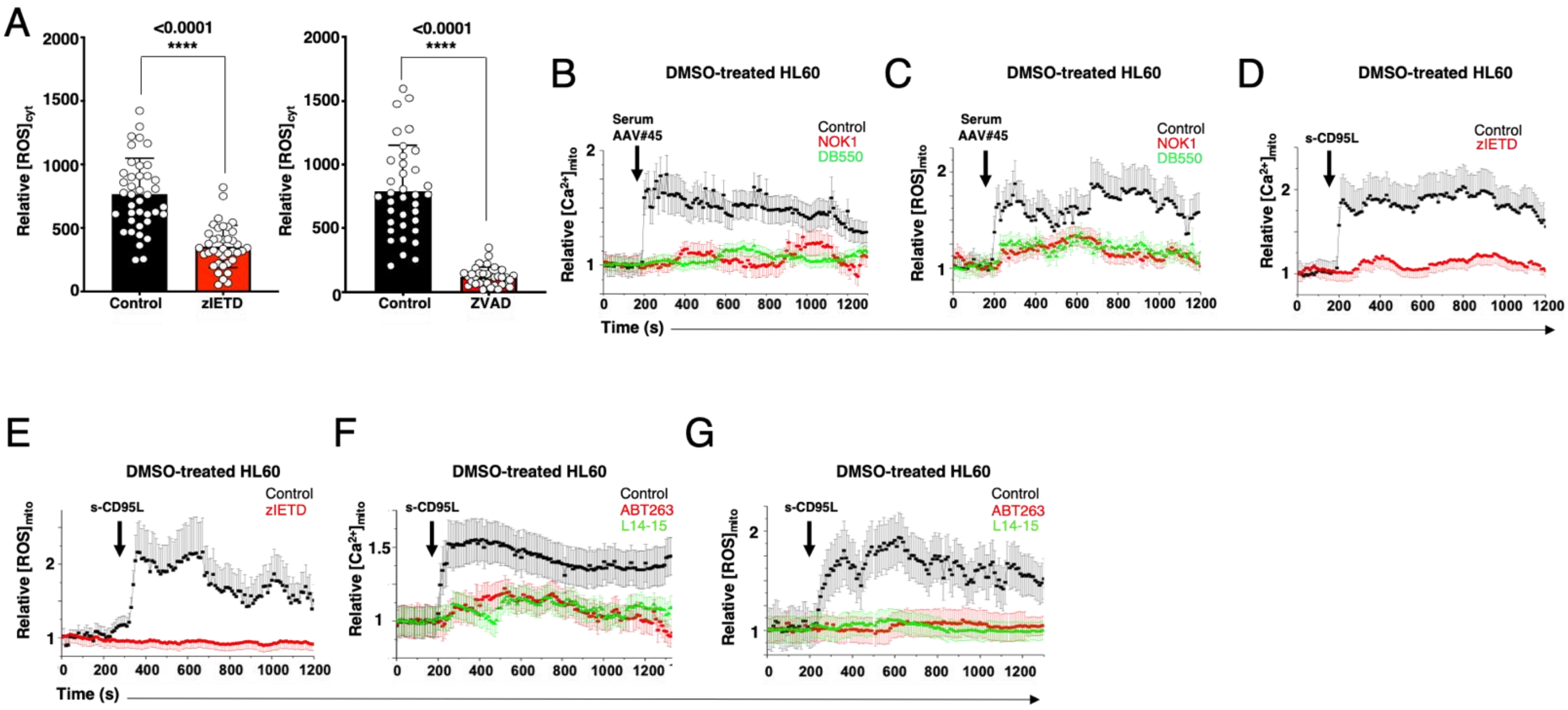

Supplementary Figure 4. Caspase-8 activity is mandatory to the mitochondrial Ca^{2+} uptake and subsequent ROS generation.

A. Histograms represent mean ± SD of area under the curve (AUC) of ROS production measured for each cell analyzed in figure 4B (AAV neutrophils loaded with Cal-520-AM, treated or not with zIETD-fmk ***(left panel***, 50 µM, 30 minutes) or zVAD-fmk ***(right panel***, 100 µM, 30 minutes) and stimulated with sCD95L (100 ng/mL)). Data represent the mean ± SD. **** p<0.0001, using two-tailed Mann-Whitney test.

B. DMSO-treated HL60 cells were stimulated with serum from AAV patient and mitochondrial Ca^{2+} concentration was assessed. Serum from AAV patient was pre-treated for 30 minutes with NOK-1 (10 µg/ml). For DB550 treatment, cells were pre-incubated 30 min with DB550 (1 µM) and then, stimulated with sCD95L (100 ng/mL). Data represent the mean ± SD (n=41 cells). Data are representative of three independently performed experiments.

C. Cells were treated as in B and mitochondrial ROS concentration was monitored. Data represent the mean ± SD (n=41 cells). Data are representative of three independently performed experiments.

D. DMSO-treated HL60 cells were pre-incubated with zIETD-fmk (50 µM) for 30 minutes and then stimulated with sCD95L (100 ng/mL). Mitochondrial Ca^{2+} concentration was assessed. Data represent the mean ± SD (n=41 cells). Data are representative of three independently performed experiments.

E. Cells were treated as in D and mitochondrial ROS were measured. Data represent the mean ± SD (n=41 cells). Data are representative of three independently performed experiments.

F. DMSO-treated HL60 cells were pre-incubated with the Bcl2 inhibitor, ABT263 (100 nM, 30 minutes), the Bcl2/VDAC1 interaction inhibitor, L14-15 (10 µM, 30 minutes) or without treatment (control) and the CD95-mediated (sCD95L, 100 ng/mL) mitochondrial Ca^{2+} concentration was assessed. Data represent the mean ± SD (n=41 cells). Data are representative of three independently performed experiments.

G. Cells were treated as in F and mitochondrial ROS was measured. Data represent the mean ± SD (n=41 cells). Data are representative of three independently performed experiments.

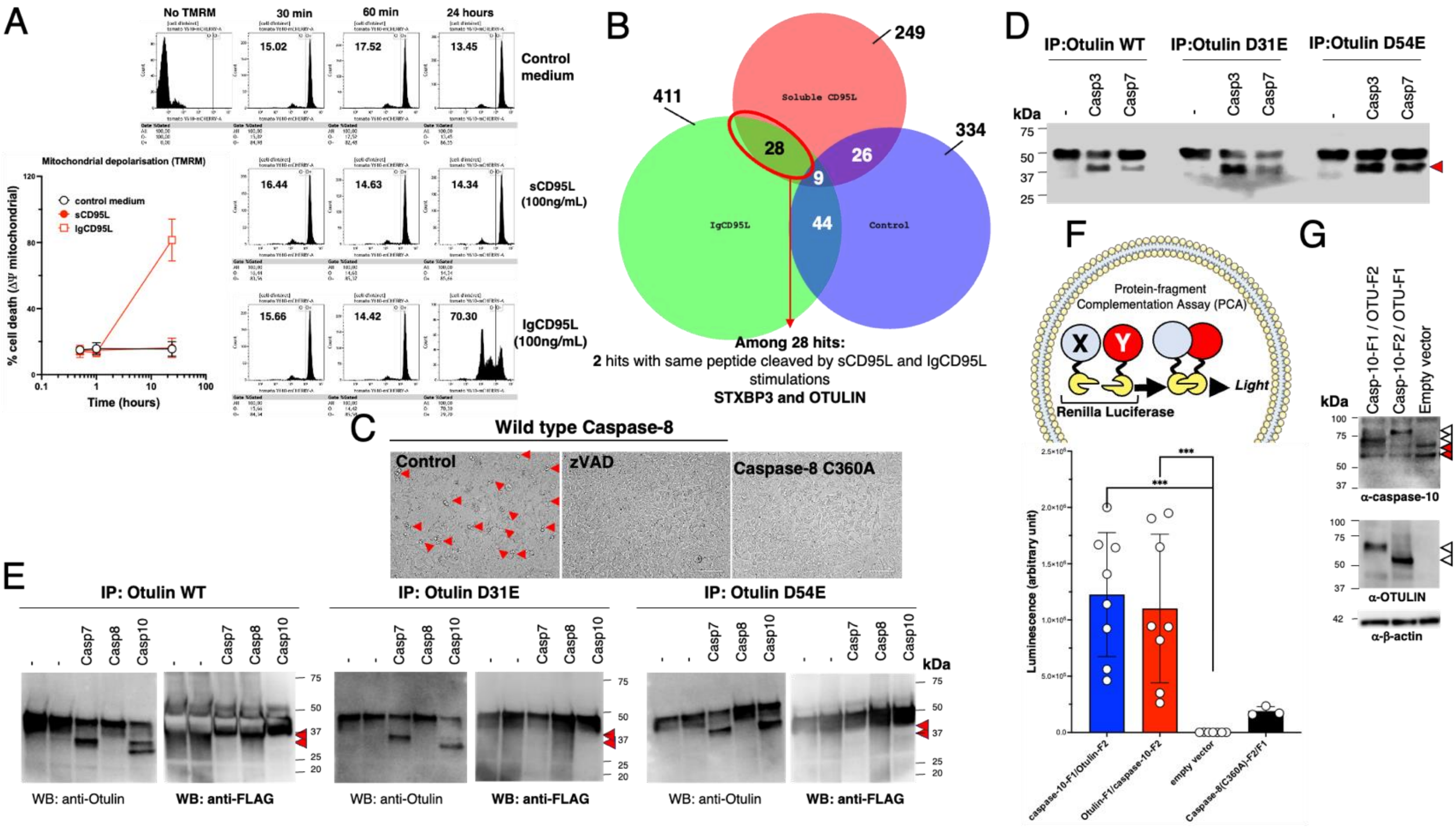

Supplementary Figure 5. OTULIN is a caspase substrate in neutrophils exposed to CD95L.

A. To assess early apoptotic events in HL60 cells exposed to sCD95L or IgCD95L, mitochondrial membrane depolarization (ΔΨm), an early marker of apoptosis, was measured by flow cytometry. DMSO-differentiated HL60 cells were pre-incubated with TMRM (40 nM) for 30 min and subsequently stimulated with vehicle, sCD95L, or IgCD95L (100 ng/mL) for the indicated time periods. Mitochondrial depolarization was evaluated by monitoring the loss of TMRM fluorescence; representative flow cytometry histograms are shown on the right. No significant cell death was detected within the first hour following stimulation with either ligand. The strong (cytotoxic) CD95 inducer IgCD95L induced marked apoptosis after 24 h of stimulation in DMSO-treated HL60 cells.

Left panel: quantification of mitochondrial depolarization (loss of ΔΨm) in HL60 cells treated with control medium, sCD95L, or IgCD95L (100 ng/mL) for the indicated times. Data are presented as mean ± SD from three independent experiments.

B. Venn diagram showing the number of peptides whose expression is modulated (positively or negatively) in the presence zVAD-fmk for indicated condition. Among the common 28 hits between IgCD95L (strong CD95 activation) and sCD95L (weak CD95 activation), only two peptides encompassing an aspartic acid (D) in their sequence were identified.

C. HEK/293T cells were transfected with wild type or catalytically inactive (C360A) caspase-8 and 24 hours later, pictures were taken. Floating cells (dead cells) are observed ***(red arrows)*** with wild type caspase-8. Scale bars represent 100 µm.

D. HEK cells were transfected with indicated construct-encoding pLVX-IRES-tdTomato vectors and 24 hours later, cells were lyzed and the indicated FLAG-tagged OTULIN constructs were immunoprecipitated using anti-FLAG-coated beads. Beads were then washed and incubated with indicated caspases for 90 minutes at 37°C. OTULIN cleavage was analyzed by immunoblot with an anti-OTULIN mAb.

E. HEK cells were transfected with indicated construct-encoding pLVX-IRES-tdTomato vectors and 24 hours later, cells were lyzed and the indicated FLAG-tagged OTULIN constructs were immunoprecipitated using anti-FLAG-coated beads. Beads were then washed and incubated with indicated caspases for 90 minutes at 37°C. OTULIN cleavage was analyzed by immunoblot with an anti-OTULIN mAb and an anti-FLAG mAb targeting the N-terminal region of OTULIN.

F. ***Upper panel:*** Schematic representation of the protein-fragment complementation assay (PCA). The *Renilla* luciferase (RLuc) enzyme was divided into amino-terminal (F1) and carboxy-terminal (F2) fragments and these cDNAs were fused to the OTULIN, caspase-8 or caspase-10 sequences. ***Lower panel:*** HEK cells were transfected with indicated constructs and 24 hours after transfection, cells (10^5 cells) were deposited in white flat bottom 96-well plate. Coelenterazine-h was added to each well and *Renilla* luciferase activity was monitored. Histograms represent mean±SD of n=3 to 8 experiments. *** $P<0.001$, using two-tailed non-parametric Mann-Whitney test.

G. The expression levels of F1 and F2-fused caspase-10 and OTULIN constructs in HEK cells used in **F** were assessed by immunoblotting using indicated antibodies. β-Actin served as a loading control. White arrows indicate caspase-10-F1 and caspase-10-F2 or OTULIN-F1 and OTULIN-F2 chimeric proteins. The red arrows show the endogenous caspase-10 in HEK/293T cells. No endogenous OTULIN was detected in HEK/293T cells. Images are representative of three independent experiments.

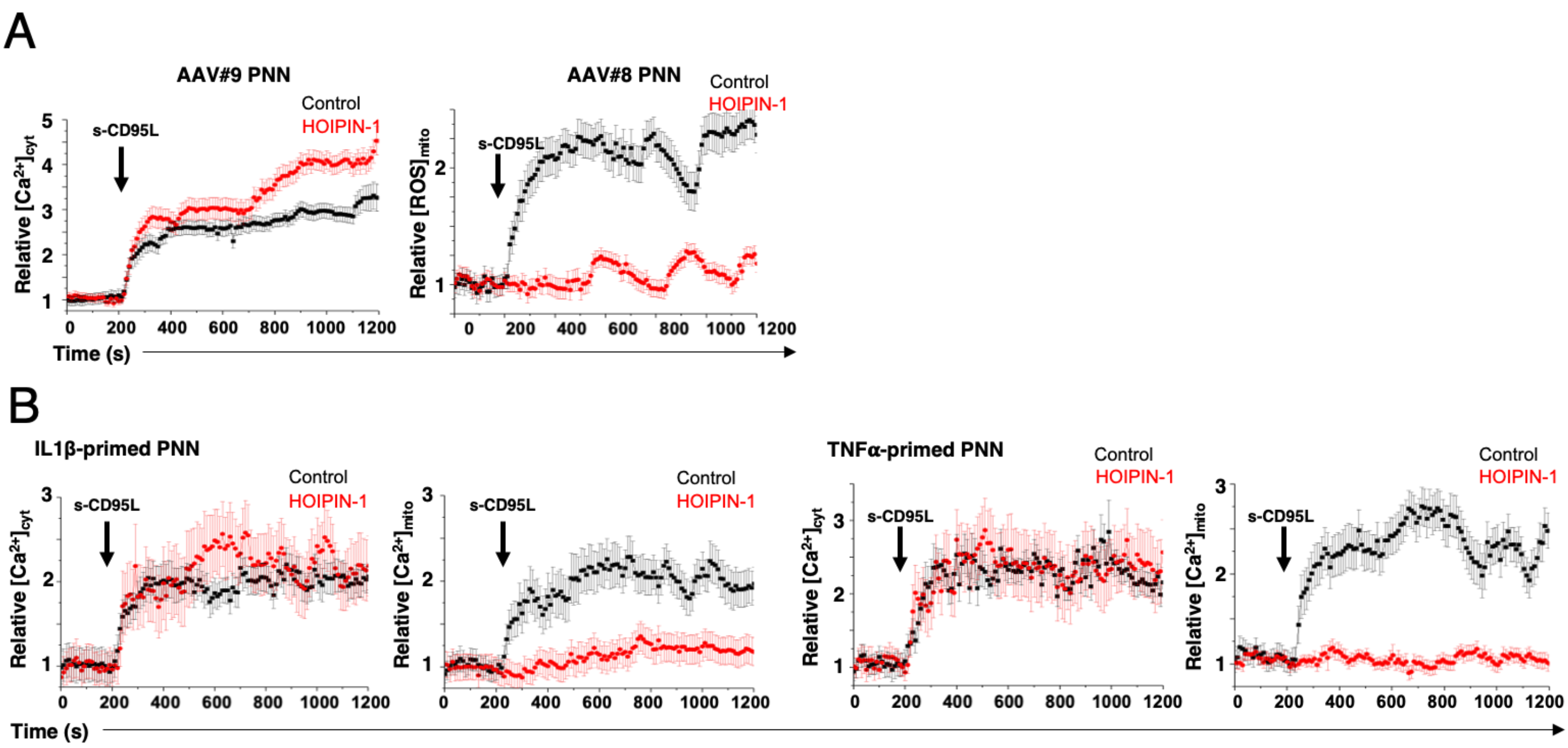

Supplementary Figure 6. LUBAC activity is involved in the CD95-mediated ROS generation in neutrophils.

A. Neutrophils isolated from AAV patient were pre-treated for 30 minutes in the presence or absence of HOIPIN-1 (10µg/ml) and then, stimulated with sCD95L (100 ng/mL). Cytosolic Ca^{2+} ***(left panel)*** and mitochondrial ROS ***(right panel)*** concentrations were assessed using Cal-520-AM and MitoSOX, respectively. Data represent the mean ± SD (n=41). Data are representative of three independently performed experiments.

B. Neutrophils from healthy donors were pre-incubated with IL-1β or TNF for 15 min in the presence or absence of HOIPIN-1 (10µg/ml) and then stimulated with sCD95L (100 ng/mL). Cytosolic ***(left panel)*** and mitochondrial ***(right panel)*** Ca^{2+} were monitored using Cal-520-AM and Rhod2-AM probes, respectively. Data represent the mean ± SD (n=41). Data are representative of three independently performed experiments.

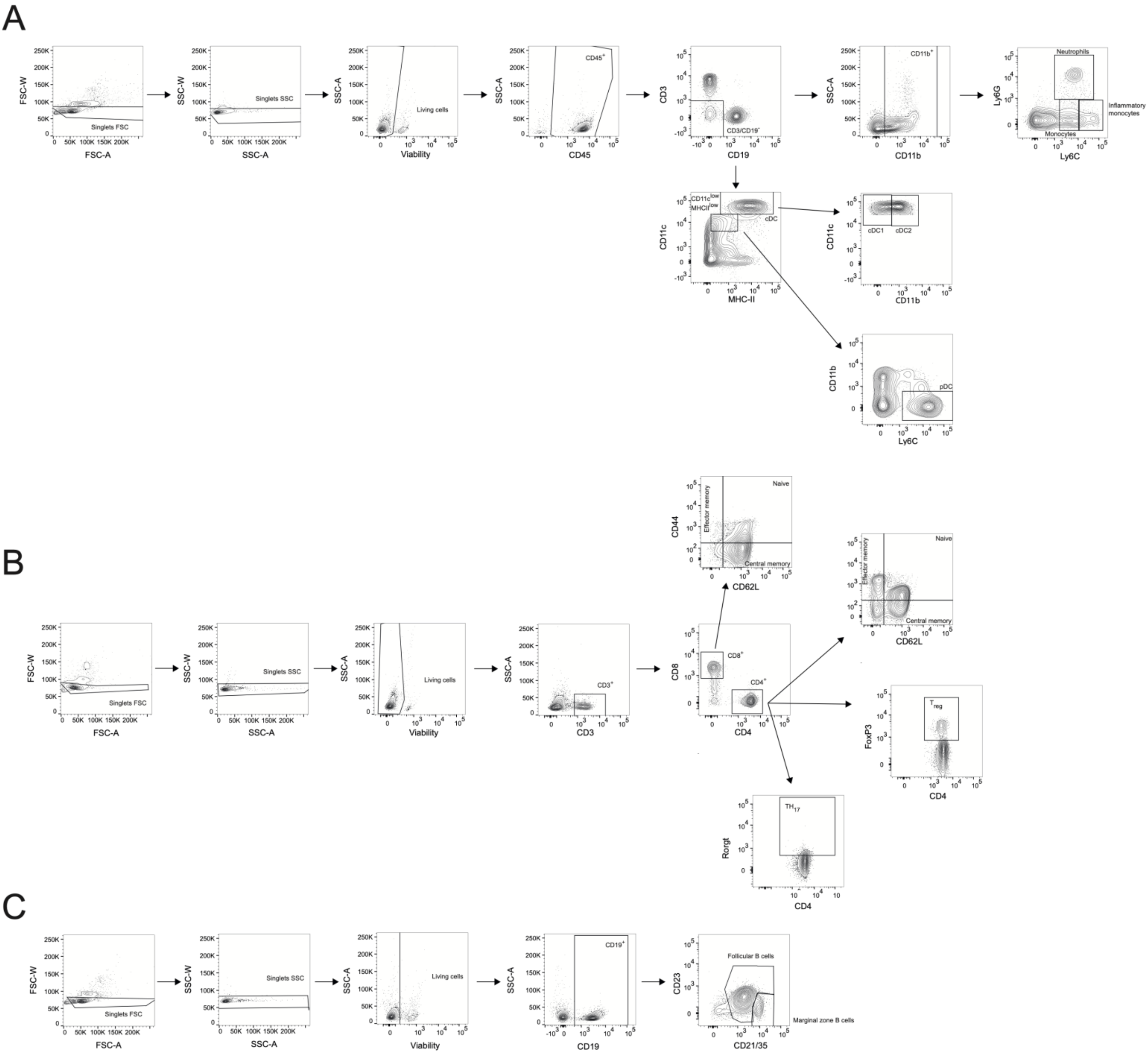

Supplementary Figure 7. Multiparameter flow cytometry analyses of immune cells in anti-MPO-induced AAV mice treated or untreated with DB550.

Representative flow cytometry plot from control mice showing immune cell gating strategy used for Table 1

A. Gating strategy for monocytes, inflammatory monocytes, neutrophils, conventional dendritic cells (cDC), cDC1 and 2.

B. Gating strategy for $CD4^+$, $CD8^+$ and subsequent naïve, effector memory and central memory cells, T_{reg} and Th_{17}.

C. Gating strategy follicular B cell and marginal zone B cells.

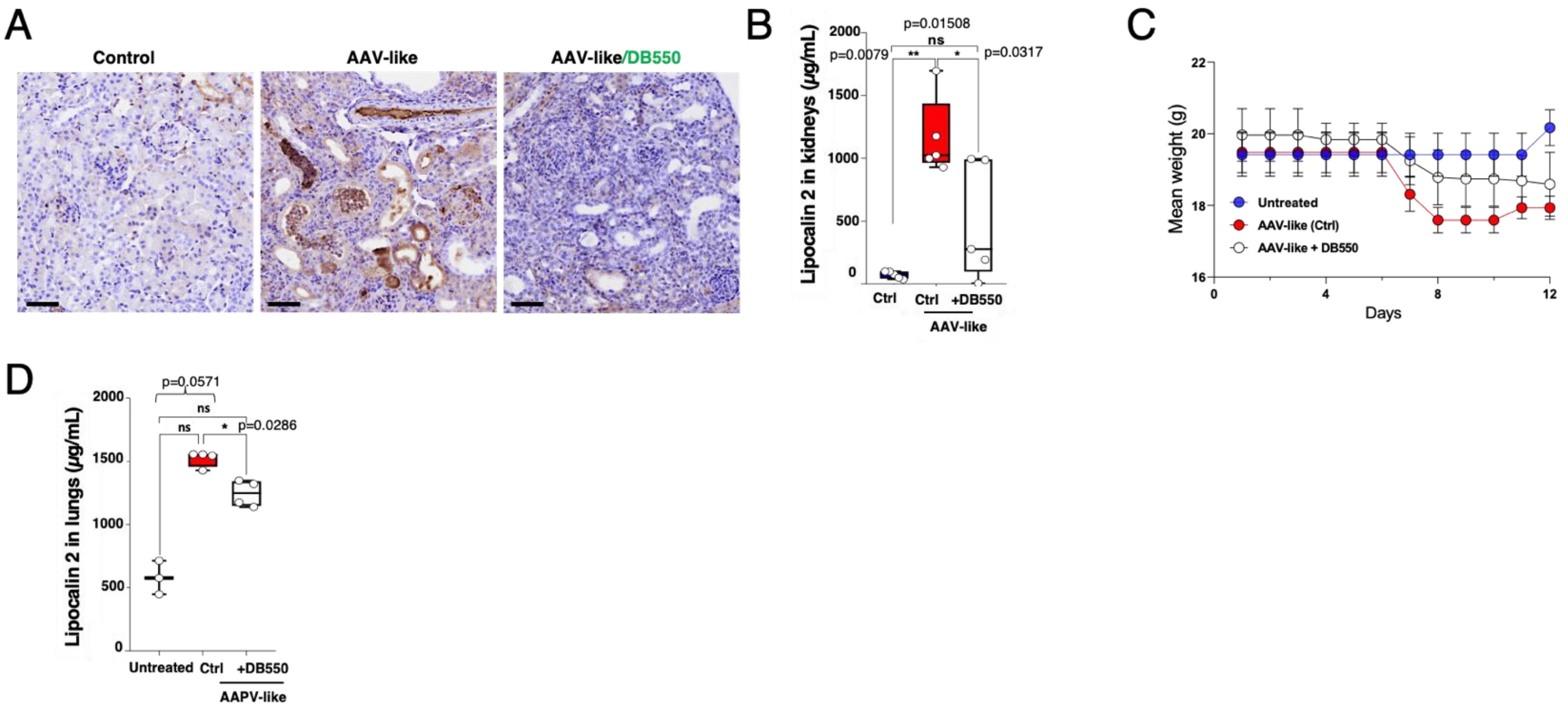

Supplementary Figure 8. Analyses of the anti-MPO-induced AAV mouse models.

A. Infiltrating neutrophils (elastase marker) in kidneys of indicated mice analyzed by IHC. Scale bars represent 50 µm.

B. Lipocalin 2 was measured by ELISA from kidney lysates of untreated, AAV-like mice and AAV-like mice treated with DB550 (n=3-4 mice per group). Data represent mean ± SD; * $p<0.05$, ** $p<0.01$, using two-tailed Mann-Whitney test.

C. Mice body weights have been measured daily for each group (n=3-4 mice per group).

D. Lipocalin 2 was measured by ELISA from lung lysates of untreated, Ctrl and DB550-treated AAPV mice (n=3-4 mice per group). Data represent mean ± SD; ns: not significant, * $p<0.05$, using two-tailed Mann-Whitney test.

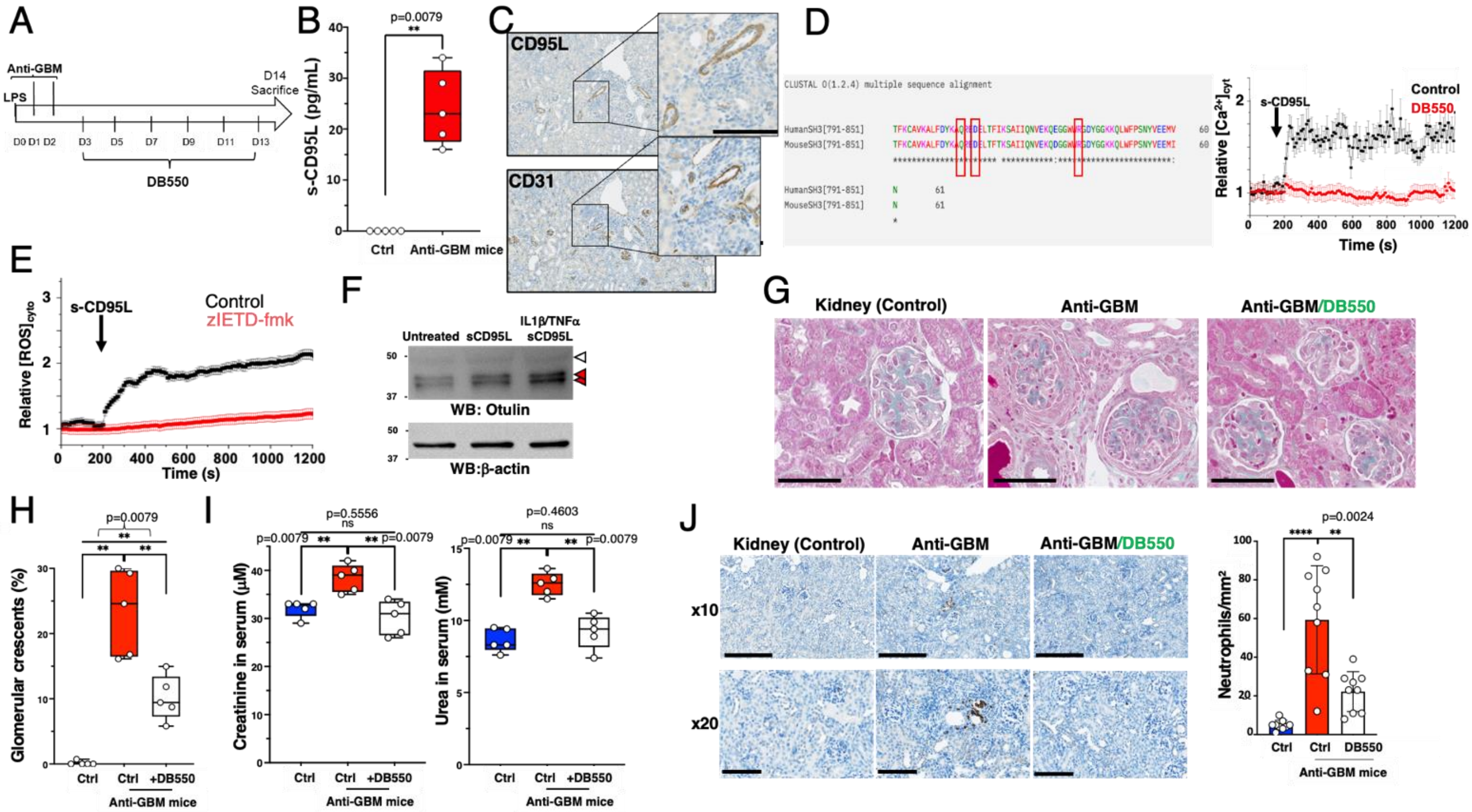

Supplementary Figure 9. Analyses of the anti-GBM-induced glomerulonephritis mouse model.

A. To induce glomerulonephritis ("anti-GBM" group), mice were treated by LPS (0.5 mg/kg) at day 0 and sheep anti-GBM nephrotoxic serum (40 mg/kg) from day 0 to day 3, or control sheep non-immune serum ("Untreated" group). Anti-GBM mice were treated ("Ctrl" group) or not with DB550 (10 mg/kg) three times per week ("+DB550" group). (n=5 mice per group).

B. CD95L was dosed by ELISA at day 14 after sheep Ig injection. Data represent mean ± SD; ** p<0.01 using unpaired and two-tailed Mann-Whitney test.

C. CD95L ***(upper panel)*** and CD31 ***(lower panel)*** staining on kidney serial sections from Anti-GBM mice (Ctrl group). Scale bars represent 100 μm.

D. ***Left panel:*** The protein sequences of SH3 domains of human and mouse PLCγ1 were aligned using CLUSTAL. Sequences are highly conserved between human and mouse SH3 domains. In addition, amino acid residues involve in the DB550 interaction are conserved (Q805, D808 and R830 highlighted in red squares)[27]. ***Right panel:*** Murine neutrophils isolated from mice injected with GMB nephrotoxic serum were pre-treated with or without DB550 (1 μM, 30 minutes) and then exposed to sCD95L (100 ng/ml). The cytosolic Ca^{2+} concentration was measured. Data represent the mean ± SD (n=41). Data are representative of three independently performed experiments.

E. Murine neutrophils isolated from mice injected with GMB nephrotoxic serum were pre-treated with or without zIETD-fmk (50 μM, 30 minutes) and then exposed to sCD95L (100 ng/ml). The cytosolic ROS concentration was assessed. Data represent the mean ± SD (n=41). Data are representative of three independently performed experiments.

F. Murine neutrophils isolated from untreated mice were pre-incubated with or without TNF and IL-1β for 15 min and then exposed to sCD95L (100 ng/ml). Cells were lyzed and indicated immunoblots were performed.

G. Representative Masson's trichrome staining of kidneys from untreated (LPS/non-nephrotoxic serum), untreated (LPS/nephrotoxic serum) and treated (LPS/nephrotoxic serum + DB550) mice. Images are representative of normal glomeruli observed in control (LPS/non-

nephrotoxic serum) mice (normal glomerulus), crescentic glomeruli in AAV-like kidneys (LPS/nephrotoxic serum) and treated glomeruli. Scale bars represent 50 µm.
H. Glomerular injury (% Glomerular crescents) was scored by pathologists in kidneys isolated from healthy mice, anti-GMB mice and anti-GBM mice treated with DB550. Data represent the mean ± SD. ** $p < 0.01$, using two-tailed non-parametric Mann-Whitney test.
I. Quantification of serum creatinine and urea in untreated mice, anti-GMB mice and anti-GBM mice treated with DB550. Data represent the mean ± SD. Ns: not significant, ** $p < 0.01$, using two-tailed non-parametric Mann-Whitney test.
J. Infiltrating neutrophils (elastase marker) in kidneys of indicated mice analyzed by IHC. Scale bars represent 250 µm and 100 µm for magnified images. The number of kidney-infiltrating neutrophils was quantified by regions of interest (ROIs) as described in *met and Methods*. Each ROI covers 6 mm^2. Data represent the mean ± SD. ** $p<0.01$, **** $p<0.0001$ using two-tailed non-parametric Mann-Whitney test. Space bars = 250µm.

Supplementary Table 1. Description of HD and AAV patient cohort (Bordeaux University Hospital).
General symptoms = fever, fatigue, loss of weight, loss of appetite; ENT = Ear, Nose and Throat
* = Treatment ever received

Clinical characteristics	Healthy Donors (N=20)	EGPA (N=12)	GPA (N=19)	MPA (N=39)
Age (Mean [SD])	61.5 [1.6]	67.1 [10.0]	57.5 [13.2]	66.2 [10.8]
Men (% [absolute number])	75.0 [15]	25.0 [3]	73.7 [14]	74.4 [29]
Woman (% [absolute number])	25.0 [5]	75.0 [9]	26.3 [5]	25.6 [10]
General symptoms (% [absolute number])	NA	41.7 [5]	52.6 [10]	30.8 [12]
Kidney (% [absolute number])	NA	33.3 [4]	73.7 [14]	97.4 [38]
Pulmonary (% [absolute number])	NA	75.0 [9]	84.2 [16]	64.1 [25]
Cutaneous (% [absolute number])	NA	25.0 [3]	5.3 [1]	7.7 [3]
Neurological (% [absolute number])	NA	91.7 [11]	47.4 [9]	15.4 [6]
Muscular (% [absolute number])	NA	33.3 [4]	5.3 [1]	12.8 [5]
Joint (% [absolute number])	NA	33.3 [4]	57.9 [11]	28.2 [11]
ENT (% [absolute number])	NA	66.7 [8]	68.4 [13]	33.3 [13]
Digestive (% [absolute number])	NA	0.0 [0]	0.0 [0]	10.3 [4]
Ophthalmologic (% [absolute number])	NA	0.0 [0]	21.1 [4]	0.0 [0]
Cardiovascular (% [absolute number])	NA	16.7 [2]	10.5 [2]	7.7 [3]
Associated cancer or hematologic malignancies (% [absolute number])	NA	0.0 [0]	10.5 [2]	0.0 [0]
c-ANCA Anti-PR3 (% [absolute number])	NA	0.0 [0]	89.5 [17]	10.3 [4]
p-ANCA Anti-MPO (% [absolute number])	NA	100 [12]	10.5 [2]	94.9 [37]
Corticosteroids*	NA	91.7 [11]	100 [19]	97.4 [38]
Cotrimoxazole*	NA	0.0 [0]	57.9 [11]	25.6 [10]
Cyclophosphamide*	NA	58.3 [7]	78.9 [15]	84.6 [33]
Rituximab*	NA	25.0 [3]	68.4 [13]	43.6 [17]
Methotrexate*	NA	16.7 [2]	21,1 [4]	2.6 [1]
Azathioprine*	NA	50.0 [6]	52.6 [10]	53.8 [21]
Intravenous Immunoglobulin*	NA	50.0 [6]	15.8 [3]	0.0 [0]
Plasmatic exchange*	NA	0.0 [0]	5.3 [1]	35.9 [14]

Supplementary Table 2. Description of longitudinal AAV patient cohort (Angers University Hospital).
General symptoms = fever, fatigue, loss of weight, loss of appetite; ENT = Ear, Nose and Throat
* = Treatment received at 6 months post-diagnosis

**Clinical characteristics**	**MPA (N=20)**
Age (Median [SD])	60,6 [13.5]
Men (% [absolute number])	45.0 [9]
Woman (% [absolute number])	55.0 [11]
General symptoms (% [absolute number])	65.0 [13]
Kidney (% [absolute number])	100 [20]
Pulmonary (% [absolute number])	40.0 [8]
Cutaneous (% [absolute number])	15.0 [3]
Neurological (% [absolute number])	10.0 [2]
Muscular (% [absolute number])	25.0 [5]
Joint (% [absolute number])	30.0 [6]
ENT (% [absolute number])	35.0 [7]
Digestive (% [absolute number])	0.0 [0]
Ophthalmologic (% [absolute number])	10.0 [2]
Cardiovascular (% [absolute number])	5.0 [1]
Associated cancer or hematologic malignancies (% [absolute number])	20.0 [4]
c-ANCA Anti-PR3 (% [absolute number])	0.0 [0]
p-ANCA Anti-MPO (% [absolute number])	90.0 [18]
Corticosteroids*	100 [20]
Cotrimoxazole*	85.0 [17]
Cyclophosphamide*	80.0 [16]
Rituximab*	80.0 [16]
Methotrexate*	5.0 [1]
Azathioprine*	35.0 [7]
Intravenous Immunoglobulin*	0.0 [0]
Plasmatic exchange*	45.0 [9]

Supplementary Table 3: Description of the patients used for immunoblot Figure 6F.
M: Male / F: Female;
MPA: Microscopic polyangiitis / GPA: Granulomatosis with polyangiitis;
K: Kidney / Neur: Neurological / Sk: Skin / Lu: lung / No: Nose;
MPO: Anti-myeloperoxydase (UI/ml) / PR3: anti-proteinase 3 (UI/ml);
A: Active / I: Inactive;
BVAS: Birmingham Vasculitis Activity Score;
Cs: Steroids / Cyc: cyclophosphamide / Rtx: Rituximab.

Patient	Age	Sex	Disease	Symptoms	ANCA	Status	BVAS	Treatment
1	68	M	MPA	K	MPO (1776)	A	14	Cs, Cyc, Rtx
2	72	F	GPA	Sk, Lu, K, Gut	PR3 (672)	A	31	Cs, Cyc, Rtx
3	64	F	MPA	K, Neur	MPO (135)	I	4	Cs
4	69	M	GPA	No, Eye	PR3 (144)	I	0	Cs
5	64	M	MPA	K, Neur	MPO (126)	A	25	Cs, Cyc, Rtx
6	75	M	MPA	K, Lu, Sk	MPO (23)	I	3	Cs
7	70	F	MPA	Lu, Neur	MPO (15)	A	12	Cs, Cyc
8	75	M	MPA	K	MPO (30)	A	12	Cs, Rtx
9	53	M	MPA	K, Neur	MPO(27)	I	2	Cs
10	89	M	MPA	K	MPO (4298)	A	12	Cs, Rtx

Supplementary Table 4: Reagents and dilutions.

Reagent	Cat number	dilution/concentration	Fournisseur	Ville/pays
ABT-263	CT-A263		Chimietek	Indianapolis, IN, USA
Alexa Fluor 488-conjugated anti-human CD95 (DX2)	305616		Biolegend	San Diego, CA, USA
Alexa Fluor 700 anti-mouse CD23 Antibody	101632	1:200	Biolegend	San Diego, CA
Alexa Fluor 700 anti-mouse CD45 Antibody	103128	1:200	Biolegend	San Diego, CA
Alexa Fluor 633-congated goat anti-Mouse IgG (H+L)	A21052		Invitrogen	Waltham, MA, USA
Anti caspase-8 human mAb clone 12F5	ALX-804-242-C100	1/3000	Enzo	Farmingdale, NY, USA
Anti-b-actin	A5441	1/5000	Sigma Aldrich Chimie	Saint Louis, MI, USA
Anti-b-actin	A3854		Sigma-Aldrich	Saint Louis, MI, USA
Anti-caspase 3 (D3R6Y)	14220		Cell Signaling	Beverly, MA, USA
Anti-caspase-8 (1C12)	9746		Cell Signaling	Beverly, MA, USA
Anti-CD31	AB182981		Abcam	Cambridge, UK
Anti-CD31	Ab28364		Abcam	Cambridge, UK
anti-CD95 Pacific Blue	305619		Biolegend	San Diego, CA, USA
Anti-CD95L	BS-0216R		Bioss	Woburn, MA, USA
Anti-CD95L (G247-4)	556387		BD Pharmingen	Franklin Lakes, NJ, USA
anti-FADD	556402		BD Bioscience	Franklin Lakes, NJ, USA
anti-FLAG M2	F3165	1/5000	Sigma Aldrich Chimie	Saint Louis, MI, USA
anti-FLAG mAb-coated magnetic beads (M2)	8823		Sigma Aldrich	Saint Louis, MI, USA
Anti-GMB nephrotoxic serum	PTX-001S		Probetex	San Antonio, TX, USA
Anti-human Caspase 10 (63131)	MAB834	1/1000	R&D system	Minneapolis, USA
anti-human CD95 Alexa Fluor 488	305616		Biolegend	San Diego, CA, USA
Anti-mouse CD16/CD32 Fc Block Clone 2.4G2	15877028		BD Pharmingen (553142)	France
Anti-mouse IgG-HRP	1706516		BIO-RAD	Paris, France
Anti-mouse IgG1-HRP	1070-05		Southern Biotech	Birmingham, AL, USA
anti-mouse IgG1human ads-HRP	1070-05	1/3000	Southern Biotech	Birmingham, AL, USA
anti-mouse IgG2a human ads-HRP	1080-05	1/3000	Southern Biotech	Birmingham, AL, USA
Anti-Neutrophil Elastase	NBP2-57576		Novus biologicals	Centennial, CO, USA
Anti-Neutrophil Elastase	JF098-6		Novus biologicals	Centennial, CO, USA
Anti-otulin	14127	1/5000	Cell Signaling	Beverly, MA, USA
Anti-Otulin (E3B8L)	74125S	1/1000	Cell Signaling	Beverly, MA, USA
Anti-rabbit IgG-HRP	4090-05	1/3000	Southern Biotech	Birmingham, AL, USA
Anti-rabbit IgG-HRP	MP-7451		Eurobio	Les Ulis, France
APC anti-mouse CD3 Antibody	100236	1:200	Biolegend	San Diego, CA
APC/Cyanine7 anti-mouse/human CD11b Antibody	101226	1:200	Biolegend	San Diego, CA

BAPTA-AM	A1076		Sigma-Aldrich	Saint Louis, MI, USA
Brilliant Violet 421 anti-mouse CD3 Antibody	100228	1:400	Biolegend	San Diego, CA
Brilliant Violet 605 anti-mouse CD8a Antibody	100744	1:400	Biolegend	San Diego, CA
Brilliant Violet 785 anti-mouse CD19 Antibody	115543	1:100	Biolegend	San Diego, CA
Brilliant Violet 785 Mouse IgG2a, κ Isotype Ctrl Antibody	400274	1:50	Biolegend	San Diego, CA
BV786 Mouse Anti-Mouse RORγt	564723	1:50	BD Pharmingen	Franklin Lakes, NJ, USA
C5a	300-70B		Peprotech	Waltham, MA, USA
Coelenterazin H	C6780	2µg/mL	Thermo Fisher	Waltham, Massachusetts USA
FITC anti-mouse Ly-6G Antibody	127606	1:200	Biolegend	San Diego, CA
FITC Rat Anti-Mouse CD4	553047	1:200	BD Pharmingen	Franklin Lakes, NJ, USA
Fixable Viability Dye eFluor780	13539140		eBiosciences	Santa Clara, CA, USA
Fluoromount	ADI-950-260-0025		Enzo Life Sciences	Farmingdale, NY, USA
Foxp3 / Transcription Factor Staining Buffer Set	00-5523-00		Thermofisher	Waltham, Massachusetts USA
HOIPIN-1	#JTP-0819958		MedChemExpress	Monmouth Junction, NJ, USA
IgG1k-Pacific Blue	400151		Biolegend	San Diego, CA, USA
IL1b	200-01B		Peprotech	Waltham, MA, USA
InVivoMAb anti-mouse myeloperoxidase (MPO) 6D1	BE0393		Bio X cell	Paris, France
InVivoMAb anti-mouse myeloperoxidase (MPO) 6G4	BE0392		Bio X cell	Paris, France
Isotype IgG2a	BE0086		Bio X cell	Paris, France
Isotype IgG2b	BE0085		Bio X cell	Paris, France
L14-15	LAWTAGNSNTR	peptide synthesis	LifeTein	Somerset, NJ, USA
LCN-2 Mouse DuoSet	DY1857		R&D system	Minneapolis, USA
Lipopolysaccharide (LPS Escherichia coli O55:B5)	L6529		Sigma Aldrich	Saint Louis, MI, USA
M.O.M Blocking Reagent	MKB-2213-1		Vector Laboratories	Newark, USA
MitoSOX Red Reagent	M36008		Life technologies	Carlsbad, CA, USA
MitoTempo	SML0737		Sigma-Aldrich	Saint Louis, MI, USA
Pacific Blue anti-mouse I-Ab Antibody	116422	1:200	Biolegend	San Diego, CA
PE anti-mouse CD11c Antibody	117308	1:200	Biolegend	San Diego, CA
PE FOXP3 Monoclonal Antibody	12-5773-82	1:50	Thermofisher	Waltham, Massachusetts USA
PE Rat IgG2a, κ Isotype Ctrl Antibody	400508	1:50	Biolegend	San Diego, CA
PE-Cy7 Rat Anti-Mouse CD62L	560516	1:200	BD Pharmingen	Franklin Lakes, NJ, USA
PE/Cyanine7 anti-mouse CD21/CD35 (CR2/CR1) Antibody	123420	1:1000	Biolegend	San Diego, CA
PE/Dazzle 594 anti-mouse Ly-6C Antibody	128044	1:400	Biolegend	San Diego, CA
PerCP-Cy5.5 Rat Anti-Mouse CD44	560570	1:400	BD Pharmingen	Franklin Lakes, NJ, USA
RIPA buffer	sc-24948		CliniSciences	Nanterre, France
s-CD95L	310-03H		Peprotech	Waltham, MA, USA
sheep non-immune serum	PTX-000S		Probetex	San Antonio, TX, USA
TNFa	300-01A		Peprotech	Waltham, MA, USA
TSA Fluorescein reagent	NEL741001KT		Akoya Bioscience	Massachusetts, USA

V5-Tag antibody (clone SV5-Pk1)	MCA1360	1/5000	Biorad	Marnes-la-Coquette, France
Viobility 405/520 Fixable Dye	130-130-421	1:100	Miltenyibiotec	Bergisch Gladbach Germany
z-AEVD-fmk (Caspase-10 inhibitor)	FMK-009		Tocris Bioscience	Bristol, UK
zDEVD-fmk (caspase-3 inhibitor)	TA-T6005		TargetMol	Boston, MA, USA
zIETD-fmk (caspase-8 inhibitor)	FMK-007		Tocris Bioscience	Bristol, UK
zVAD-fmk (pan-caspase inhibitor)	2163/1		Tocris Bioscience	Bristol, UK
