# Supplementary material for: Soluble CD95L triggers Caspase-10-driven reactive oxygen species production in neutrophils and aggravates anti-neutrophil cytoplasmic antibody-vasculitis
Source: Nat Commun. 2026 Jun 20;17:7759. doi: 10.1038/s41467-026-74452-8 (PMC13434393; doi:10.1038/s41467-026-74452-8)
Supplement: Supplementary file 2 [file 41467_2026_74452_MOESM2_ESM.pdf]

nature portfolio

Peer Review File

Soluble CD95L triggers Caspase-10-driven reactive oxygen species production in neutrophils and aggravates anti-neutrophil cytoplasmic antibody-vasculitis

Corresponding Author: Dr Patrick Legembre

This file contains all reviewer reports in order by version, followed by all author rebuttals in order by version.

Version 0:

Reviewer comments:

Reviewer #1

(Remarks to the Author)
In this study the authors investigate sCD95L induced neutrophil ROS generation, and identify that this may play a role in AAV. The study shows expression of CD95L on endothelial cells at tissue sites of vasculitis and identifies elevated serum levels in patients with AAV with highest levels in patients with active MPA-AAV. sCD95L induces ROS in neutrophils from patients with AAV or primed healthy donor neutrophils and this is shown to be dependent on mitochondrial calcium flux and caspase-8. Proteomics of CD95L-stimulated HL60 cells identifies OTULIN as a potential caspase substrate mediating this effect. The authors then use a series of cell free experiments to dissect the molecular pathways showing OTULIN cleavage is likely due to caspase-10, with caspase-8 acting upstream. Finally, inhibition of sCD95L is shown to be an effective treatment in mice with accelerated nephrotoxic nephritis, passive anti-MPO glomerulonephritis, and pulmonary vasculitis. I thought the study was interesting, offering new insights into endothelial-neutrophil interactions in AAV. The mechanistic studies were comprehensive, dissecting out various elements of the caspase pathways.
Major Comments
1. My main criticism is that the range of different stimulation methods/cell types used sometimes made the study difficult to follow. Experiments were variously carried out using AAV serum stimulation of healthy neutrophils, sCD95L stimulation of AAV neutrophils and primed healthy neutrophils, and sCD95L stimulation of HL60 cells. Different mechanistic elements are then carried out in some but not all of the different experimental set ups. I would suggest restructuring to focus on one experimental set up at a time. In particular a smaller number of experiments are carried out using the AAV serum stimulations so focussing more on AAV neutrophils/primed healthy neutrophils for identification of mechanisms would be one option.
2. The time point of experiments and short neutrophil lifespan is given as justification for using HL-60 cells over primary neutrophils- however even for the proteomic study only a 30 minute incubation period was used which is well within a neutrophils in vitro lifespan. The HL60 cells behave differently to healthy primary cells and respond to sCD95L to a greater degree than primary neutrophils and constitutively cleave otulin. I would suggest where possible, other than experiments using Crispr KO which I appreciate may be technically challenging in primary cells, experiments should be repeated in primary cells (such as Fig 5A). For any studies which it is not possible to repeat (such as the proteomics) this should be discussed as a limitation.
3. ANCA IgG is well described to induce ROS so it is interesting that in the serum stimulation experiments blocking sCD95L results in almost complete ROS inhibition. It would be useful to show what the effect on ROS generation is if IgG is depleted from the serum. Similarly, what were the sCD95L levels in the specific sera used for these studies? And which cohort of patients do they represent; are they from patients with both MPA and GPA and what was their disease activity?
4. The elevated levels of sCD95L in active MPA compared to GPA is interesting but not discussed further. Can the authors offer a hypothesis as to why this might be the case? This association should also be corrected for confounders such as renal function, disease severity, or inflammatory state. It would also be useful to carry out multivariable analysis for the association with BVAS. Are data available on eGFR or CRP?
5. Some studies have identified that AAV neutrophils produce less ROS than those from healthy donors (PMID: 37947315, 27102815). One study also suggests that MPA neutrophils generate less ROS than GPA patients (albeit in response to non physiological stimulus with PMA) which seems contrary to the higher levels of sCD95L in MPA. It would be useful to include some discussion on this. Were the AAV neutrophils used in the study from patients with both MPA and GPA?
6. Figure 6- What effect does caspase or otulin ko have on calcium flux or cytosolic ROS? Similarly when the range of

caspase inhibitors are used in primed healthy neutrophils what impact does that have on mitoROS/calcium?
7. Accelerated nephrotoxic nephritis is a model of immune complex GN using a planted alloantigen and does not represent either anti-GBM disease or AAV. In particular it is not pauci-immune and the histopathological lesion has several differences to AAV in humans. I would avoid referring to it as a model of AAV and would suggest referring to the model as either NTN or crescentic GN. As the authors have used two other models in mice which are more representative of AAV I would suggest moving the NTN experiment to the supplemental figures.
8. Are serum CD95L levels raised in either of the vasculitis models studied?

Minor comments
1. Fig 1A- an image of a glomerulus in HC tissue should be included rather than just tubulointerstitium
2. Is there any significant neutrophil infiltrate in the passive AAV GN model?

Reviewer #2

(Remarks to the Author)
The manuscript by Boizard-Moracchini and colleagues examines the effects of soluble CD95L on Casp10-driven ROS in neutrophils and the relationship to ANCA. The manuscript is of interest for understanding disease progression but lacks key controls. The introduction should provide better context for why these findings are not incremental when compared to published studies addressing roles for FasL and neutrophils in ANCA.

Figure 1A requires a higher magnification image together with quantitation for a measure of colocalization between CD31 and CD95L. What other cells could also be present in these areas to also express CD95L? Any CD45+? It appears likely it is endothelial but at this magnification it is difficult to be confident in this disease setting.

Figure 2E. The difference in ROS production may be explained by differences in neutrophil activation from healthy controls or AAV patients. A phenotypic characterization of these neutrophils is required to account for these likely differences.

The Figure legends (for S2 as an example) imply the mean +/- SD of 41 biological replicates is shown. Were these performed on different days? Or are these technical replicates from a smaller number of biological replicates? The majority of graphs plot ‘relative ROS’, for example, so how is the relative signal compared to other positive controls that are known to generate high levels of ROS in this assay?

The authors state that the proteomic assays was performed in HL-60 cells because primary neutrophils have a short half-life, despite conducting the majority of experiments in primary neutrophils. While neutrophils can be maintained in culture for >24h with pro-survival cytokines, the proteomics assay was only performed for 30 min, so was the justification more centered around the challenges of executing proteomics on primary neutrophils rather than life span?

Figure 4F is uninterpretable at this size. The text also refers to this as a cell death assay where the homemade dodecameric IgCD95L ‘induced a strong caspase-dependent cell death signal’. The assay is only performed for 30 min so authors should provide biochemical and cellular evidence that this indeed distinguishes apoptotic and non-apoptotic cell death. It is already established in the literature that the strength of FasL signal on neutrophils determines the kinetics of cell death and the proportion of the population entering into apoptosis. Are the effects in Figure 4F and elsewhere a consequence of kinetic changes in the neutrophil population? These are important considerations when comparing different types of FasL, a potent inducer of human neutrophil apoptosis.

The proteomics in Figure S4 refers to the appearance of 2 peptides. This is a non-standard depiction of mass spec data. Additional description of the results of the mass spec analysis should be provided in the main figures or tables. I acknowledge the access provided to mass spec data for this review, however it was unclear the format of data visualization for the manuscript.

The western blots for Figure 5A-C and 6E are not convincing. The p43/41 arrows are not well aligned, and the loading control in 5A is of poor quality with additional bands and cutoff - and not shown in supplemental. The authors should consider a positive control for cleaved Casp8 and Otulin if this cell line is not optimal, or consider further optimizing the western blots. The blot for SF5 needs a negative control to confirm that the cleavage is not a consequence of the protein prep method for neutrophils.

Can the authors comment on the disjointed ROS curves in Figure 6. What causes the large fluctuations in signal across the 1200s timecourse? Figure 6D requires a positive control – for the ALPS-CASP10 samples, do these cells have more general defects in generating ROS?

For CRISPR ko of HL60 cells, did the three clones from each KO respond in a similar manner? What data supports the knockout of these genes? Did the loss of Casp8 impact the growth or viability of CRISPR ko cells via RIPK1/RIPK3/MLKL?

Does DB550 have impacts on other aspects of the hematological, immune, and inflammatory response in the experiments illustrated in Figure 7? It seemed unusual for Figure 7 to be split across 3 pages so less clear if these will be an appropriate size if published.

Confirm if mouse models used 0.5ng/g or 0.5ug/g LPS.

Reviewer #3

(Remarks to the Author)
The manuscript presents an intriguing and potentially impactful study on the role of CD95/CD95L signaling in ANCA-associated vasculitis (AAV), with a focus on the contribution of soluble CD95L (sCD95L) to neutrophil activation and ROS production via caspase-dependent mechanisms. The identification of OTULIN as a novel caspase substrate and the involvement of LUBAC in mitochondrial ROS generation are particularly interesting observations that could have therapeutic implications.
However, I have major concerns regarding the mechanistic conclusions, particularly those involving caspase-10, which currently lack sufficient experimental support. In addition, the manuscript is missing methodological details, which must be addressed to support the manuscript's conclusions. These issues need to be thoroughly addressed before the manuscript can be considered for publication.
Major Concerns:
1. Caspase-10 involvement not clearly demonstrated
The manuscript claims that caspase-10 cleaves OTULIN, leading to LUBAC activation and mitochondrial ROS production in neutrophiles.
o Is there direct evidence of caspase-10 activation (e.g., cleavage, activity assays) in neutrophils treated with sCD95L?
2. Alternative caspases, particularly caspase-8, are not systematically ruled out. This is especially important given that:
• Both caspase-8 and caspase-10 are expressed in neutrophils.
• Caspase-10 is often present at lower concentrations in these cells compared to caspase-8.
• The manuscript does not clearly explain how the decision between caspase-8 and caspase-10 is regulated in this context.
• The use of specific inhibitors, knockdowns, or rescue approaches would be critical to establish caspase-10-caspase-8 crosstalk in these cells and to more convincingly show caspase-10 specificity in neutrophiles.
3. Specificity of Caspase-Mediated OTULIN Cleavage
The cleavage of OTULIN is attributed to caspase-10, but the possibility that other caspases (e.g., caspase-8 or -3) are involved is not adequately ruled out.
o Please clarify whether the cleavage sites identified are unique to caspase-10
4. Causal Link Between CD95/CD95L Signaling and ROS Remains Correlative
While the link between sCD95L and increased ROS in AAV neutrophils is well described, the pathway leading from CD95 engagement to mitochondrial ROS production via OTULIN cleavage is not convincingly demonstrated as a causative chain.
o Additional data are needed to establish this as a functional pathway rather than an association.
5. Recombinant Caspases: Missing Methodological Details
A major concern is the lack of a clear description of recombinant caspase preparation in the Materials and Methods section:
o The manuscript does not specify how recombinant caspases were purified, nor how their activity was verified or normalized across experiments. Specifically, this information is essential for the interpretation of the data in Figure 5
o Since conclusions regarding OTULIN cleavage rely heavily on these enzymes, this missing information is critical and must be included in the revised manuscript.
6. Figure 5G: Ubiquitination Data Not Quantitatively Supported
In Figure 5G, M1-linked ubiquitination is assessed using recombinant proteins, and the authors conclude that the Δ54 OTULIN mutant increases M1-ubiquitin levels by approximately 50%. However, this conclusion is not supported by the data, as:
o The Δ54 construct appears to be expressed at much higher levels than both WT and Δ31.
o No normalization is shown to account for differences in protein expression, making it unclear whether the observed increase is due to enhanced activity or simply overexpression.
o As such, the conclusion regarding the 50% increase in M1-ubiquitination is not experimentally justified and should be re-evaluated or removed unless properly controlled.
6. Limited OTULIN Cleavage in Patient Samples Challenges the Proposed Mechanism
The data presented in Figure 6 showing OTULIN cleavage in patient samples is valuable and adds clinical relevance to the study. However, a notable contradiction arises:

Only approximately 10% of OTULIN is cleaved, while 90% remains uncleaved in these samples.

This raises an important question about the threshold of OTULIN cleavage required to meaningfully impair its function and to trigger the downstream effects on LUBAC activity and ROS production.

It also suggests that OTULIN cleavage may not be the sole mechanism controlling ROS generation upon sCD95L treatment. Additional or complementary pathways may contribute, which should be acknowledged and explored.
7. Typographical and grammatical errors:
The manuscript contains language issues that should be addressed through proofreading.

Version 1:

Reviewer comments:

Reviewer #1

(Remarks to the Author)

The authors have provided a clear and thoughtful response to the reviewers' comments, and the revisions have strengthened the manuscript, particularly the inclusion of additional experiments in primary neutrophils. Overall, the authors have addressed my comments satisfactorily and I have only a few minor points:

1. The authors included a detailed analysis of patient characteristics and the association with sCD95L in the response to reviewers. While I appreciate this is not the main focus of the study it would be helpful to add a brief statement to the manuscript to highlight that within the limitations of the sample size no obvious clinical/confounding factors were identified to explain differences in sCD95L.
2. The mouse accelerated NTN study has been moved to the supplementary figures but the main text still contains a relatively detailed description of these data. As this experiment provides some additional information not assessed in the AAV models (e.g., neutrophil Ca^{2+} flux and ROS), it would be helpful to revise the text to more specifically highlight these unique aspects and reduce overlap with the AAV model description. As noted previously, I would also avoid referring to neutrophils from NTN mice as "AAV-like."
3. In Fig S6F please could you clarify if healthy neutrophils or NTN neutrophils are used?

Reviewer #2

(Remarks to the Author)
Concerns have been adequately addressed.

Reviewer #4

(Remarks to the Author)
The authors have responded to all of the three reviewers comments and improved the manuscript.
I was asked to assess the authors responses to the critiques by reviewer #3. These focus in great part on the role of caspase-10 and the level of OTULIN cleavage in patients.
I share this reviewers concerns and would like to specify them a bit more. My understanding is the author believe that the function of caspase-10 is replaced by caspase-8 in mice. That is also consistent with the dependency of ROS production in both human and mice on caspase-8, further supported by the fact that the pan-caspase inhibitor zVAD-fmk does not inhibit caspase-10. So, it is caspase-8 that is critical in both species. What is hard to understand is why caspase-8 would not cleave OTULIN D54 in humans if it is essential for the cleavage of mouse Otulin. Especially because the 14 amino acids around the D54 cleavage site are identical in human and mouse Otulin.
I appreciate the addition of the data on the cell-free system in Figure 5. The cell-free system provides an opportunity to figure out whether murine caspase-8 cleaves murine OTULIN which has to be predicted. That could be added and would address most of reviewer #3's concerns.

A few general remarks:
The abstract is not accessible to a nonspecialized reader. ANCA and LUBAC are not explained.
I do not understand why none of the figures presented to the reviewers in the rebuttal made it into the supplement? Some of these data need to be shown there.
There is a bit of a disconnect between the in vitro and the in vivo parts of the manuscript. The effects of the CD95 interacting peptide on the disease outcome in the different mouse models are not directly connected to the cleavage of Otulin by caspase-10 or in this case caspase-8.

Open Access This Peer Review File is licensed under a Creative Commons Attribution 4.0 International License, which permits use, sharing, adaptation, distribution and reproduction in any medium or format, as long as you give appropriate credit to the original author(s) and the source, provide a link to the Creative Commons license, and indicate if changes were made.
In cases where reviewers are anonymous, credit should be given to 'Anonymous Referee' and the source.
The images or other third party material in this Peer Review File are included in the article's Creative Commons license, unless indicated otherwise in a credit line to the material. If material is not included in the article's Creative Commons license and your intended use is not permitted by statutory regulation or exceeds the permitted use, you will need to obtain permission directly from the copyright holder.
To view a copy of this license, visit https://creativecommons.org/licenses/by/4.0/

To the referees,
We sincerely appreciate the reviewers' comments and constructive feedback and have made every effort to address all concerns thoroughly.
We believe that the substantial revisions, together with the additional experimental data, significantly strengthen the manuscript and clarify the mechanistic framework of our study.

RESPONSE TO REVIEWERS' COMMENTS

Reviewer #1 (Remarks to the Author):

In this study the authors investigate sCD95L induced neutrophil ROS generation, and identify that this may play a role in AAV. The study shows expression of CD95L on endothelial cells at tissue sites of vasculitis and identifies elevated serum levels in patients with AAV with highest levels in patients with active MPA-AAV. sCD95L induces ROS in neutrophils from patients with AAV or primed healthy donor neutrophils and this is shown to be dependent on mitochondrial calcium flux and caspase-8. Proteomics of CD95L-stimulated HL60 cells identifies OTULIN as a potential caspase substrate mediating this effect. **The authors then use a series of cell free experiments to dissect the molecular pathways showing OTULIN cleavage is likely due to caspase-10, with caspase-8 acting upstream.** Finally, inhibition of sCD95L is shown to be an effective treatment in mice with accelerated nephrotoxic nephritis, passive anti-MPO glomerulonephritis, and pulmonary vasculitis. **I thought the study was interesting, offering new insights into endothelial-neutrophil interactions in AAV. The mechanistic studies were comprehensive, dissecting out various elements of the caspase pathways.**

Major Comments

1. My main criticism is that the range of different stimulation methods/cell types used sometimes made the study difficult to follow. Experiments were variously carried out using AAV serum stimulation of healthy neutrophils, sCD95L stimulation of AAV neutrophils and primed healthy neutrophils, and sCD95L stimulation of HL60 cells. Different mechanistic elements are then carried out in some but not all of the different experimental set ups. I would suggest restructuring to focus on one experimental set up at a time. **In particular a smaller number of experiments are carried out using the AAV serum stimulations so focussing more on AAV neutrophils/primed healthy neutrophils for identification of mechanisms would be one option.**

We acknowledge that the use of different stimulation methods and cell types in the original manuscript may have affected the clarity and flow of the study. In the revised version, we have reorganized the figures and focused primarily on experiments using IL-1β or TNFα-primed neutrophils from healthy donors and neutrophils from AAV patients (**Figures 2–4**).

Specifically, we used primary neutrophils from healthy donors or AAV (MPA) patients to investigate the role of CD95 and CD95L (using DB550 and the neutralizing anti-CD95L mAb NOK1, **Figures 2C–3B**), caspases (zVAD, zIETD, zDEVD, zAEVD, **Figure 4 and Figure 6D**), calcium signaling (BAPTA-AM, **Figure S2D**), mitochondrial function (MitoTempo, L14/15, ABT263, **Figures S2E–F**), and linear ubiquitin ligase activity of HOIP (HOIPIN-1, **Figure 6A**) in CD95-mediated ROS production. Additionally, cleavage and expression of Otulin were examined in neutrophils from AAV patients (**Figure 6F**). To further strengthen the mechanistic findings, we have included new data showing that Otulin and caspase-10 are cleaved upon sCD95L stimulation of primary neutrophils (**New Figure 5F**).

We note that while performing all experiments in primary neutrophils would be ideal, their short half-life and non-dividing nature precluded genetic studies[1]. To overcome this, we used the neutrophil-like HL60 cell line to investigate the biological roles of key mechanistic elements, including Otulin, caspase-8, and caspase-10, in CD95-mediated signaling (**Figure 5, except 5F**). As shown in **Figure**

S3, HL60 cells respond to CD95 engagement similarly to primary neutrophils, supporting the relevance of these findings.

2. The time point of experiments and short neutrophil lifespan is given as justification for using HL-60 cells over primary neutrophils- however even for the proteomic study only a 30 minute incubation period was used which is well within a neutrophils in vitro lifespan. The HL60 cells behave differently to healthy primary cells and respond to sCD95L to a greater degree than primary neutrophils and constitutively cleave otulin. I would suggest where possible, other than experiments using Crispr KO which I appreciate may be technically challenging in primary cells, experiments should be repeated in primary cells (**such as Fig 5A**). For any studies which it is not possible to repeat (such as the proteomics) this should be discussed as a limitation.

As mentioned above, most pharmacologic experiments have been done with either primary neutrophils isolated from healthy donors pre-stimulated with TNFα or IL1β or with neutrophils isolated from patients with AAV. In contrast, the genetic experiments with CRISPR/Cas9 extinction of caspase-10, caspase-8, caspase-3 and Otulin have been performed using the neutrophil-like HL60 cell line (**Figure 6B-C**).
We agree with the referee that the time point chosen for the proteomic analyses does not justify the use of HL60 cells instead of purified neutrophils from healthy donors and this explanation has been removed from the revised version. We selected HL60 cell line because **i)** it recapitulates the whole signaling pathway observed in AAV neutrophils and primary neutrophils pre-activated with IL1β or TNFα (see manuscript **Figure S3**), ii) it is more resistant to experimental manipulations as compared to fresh neutrophils and iii) importantly, the use of a cell line, eliminates inter-donor or inter-patient variability, which would have complicated the interpretation of the proteomic data (**discussed page 11 of the revised manuscript**).
Moreover, we have added a sentence in the discussion section to address the limitations of using the HL-60 cell line (**page 26 of the revised manuscript**).
We realized additional experiments using primary neutrophils. Specifically, we show in the new **Figure 5F**, that Otulin and caspase 10 are cleaved in neutrophils isolated from healthy donors and exposed to sCD95L.

3. ANCA IgG is well described to induce ROS so it is interesting that in the serum stimulation experiments blocking sCD95L results in almost complete ROS inhibition. It would be useful to show what the effect on ROS generation is if IgG is depleted from the serum. Similarly, what were the sCD95L levels in the specific sera used for these studies?
And which cohort of patients do they represent; are they from patients with both MPA and GPA and what was their disease activity?

To assess the role of IgG in the sera of AAV patients in neutrophil ROS production, sera from 2 different MPA patients were depleted for IgG and tested for ROS production on IL1β-stimulated healthy donor neutrophils (**Rebuttal letter Figure 1**). The elimination of IgG in these sera did not affect their ability to induce ROS production in neutrophils suggesting that this ROS production occurred in an IgG independent fashion.

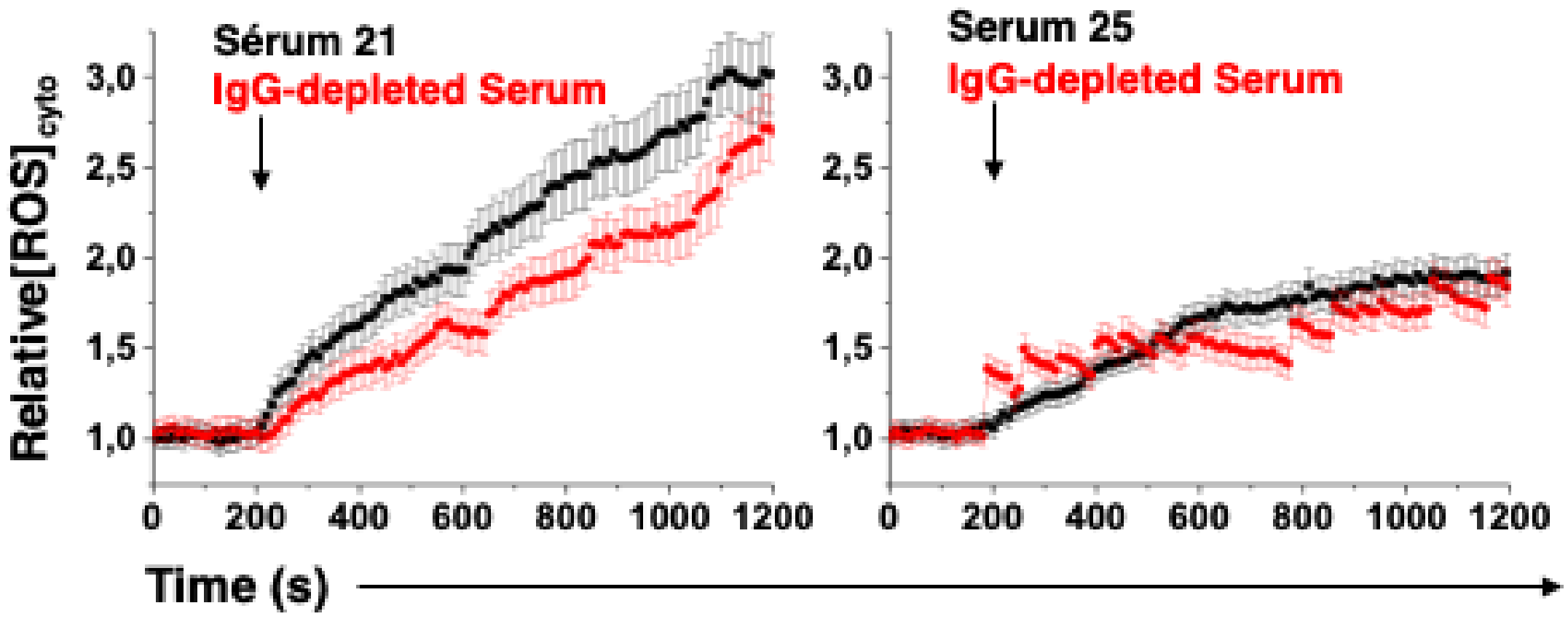

Rebuttal letter figure 1: Effect of IgG depletion in sera from patients with AAV (MPA) on neutrophil ROS production. *The IgG depletion in sera from patients with AAV does not affect the ROS production in neutrophils isolated from healthy donors. Briefly, healthy donor neutrophils were exposed to indicated sera (dilution 1/1000) and the cytosolic ROS was monitored. The concentration of soluble CD95L in MPA sera was dosed by ELISA (serum21: 218 pg/mL and serum 25: 295 pg/mL).*

Method for IgG depletion: depletion of IgG in AAV patients was performed using the NAb™ Protein A Plus Spin Column Kit (#89956, Thermo Fisher Scientific) according to the manufacturer's instructions. Briefly, Protein A columns were equilibrated with binding buffer (0.1M phosphate, 0.15M sodium chloride; pH 7.2), diluted serum was applied to the columns, and samples were incubated for 10 min at room temperature. After centrifugation to remove unbound proteins, columns were washed twice with binding buffer, and bound IgG was eluted with elution buffer (0.1M glycine; pH 2-3). Eluates were immediately neutralized with 1 M Tris-HCl (pH 9.0). IgG concentrations were determined by spectrophotometry at 280 nm.
To validate the depletion procedure, IgG-depleted serum fractions and total serum were quantified for IgG levels using immunonephelometry on a BN2 analyzer (Siemens Healthineers). Serum 21 contained 23.2 mg/mL of IgG prior to depletion and 0.42 mg/mL after depletion. Serum 25 contained 14.8 mg/mL of IgG before depletion and 0.44 mg/mL after depletion.

Because our data indicated that patients with MPA showed a significantly higher concentration of sCD95L during active disease compared to non-active **(Figure 1C)** and because sCD95L concentration decreases in the sera of MPA patients treated with immunosuppressive drugs (**Figures 1D**), the remainder of the study focused on patients affected by MPA. This has now been clarified in the main text of the manuscript (**page 8 of the revised manuscript**).

4. The elevated levels of sCD95L in active MPA compared to GPA is interesting but not discussed further. Can the authors offer a hypothesis as to why this might be the case?

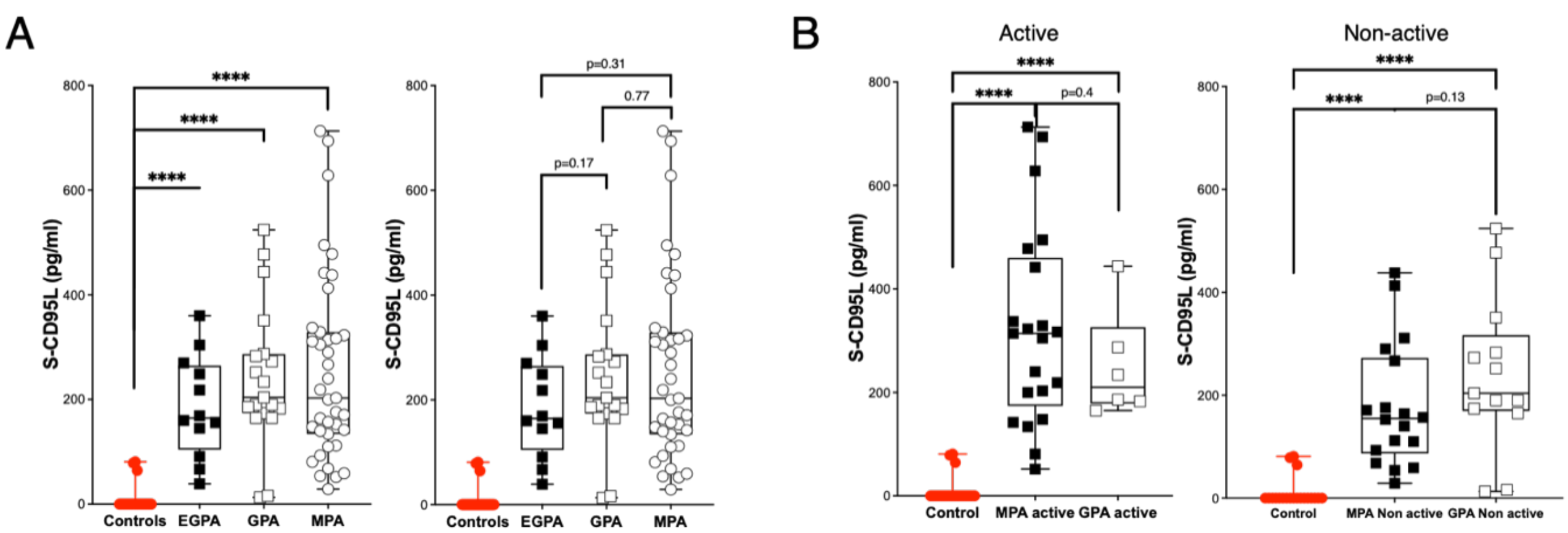

Rebuttal letter figure 2: Different representations of the manuscript Figures 1B and 1C. *Soluble CD95L was dosed by ELISA in sera of the indicated patients. **** p<0.0001, using two-tailed*

Mann-Whitney test. ***A. left panel:*** *Statistical analyses performed between control donors and AAV patients.* ***Right panel:*** *Statistical analyses performed between sub-groups of AAV patients.* ***B. left panel:*** *Statistical analyses performed between active AAV and GPA patients.* ***Right panel:*** *Statistical analyses performed between non-active AAV and GPA patients.*

As shown in **rebuttal letter Figure 2A (left panel)**, although the concentration of sCD95L was increased in sera of patients with EGPA, GPA and MPA compared to healthy donors, we did not observe any significant difference in the concentration of serum sCD95L between MPA, GPA and EGPA patients. We performed statistical analyses on sCD95L levels dosed in active (**rebuttal letter Figure 2B**, ***left panel***) and non-active (**rebuttal letter Figure 2B**, ***right panel***) patients with MPA and GPA. The concentration of sCD95L was not significantly increased in active MPA compared to active GPA patients (**rebuttal letter Figure 2B**, ***left panel***). Similarly, no significant differences were observed between inactive patients affected with MPA and GPA.
Because our statistical analyses revealed that a significant difference existed between active and non-active MPA patients, the rest of the study has been realized using MPA sera and neutrophils isolated from MPA patients.

Why active MPA patients exhibit significantly higher levels of sCD95L compared to inactive patients, whereas active GPA patients show similar levels to inactive patients? We did not yet explore this question but this difference could be attributed to i) the small sample size in the active GPA group or ii) to clinical (a) or pathophysiological distinctions (b) between GPA and MPA.

Indeed, (a) MPA patients were older (66.2 vs. 57.5 years, p = 0.0074) and presented more renal involvement (97.4% vs. 73.7%, p = 0.0119) compared to GPA patients. Additionally, the MPA group included significantly more severe patients (41% *vs*. 10.5%, p = 0.0322) (European vasculitis Society, (EUVAS) criteria). Conversely, GPA patients exhibited higher frequencies of neurological (47.4% *vs*. 15.4%, p = 0.0226), joint (57.9% *vs*. 28.2%, p = 0.0436), ENT (68.4% *vs*. 33.3%, p = 0.0231), and ophthalmological involvement (21.1% *vs*. 0%, p = 0.0091). Finally, GPA patients received more Trimethoprim/sulfamethoxazole (57.9% vs. 25.6%, p = 0.0221) and Methotrexate (21.1% *vs*. 2.6%, p = 0.0355), possibly due to the more frequent joint involvement in this group. Finally, iii) this difference in the treatments proposed to MPA and GPA patients might modulate either the magnitude of expression or cleavage of CD95L and thereby, affect the difference of sCD95L concentration between MPA and GPA patients. iv) Beyond these clinical differences, MPA and GPA may also differ in their pathophysiological mechanisms. For instance, MPA might rely more heavily on neutrophils than GPA. Mechanisms such as NETosis and cell death, as well as the kinetics between disease onset and the rise in sCD95L levels, could vary between the two conditions. Inactive GPA patients might maintain higher levels of sCD95L compared to MPA (even if this difference of sCD95L levels between these patients is not significant, see **rebuttal letter Figure 2B**, ***right panel)*** due to persistent granulomatous inflammation and chronically activated T lymphocytes and macrophages, which may continue to express CD95L. These explanations remain as hypotheses.

This association should also be corrected for confounders such as renal function, disease severity, or inflammatory state. It would also be useful to carry out multivariable analysis for the association with BVAS. **Are data available on eGFR or CRP?**

As requested by the reviewer, we performed analyses of the sCD95L concentration with different clinical factors and **we did not observe any evident confounding factors that could explain the elevation of sCD95L in MPA patients.** Specifically, no correlations were found with renal function (creatinine, eGFR calculated by MDRD), proteinuria, Berden score, disease severity (BVAS), or inflammatory status (CRP), as illustrated in the **rebuttal letter figure 3**.

Although the limited sample size (n=20) precluded multivariate analysis, no individual correlations were observed for relevant parameters, particularly eGFR or CRP. Collectively, these data support the conclusion that sCD95L elevation in AAV is specific and not attributable to any identified confounding factor.

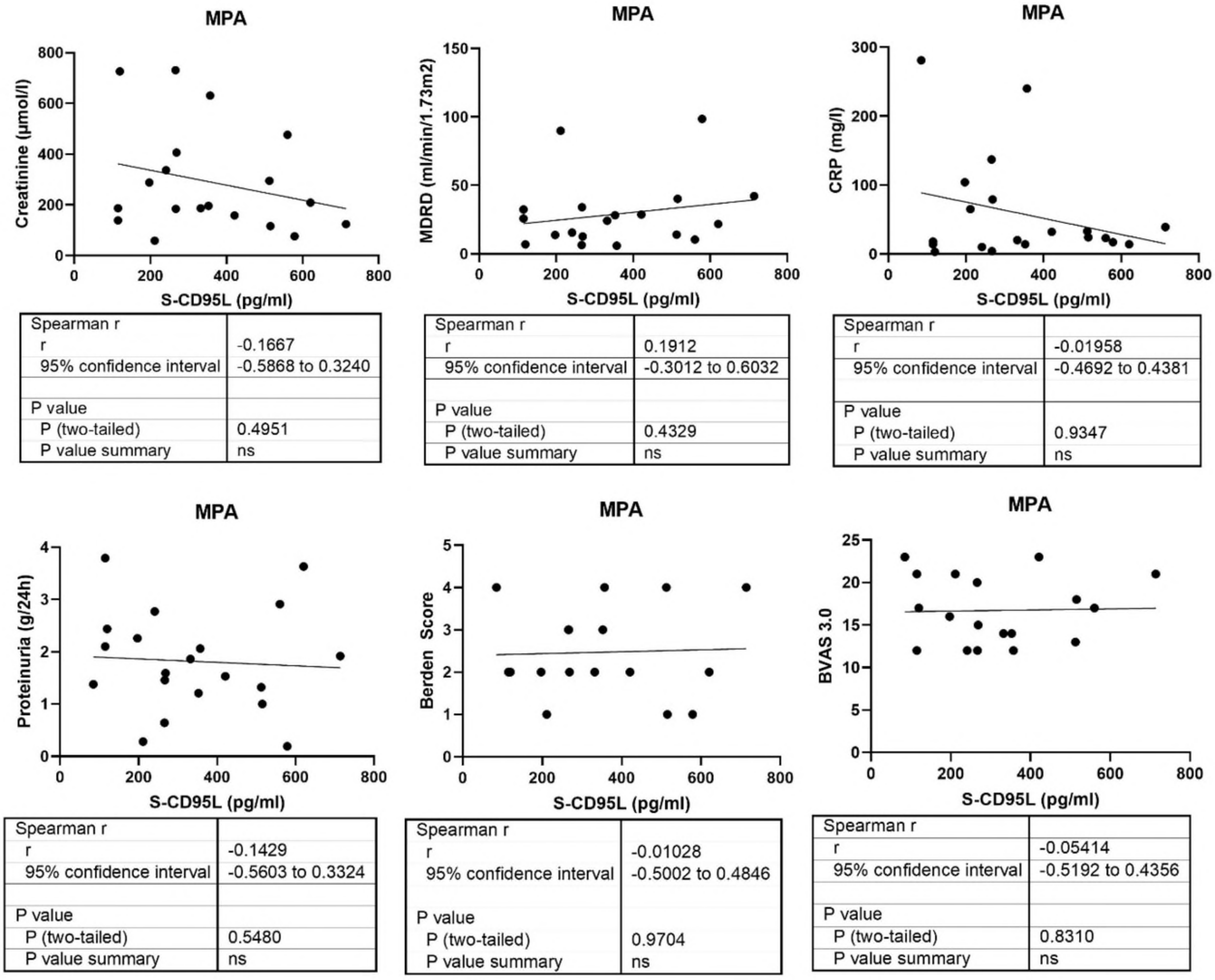

Rebuttal letter figure 3: Correlation between sCD95L concentration and different clinical factors.

5. Some studies have identified that AAV neutrophils produce less ROS than those from healthy donors (PMID: 37947315, 27102815). One study also suggests that MPA neutrophils generate less ROS than GPA patients (albeit in response to non physiological stimulus with PMA) which seems contrary to the higher levels of sCD95L in MPA. It would be useful to include some discussion on this.
Were the AAV neutrophils used in the study from patients with both MPA and GPA?

As the reviewer correctly pointed out**,** studies have reported that AAV neutrophils produce less ROS than those from healthy donors**.**
Nonetheless, all mentioned studies focused on <u>the phagocytosis process,</u> with a reduced ROS production attributed to <u>a defect in NADPH oxidase–driven ROS generation</u>. This impaired NADPH oxidase activity has been associated with increased AAV severity[2].
Importantly, in PMID: 37947315, neutrophils were isolated from GPA patients, whereas our study focusses on MPA patients, an aspect we now emphasize in the revised manuscript. Of note, this latter study did not evaluate the mitochondrion-induced ROS production.
Similarly, in PMID: 27102815, most patients were in remission (70%) and the authors evaluated ROS production in neutrophils from AAV patients exposed to PMA (a protein kinase C activator), or E. coli. Both stimuli activate the NADPH-oxidase-driven ROS production. To study ROS production, the authors used the IVD-labeled PhagoBurst assay, (Glycotope Biotechnology, GmBH, Germany), which determines the percentage of phagocytic cells producing reactive oxidants. This ROS production during phagocytosis mainly relies on NADPH-ox activity[3].

In summary, these methodologic differences might explain why the reduced NADPH-ox-dependent ROS production impairs phagocytosis in ANCA-vasculitis neutrophils, while sCD95L triggered a stronger ROS production in these cells as compared to neutrophils isolated from healthy donors (**Figure 2D**) through a mitochondrion-dependent mechanism (**Figure S2E and S2F**).
We did not evaluate the NADPH-oxydase activity, mainly localized at the plasma membrane[4], since our data clearly show that the CD95-mediated ROS production in neutrophils relied on the mitochondria **(Figure S2E-F**).
Overall, the referee's comment is not in contradiction with our data: although the NAPDH-oxydase-dependent ROS production could be impaired in AAV neutrophils and thereby, affecting the phagocytosis, our data reveal that sCD95L induced a stronger mitochondrial-driven ROS production in AAV neutrophils than in neutrophils from healthy donors, through a caspase-10-Otulin signaling pathway.
We now discussed this point in the discussion of the manuscript (**page 26 of the revised manuscript**).

As previously mentioned, the remainder of the study was conducted using sera and neutrophils isolated from MPA patients**.** This aspect is now clarified in the revised version of the manuscript (**page 8 of the revised manuscript**).

6. Figure 6- What effect does caspase or otulin ko have on calcium flux or cytosolic ROS? Similarly when the range of caspase inhibitors are used in primed healthy neutrophils what impact does that have on mitoROS/calcium?

Without CD95L stimulation, we did not observe any effect on basal ROS or calcium level in otulin and caspase-3, caspase-8 and caspase-10-ko HL60 cells compared with wild-type cells.
Similarly, the treatments with caspase-8 inhibitor zIETD and the pan-caspase inhibitor zVAD-fmk did not cause major effects in the Ca^{2+} and ROS responses neither in AAV neutrophils (**Figures 4A-B**) nor in TNFα or IL1β-pretreated neutrophils from healthy donors (**Figure 4C**)

7. Accelerated nephrotoxic nephritis is a model of immune complex GN using a planted alloantigen and does not represent either anti-GBM disease or AAV. In particular it is not pauci-immune and the histopathological lesion has several differences to AAV in humans. I would avoid referring to it as a model of AAV and would suggest referring to the model as either NTN or crescentic GN. As the authors have used two other models in mice which are more representative of AAV I would suggest moving the NTN experiment to the supplemental figures.

We thank the reviewer and fully agree with the comment. The figure corresponding to this crescentic GN animal model has been moved to the Supporting Figures (**Figure S6 of the revised manuscript**).

8. Are serum CD95L levels raised in either of the vasculitis models studied?

As requested, dosage of sCD95L has been performed in the BAL or sera in the two AAV animal models. These data have been included in the revised version of the manuscript (**New Figure 7B and 7I**).

Minor comments

1. Fig 1A- an image of a glomerulus in HC tissue should be included rather than just tubulointerstitium

We have updated the figures to include images of the glomeruli (**Figure 1 and S1 of the revised manuscript**).

2. Is there any significant neutrophil infiltrate in the passive AAV GN model?

As shown in the newly added **Figure S6J**, we quantified neutrophil infiltration in the kidneys of the passive AAV GN model. Neutrophil infiltration was increased in the passive AAV GN model compared with healthy control mice and was reduced following DB550 treatment.

Reviewer #2 (Remarks to the Author):

The manuscript by Boizard-Moracchini and colleagues examines the effects of soluble CD95L on Casp10-driven ROS in neutrophils and the relationship to ANCA. **The manuscript is of interest for understanding disease progression but lacks key controls**. The introduction should provide better context for why these findings are not incremental when compared to published studies addressing roles for FasL and neutrophils in ANCA.

We agree that the Introduction required clearer positioning relative to prior studies on CD95L and neutrophils in ANCA. We have therefore revised the Introduction to explicitly distinguish our findings from earlier work.

Previous studies established that metalloprotease-cleaved CD95L promotes neutrophil migration[5], whereas multimeric CD95L induces apoptosis via mitochondrial pathways[6]. In contrast, our study identifies a **previously unrecognized signaling axis** in which soluble CD95L selectively activates **caspase-10**, leading to **OTULIN cleavage**, **LUBAC activation**, and **mitochondrion-dependent ROS production** in neutrophils. Given that excessive ROS generation by neutrophils contributes to the pathogenesis of AAV[7], we investigated the role of soluble CD95L (sCD95L) in this disease and demonstrated that inhibition of this pathway markedly ameliorates clinical symptoms in three distinct AAV-like mouse models. To our knowledge, the functional link between CD95L, caspase-10, and OTULIN has not been reported and cannot be inferred from existing literature. Moreover, the role of soluble CD95L in MPA/AAV pathogenesis had not been previously suggested.

These original findings clearly represent a significant advance compared to earlier studies on CD95-induced non-apoptotic signals [8, 9, 10, 11, 12, 13, 14, 15, 16].

The Introduction has been rewritten accordingly (**page 6 of the revised manuscript**).

Figure 1A requires a higher magnification image together with quantitation for a measure of colocalization between CD31 and CD95L. What other cells could also be present in these areas to also express CD95L? Any CD45+? It appears likely it is endothelial but at this magnification it is difficult to be confident in this disease setting.

We have revised **Figures 1A and S1** to include higher-magnification images and glomerular regions, improving visualization of CD95L expression in AAV kidneys. While immunofluorescence cannot formally exclude expression by additional cell types, CD95L signal strongly co-localizes with the endothelial marker CD31 in renal tissue. Importantly, endothelial co-localization is unequivocally demonstrated in the skin of AAV patients (**Figure S1B**), supporting an endothelial origin of CD95L in inflamed tissues.

Figure 2E. The difference in ROS production may be explained by differences in neutrophil activation from healthy controls or AAV patients. **A phenotypic characterization of these neutrophils is required to account for these likely differences.**

As requested, we performed a detailed phenotypic characterization of neutrophils from healthy donors, AAV (MPA) patients, and IL-1β–primed healthy neutrophils using multiparameter flow cytometry (**Rebutal letter Figure 4**).

Considering that AAV neutrophils are activated in an inflammatory environment, where multiple stimuli are present, it remains difficult to compare these cells with the IL1β-primed neutrophils. Nonetheless, we analyzed different activation markers including CD66b and CD63 (markers of degranulation[17]) and CD62L (marker associated with endothelial transmigration[18]) on these neutrophil sub-populations. As expected, we observed that the AAV neutrophils displayed an

activated phenotype, with an up-regulation of CD63 and a trend for CD66b suggesting degranulation (**rebuttal letter Figure 4B**). On the other side, CD62L was not down-regulated (**rebuttal letter Figure 4B**). In contrast, IL-1β priming did not alter these activation markers, even after 3 hours of incubation (**rebuttal letter Figure 4B**).
Although IL-1β and/or TNF-α do not fully reproduce the exact phenotype observed in patients, these cytokines are sufficient to sensitize normal neutrophils to the CD95L/CD95 pathway to a similar extent as that observed in AAV-derived neutrophils.

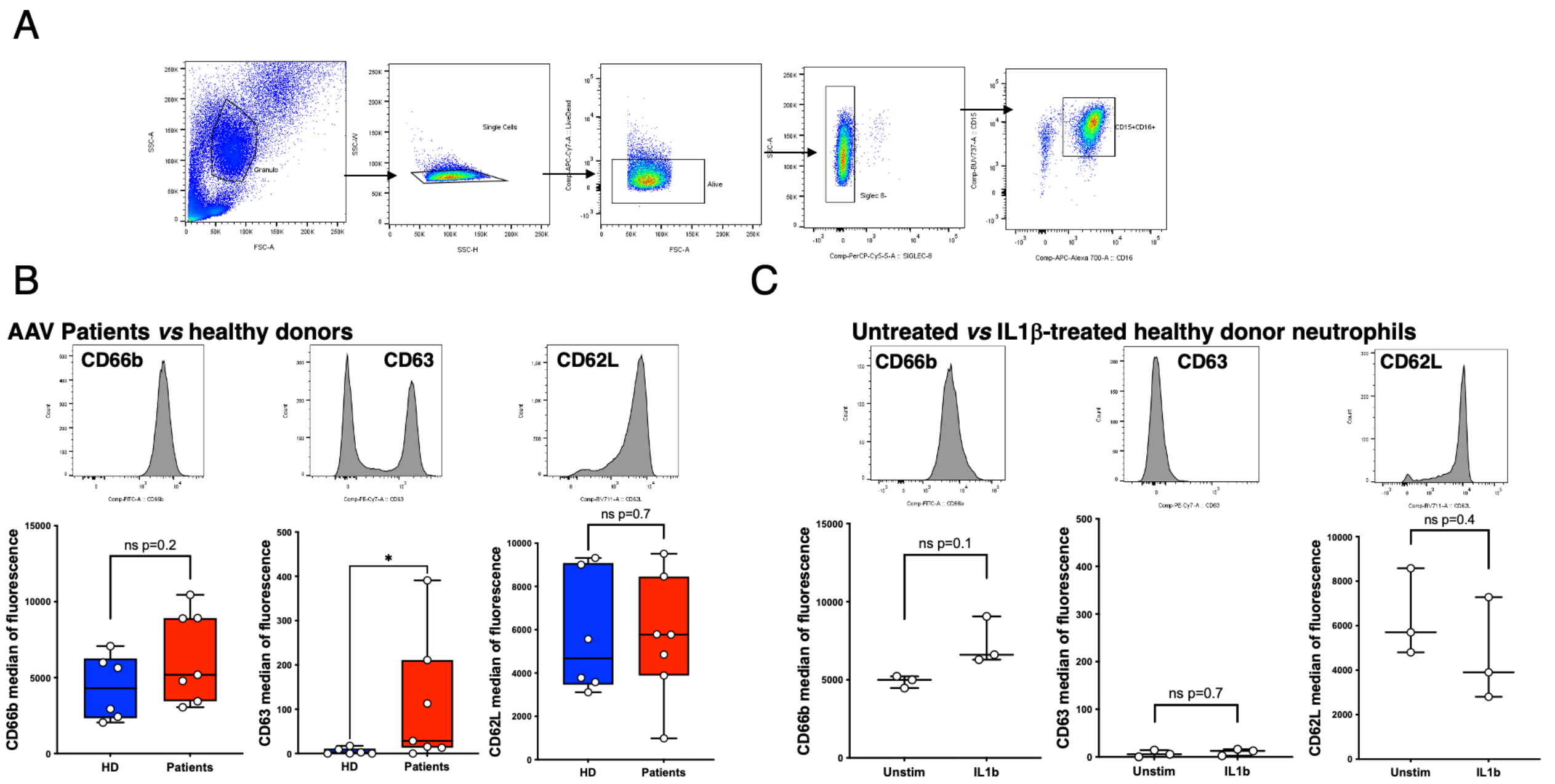

Rebutal letter Figure 4: Neutrophils isolated from patients with AAV are activated. A. Gating strategy for analysis of the activation status of neutrophils isolated from healthy donors or AAV patients. **B-C.** Analysis of the expression level of different markers of neutrophil activation (CD66b and CD63 up-regulated during degranulation and CD62L downregulated when endothelial transmigration/adhesion is increased). **B.** ***Upper panels.*** Neutrophils were isolated from whole blood of healthy donors or AAV patients (MPA) and the flow cytometry histograms of the indicated markers were depicted. ***Lower panels.*** Statistical analyses of median intensity of fluorescence for the neutrophils isolated from AAV patients or healthy donors (n=7 AAV patients and n=6 healthy donors). **C.** Neutrophils were isolated from whole blood of healthy donors and pre-incubated for 3 hours in the presence or absence of IL1β (100 ng/mL). ***Upper panels.*** The flow cytometry histograms of the indicated markers were depicted. ***Lower panels.*** Statistical analyses of median of fluorescence for the indicated cells isolated from healthy donors (n=3).

Method:

- For the analyses of AAV neutrophils, blood samples from healthy donors (n=6) or AAV patients (n=7) were processed for flow cytometric analyses. Briefly, 500 µL of whole blood was used per condition. Red blood cells were lysed using ACK lysis buffer for 10 minutes at room temperature. Lysis was stopped by diluting the ACK lysis buffer with FACS buffer (PBS supplemented with BSA and EDTA), followed by centrifugation and washing. Cells were then stained with a viability dye eFluor 780 (65-0865-14, eBioscience) for 10 minutes at room temperature in the dark, washed, and subsequently incubated with fluorochrome-conjugated monoclonal antibodies against CD63, CD66b, CD15, Siglec-8, CD16, and CD62L (all antibodies were purchased from BD Bioscience, see table below) for 15 minutes at room temperature in the dark. After staining, cells were washed and resuspended in 500 µL of FACS buffer. Data acquisition was performed on a BD LSRFortessa X-20 flow cytometer (BD Biosciences). Data were analyzed using FlowJo software (BD Biosciences). Neutrophils were identified using a sequential gating strategy based on forward and side scatter properties, followed by doublet exclusion and exclusion of dead cells using a fixable viability dye. Eosinophils were excluded based on Siglec-8 expression, and neutrophils were defined as $CD15^{+}$

CD16+ cells. Expression of CD66b, CD62L, and CD63 was subsequently analyzed within the neutrophil gate (see **rebuttal letter Fig.4A**).

- For IL1β-stimulated neutrophils, cells were purified from peripheral blood of healthy donors obtained from the Établissement Français du Sang (EFS), as described in the Materials and Methods section in the revised manuscript. Purified neutrophils were seeded at a density of 50,000 cells per well in flat-bottom plates and cultured in the presence or absence of recombinant human IL-1β (100 ng/mL). After 3 hours of incubation, neutrophils were collected and stained with the same fixable viability dye and fluorochrome-conjugated antibodies used for patient samples, including CD63, CD66b, CD15, Siglec-8, CD16, and CD62L. Data acquisition was performed on a BD LSRFortessa X-20 flow cytometer (BD Biosciences), and data were analyzed using FlowJo software (BD Biosciences), as described above.

Antigen	Fluorochrome	Clone	Cat. ref
CD63	PE Cy7	H5C6	561982
CD66b	BB515	G10F5	564679
CD15	BUV737	W6D3	741876
Siglec-8	RB705	837535	757374
CD16	Alexa Fluor 700	3G8	560713
CD62L	BV711	SK11	565039

Of note, basal ROS and calcium signals were normalized to 1 for each cell to account for inter-donor variability and probe loading efficiency. This approach selectively evaluates stimulus-induced changes and is now explicitly justified in the Methods.

More specifically, comparing basal levels of ROS remains very difficult to realize since the intensity of this signal depends on the efficiency of ROS probe loading for each patient or subject. Therefore, although we did not observe a major difference between resting and primed neutrophils or between neutrophils isolated from healthy donor and AAV patients, we cannot exclude that the activation status of neutrophils might slightly modulate these basal ROS concentrations prior to sCD95L stimulation. Nonetheless, our method of analysis normalizes these basal signals to a similar value in order to selectively evaluate the magnitude of the sCD95L signal in these cells.

In summary, comparing the basal concentrations of ROS in cells isolated from different subjects is extremely challenging, and should be performed with ratiometric probes. No ratiometric probe exists for ROS measurement. Therefore, for each treatment, the basal level of calcium or ROS was set to 1 and the probe's fluorescence intensity reflects the magnitude of changes in Calcium or ROS concentration in stimulated cells.

It is noteworthy that in **Figures 2A, 2B and 2C**, the different ROS curves represent neutrophils isolated from the same healthy donor and stimulated with different sera, ruling out any differences in the activation status of the neutrophils for these experiments.
Figure 2D shows an increase in the CD95-mediated ROS production in neutrophils isolated from patients with AAV as compared to the signal measured in healthy donor neutrophils, supporting that the AAV neutrophils were over-activated compared to neutrophils isolated from healthy donors.
By testing different cytokines and complement factors, we found that this activation state was recapitulated in healthy donor neutrophils by pre-incubating the cells with either IL-1β and/or TNF-α (**manuscript Figures 2E and 3D**). These findings highlight that a cytokine cocktail encompassing IL1β, TNFα and sCD95L contributes to the tissue damage in patients with AAV, a point is discussed in the manuscript.

The Figure legends (for S2 as an example) imply the mean +/- SD of 41 biological replicates is shown. Were these performed on different days? Or are these technical replicates from a smaller number of biological replicates? The majority of graphs plot 'relative ROS', for example, so how is the relative signal compared to other positive controls that are known to generate high levels of ROS in this assay?

The graphs represent mean ± SD of individual single-cell traces (n = 41), derived from at least three independently performed experiments or donors/patients, as now clarified in the Methods (**Main text, page 40**) and the figure legends. Because no ratiometric ROS probe exists, signals are expressed relative to baseline. This normalization strategy is standard for single-cell ROS and calcium analyses and was applied consistently across experiments.

The authors state that the proteomic assays was performed in HL-60 cells because primary neutrophils have a short half-life, despite conducting the majority of experiments in primary neutrophils. While neutrophils can be maintained in culture for >24h with pro-survival cytokines, the proteomics assay was only performed for 30 min, so was the justification more centered around the challenges of executing proteomics on primary neutrophils rather than life span?

We agree with the reviewer's point and have revised the rationale accordingly. Although neutrophils can survive short-term culture, performing reproducible, multi-replicate proteomics on primary neutrophils is technically challenging due to donor variability and limited material. Because neutrophil-like HL60 cells faithfully recapitulate sCD95L-induced calcium and ROS signaling (**Figure S3**), we used this model to perform five independent proteomic analyses with improved robustness. The manuscript text has been revised to reflect this rationale (**page 11 of the revised manuscript**).

Figure 4F is uninterpretable at this size. The text also refers to this as a cell death assay where the homemade dodecameric IgCD95L 'induced a strong caspase-dependent cell death signal'. The assay is only performed for 30 min so authors should provide biochemical and cellular evidence that this indeed distinguishes apoptotic and non-apoptotic cell death. It is already established in the literature that the strength of FasL signal on neutrophils determines the kinetics of cell death and the proportion of the population entering into apoptosis. Are the effects in Figure 4F and elsewhere a consequence of kinetic changes in the neutrophil population? These are important considerations when comparing different types of FasL, a potent inducer of human neutrophil apoptosis.

The proteomics in Figure S4 refers to the appearance of 2 peptides. This is a non-standard depiction of mass spec data. Additional description of the results of the mass spec analysis should be provided in the main figures or tables. I acknowledge the access provided to mass spec data for this review, however it was unclear the format of data visualization for the manuscript.

We agree that the original **Figure 4F** was not essential to the main message and we have therefore **removed this figure**.

As mentioned by the referee, a putative apoptotic signal induced by sCD95L and/or IgCD95L on HL60 cells could affect our proteomic analysis. To tackle this question, we have assessed apoptosis on DMSO-differentiated neutrophil-like HL60 cells exposed to IgCD95L or sCD95L at different time points ***(rebuttal letter figure 5).*** Overall, these data demonstrate that neither ligand induces mitochondrial depolarization or apoptosis within the 30–60 min time frame used for proteomics and for the other assays shown in this study.

The multi-aggregated and apoptotic ligand, IgCD95L[19] induces apoptosis only at later time points (24 h). This confirms that the proteomic signatures reflect caspase-mediated signaling rather than cell death–associated degradation (**rebuttal letter Figure 5**).

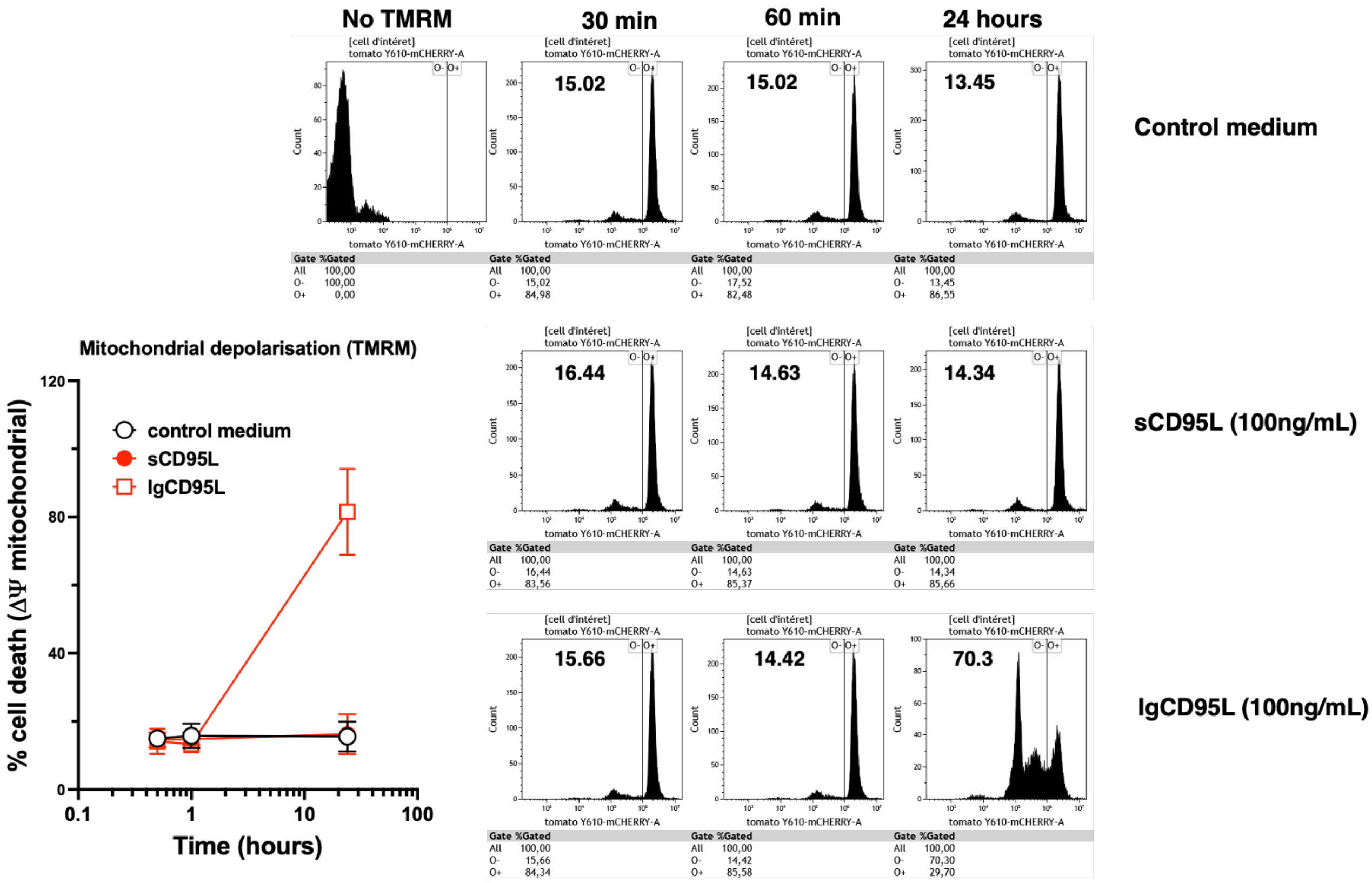

Rebuttal letter figure 5: HL60 cells exposed to IgCD95L or sCD95L do not undergo apoptosis at 30 min.
To measure the early steps of apoptosis in HL60 cells exposed to sCD95L or IgCD95L, we performed a mitochondrial depolarization assay. Indeed, mitochondrial depolarization is a precocious marker of cells death. DMSO-differentiated HL60 cells were pre-incubated for 30 min with TMRM (40 nM) and next stimulated with or without 100 ng/mL of IgCD95L or sCD95L for the indicated time points. Mitochondrial depolarization (ΔΨ) was assessed by flow cytometry. IgCD95L, the cytotoxic ligand only induces apoptosis in DMSO-treated HL60 cells at 24 hours. No cell death was measured up to 1 hour after stimulation with the different ligands.

To monitor the early steps of the CD95-mediated apoptotic signal, we used the mitochondrion-selective probe tetramethylrhodamine methyl ester (TMRM), which accumulates in living and polarized mitochondria and is released upon depolarization in dying cells [20]. After CD95 engagement, the depolarization of the inner mitochondrial membrane electrochemical gradient (Δψm) is **a robust and early marker of apoptosis**, as it is required for the release of apoptogenic molecules from the mitochondria.
We confirmed that up to 1 hour, no drop of Δψm was measured in neutrophils exposed to sCD95L (100ng/mL) or IgCD95L (100ng/mL) (**rebuttal letter Figure 5**). Only IgCD95L can trigger apoptosis in HL60 cells after 24 hours (**rebuttal letter Figure 5**).

Overall, these data pointed out that i) the caspase activation induced by both pro-inflammatory (sCD95L) and the apoptotic (IgCD95L) signals converges to the cleavage of Otulin at D54 and ii) these molecular mechanisms involved in the induction of a mitochondrion-dependent ROS production might contribute to two distinct cellular outcomes (*i.e.,* inflammation and cell death) in activated neutrophils.

As requested, a table (**Table 4**) has been added to complete the manuscript **Figure S4A** and explain how common peptides to sCD95L and IgCD95L stimulations have been selected. The main text of the manuscript has been changed to explain the strategy we used to identify the peptides lost in HL60 cells exposed to sCD95L and IgCD95L and restored when zVAD-fmk was added (**Main text pages 12 and 13**).

The western blots for Figure 5A-C and 6E are not convincing. The p43/41 arrows are not well aligned, and the loading control in 5A is of poor quality with additional bands and cutoff - and not shown in supplemental. The authors should consider a positive control for cleaved Casp8 and Otulin if this cell line is not optimal, or consider further optimizing the western blots.
The blot for Fig.S5 needs a negative control to confirm that the cleavage is not a consequence of the protein prep method for neutrophils.

It is well known that neutrophils contain granules enriched in proteases, which makes it challenging to obtain immunoblots comparable to those typically generated from T or B cells. In the revised manuscript, **Figure 5F** was generated using an alternative method that allows gentle lysis of neutrophils without disrupting their protease-containing granules. This new protocol is described in the Materials and Methods section (**updated on page 44**) and does not alter the results obtained with the previous method. It slightly improves the overall quality of our immunoblots; however, it does not enhance the quality of the caspase-8 immunoblot.
As requested by the referee, sCD95L kinetics were assessed in both DMSO-differentiated HL-60 cells and control or IL-1β–primed neutrophils isolated from healthy donors (**new Figure 5F**). We confirmed that sCD95L induced caspase-10 activation and OTULIN cleavage in these cells. Interestingly, caspase-10 activation—monitored by the appearance of the large subunit at 25 kDa—was stronger and occurred earlier in IL-1β–primed neutrophils (peak at 10 min) compared to unprimed neutrophils (cleavage observed at 60 min) (**new Figure 5F**). The reason for this difference is unclear, and an unspecific band was observed in primary neutrophils exposed to sCD95L (indicated by a star in the immunoblot)

Can the authors comment on the disjointed ROS curves in Figure 6. What causes the large fluctuations in signal across the 1200s timecourse?

ROS and calcium responses were monitored in individual cells in which these responses do not occur with the same kinetics. Therefore, the observed variations are associated with the asynchronous and heterogeneous nature of the cell population, as well as differences in probe loading. Additionally, calcium and ROS signals in neutrophils exposed to sCD95L also depend on inter-patient and donor variability.
In Otulin-knockout HL60 cells, we observed a stronger ROS production than in wild-type cells confirming that the loss of otulin enhances the CD95-induced ROS production by the mitochondria (**Figure 6C**).

Figure 6D requires a positive control – for the ALPS-CASP10 samples, do these cells have more general defects in generating ROS?

These cells were generated from rare ALPS patients. While we cannot completely exclude that other mutations might contribute to the different pattern of ROS production, this is unlikely as the primary germline mutation identified in these patients occurs in CD95 gene. As abovementioned, we did not identify the LUBAC-targeted molecules involved in the activation of the mitochondrial ROS production in AAV neutrophils, rendering difficult to check if this pathway is unaffected in ALPS patients. Nonetheless, these patients have been selected based on the presence of a selective mutation in caspase-10.
Importantly, in the new **figure 6E**, our positive control used cells generated from healthy donors, in which robust ROS production was clearly observed.

For CRISPR ko of HL60 cells, did the three clones from each KO respond in a similar manner? What data supports the knockout of these genes? Did the loss of Casp8 impact the growth or viability of CRISPR ko cells via RIPK1/RIPK3/MLKL?

We generated caspase-10, caspase-8, caspase-3 and otulin ko cell populations with Synthego (Synthego Corporation, CA USA) to avoid clonal effect. **Caspase-8, caspase-10, caspase-3, and Otulin ko have been validated by immunoblotting and these data have been included in the**

revised version of the manuscript (New Figure 6B). Material and methods have been updated accordingly (**page 33 of the revised manuscript**).

We did not observe increased cell death in caspase-8– or caspase-10–knockout cells compared with their parental counterparts (data not shown). Undifferentiated HL-60 cells proliferated more rapidly than DMSO-differentiated cells (**Rebuttal Letter Figure 6**). Loss of caspase-8 or caspase-10 did not significantly affect HL-60 cell growth, although a modest but statistically significant reduction in proliferation was observed in caspase-10–KO cells following DMSO differentiation compared with parental cells. In contrast, deletion of either caspase-3 or OTULIN led to reduced cell growth relative to parental controls (**Rebuttal Letter Figure 6**). Although these findings are intriguing, further studies are required to elucidate the mechanisms by which OTULIN and caspase-3 regulate HL-60 cell proliferation.

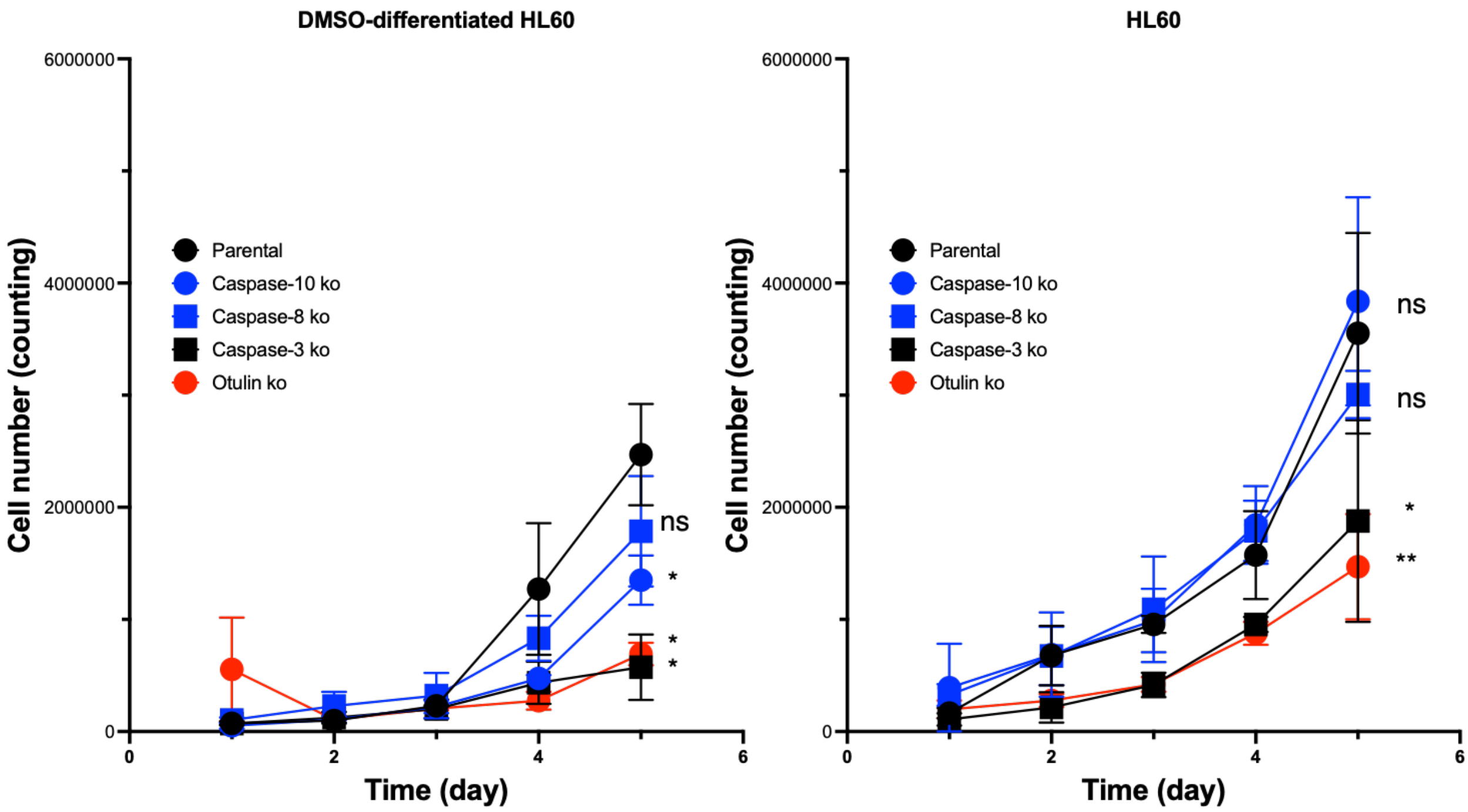

Rebuttal letter figure 6: Cell proliferation of HL60 cells.
Cell growth of the indicated HL60 cell lines was assessed by cell counting. Data represent the mean ± SD of three independent experiments. Proliferation curves were compared using two-way ANOVA.

Does DB550 have impacts on other aspects of the hematological, immune, and inflammatory response in the experiments illustrated in Figure 7?
It seemed unusual for Figure 7 to be split across 3 pages so less clear if these will be an appropriate size if published.

As requested by the reviewer, we reproduced the *in vivo* experiment with the anti-MPO animal model and performed a comprehensive analysis of the immune landscape in the spleen of untreated and DB550-treated animals using multiparameter flow cytometry.
These data clearly indicated that neutrophils are significantly increased in AAV mice compared to control animals (**rebuttal letter table 1 and table 6**) confirming the central role of these cells in ANCA vasculitis[21]. Interestingly, the DB550 treatment reduced the number of neutrophils in the spleen compared to untreated AAV mice (**rebuttal letter table 1 and table 6**). Nonetheless, this effect was not statistically significant. Overall, these findings confirmed that DB550 treatment not only inhibited the CD95-mediated ROS production in neutrophils but also affected their trafficking into inflamed organs (**new figures S6J and S8A**).
On the other hand, DB550 treatment did not affect the proportion of the other immune cells in this AAV animal model, confirming that the neutrophils are the main target of the sCD95L-mediated pro-inflammatory signal in AAV mice.

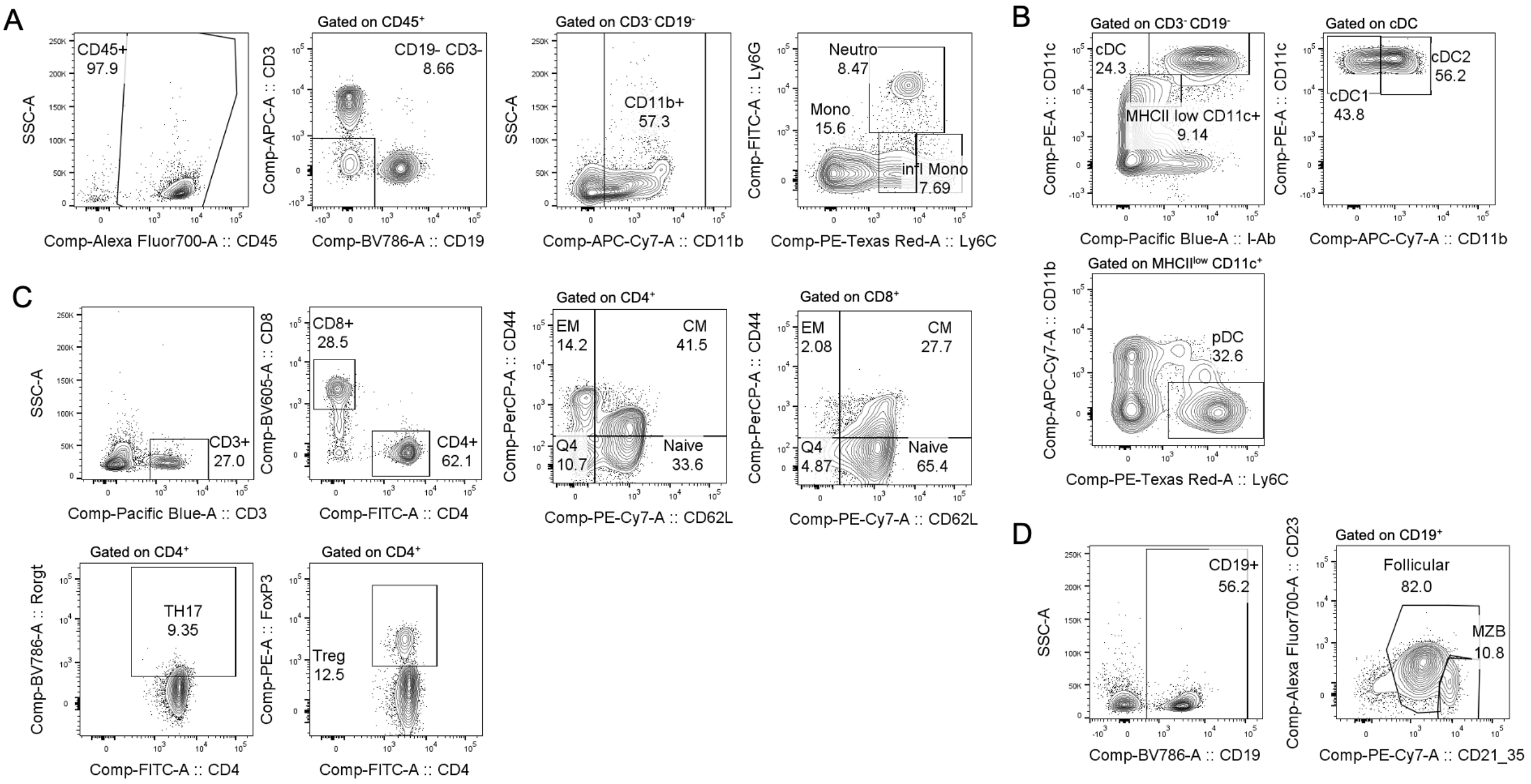

Rebuttal letter Figure 7. Spleen immune cell gating strategy in control mice: Immune cell proportion were assessed by flow cytometry:
A) Gating strategy for monocytes, inflammatory monocytes and neutrophils.
B) Gating strategy for dendritic cell subset.
C) Gating strategy for T cells subset.
D) Gating strategy for B cell subset

		Control n=3		*AAV n=3*			*AAV+ DB550 n=3*		
Population	*Gating strategy*	Mean (%)	SD	Mean (%)	SD	P-Value to control	Mean (%)	SD	P-Value to control
Monocytes	CD19$^-$ CD3$^-$ CD11b$^+$ Ly6G$^-$ Ly6C$^+$ among CD45$^+$	0,7033	0,09866	0,7767	0,1518	>0,9999	0,7833	0,07371	0,6611
Inflammatory Monocytes	CD19$^-$ CD3$^-$ CD11b$^+$ Ly6G$^-$ Ly6C^{hi} among CD45$^+$	0,36	0,1015	1,903	0,4315	0,0738	1,83	0,2982	0,1053
Neutrophils	CD19$^-$ CD3$^-$ CD11b$^+$ Ly6G$^-$ Ly6C$^-$ among CD45$^+$	0,63	0,3637	6,083	1,438	***0,0141***	3,907	0,185	0,3558
Conventional dendritic cells (cDC)	CD19$^-$ CD3$^-$ CD11c^{hi} MHCII^{hi} among CD45$^+$	1,52	0,5129	2,08	0,3132	0,3594	1,77	0,3274	>0,9999
cDC1	CD19$^-$ CD3$^-$ CD11c^{hi} MHCII^{hi} CD11b$^+$ among CD45$^+$	0,64	0,2443	0,8067	0,1422	0,5231	0,7033	0,1484	>0,9999
cDC2	CD19$^-$ CD3$^-$ CD11c^{hi} MHCII^{hi} CD11b$^-$ among CD45$^+$	0,88	0,2689	1,273	0,171	0,1993	1,063	0,1779	0,8206
Plasmacytoid DC	CD19$^-$ CD3$^-$ CD11c$^+$ MHCII^{low} Ly6C$^+$ CD11b$^-$ among CD45$^+$	0,1967	0,05508	0,44	0,1453	0,1053	0,3967	0,1815	0,2021
Follicular B cells	CD23$^+$ CD21/35$^+$ among CD19$^+$	81,03	1,06	81,13	2,635	>0,9999	79,17	1,361	0,3594
Marginal zone B cell	CD23$^-$ CD21/35^{hi} among CD19$^+$	10,54	1,909	1,909	2,248	0,1053	16,5	1,587	0,0738
Central memory CD4 T cell	CD44$^+$ CD62L$^+$ among CD4$^+$	39,57	17,84	21,77	4,823	0,7422	16,67	3,536	0,0507
Effector memory CD4 T cell	CD44$^+$ CD62L$^-$ among CD4$^+$	14,63	2,316	15,63	1,553	>0,9999	14,13	1,002	>0,9999
Naive CD4 T cell	CD44$^-$ CD62L$^+$ among CD4$^+$	33,9	8,302	35,47	1,501	0,7422	29,73	0,9504	0,7422
T_{H17}	RORγt$^+$ among CD4$^+$	9,337	1,975	6,653	1,718	0,5934	4,96	0,03	0,0225
T_{reg}	FoxP3$^+$ among CD4$^+$	12,83	1,724	18,23	3,323	0,1053	16,87	1,888	0,2021
Central memory CD8 T cell	CD44$^+$ CD62L$^+$ among CD8$^+$	26,8	5,209	21,27	3,182	0,4661	22,93	3,623	0,5934
Effector memory CD8 T cell	CD44$^+$ CD62L$^-$ among CD8$^+$	1,85	0,4694	3,857	2,225	0,2021	3,817	0,1779	0,2721
Naive CD8 T cell	CD44$^-$ CD62L$^+$ among CD8$^+$	67,57	3,329	64,37	8,043	0,7422	59,53	5,229	0,1473

Table 1 (Manuscript new Table 6). Immune cell proportions in control, AAV, and AAV + DB550-treated mice. The relative proportions of splenic immune cell subsets were determined by multiparameter flow cytometry. Immune populations were defined according to the gating strategy described in the Methods. Statistical analysis was performed using a non-parametric one-way ANOVA followed by Dunn's multiple-comparisons test. Indicated *P* values correspond to comparisons *versus* control mice. Data are presented as mean ± s.d. Bold italic values denote statistically significant differences. Experiments were performed independently three times.

Confirm if mouse models used 0.5ng/g or 0.5ug/g LPS.

This has been corrected in the manuscript, we injected 10 μg per mouse so 0.5 μg/g or 0.5 mg/kg.

Reviewer #3 (Remarks to the Author):

The manuscript presents an intriguing and potentially impactful study on the role of CD95/CD95L signaling in ANCA-associated vasculitis (AAV), with a focus on the contribution of soluble CD95L (sCD95L) to neutrophil activation and ROS production via caspase-dependent mechanisms. The identification of **OTULIN as a novel caspase substrate and the involvement of LUBAC in mitochondrial ROS generation are particularly interesting observations that could have therapeutic implications.**

However, I have major concerns regarding the mechanistic conclusions, particularly those involving caspase-10, which currently lack sufficient experimental support. In addition, the manuscript is missing methodological details, which must be addressed to support the manuscript's conclusions.
These issues need to be thoroughly addressed before the manuscript can be considered for publication.

Major Concerns:

1. Caspase-10 involvement not clearly demonstrated

The manuscript claims that caspase-10 cleaves OTULIN, leading to LUBAC activation and mitochondrial ROS production in neutrophiles.

o Is there direct evidence of caspase-10 activation (e.g., cleavage, activity assays) in neutrophils treated with sCD95L?

As requested by the referee, we added data showing that caspase-10 is activated in HL-60 cells exposed to sCD95L (**new manuscript Figure 5F**). Conversely, deletion of caspase-10 in HL-60 cells inhibited CD95-mediated ROS production (**new manuscript Figure 6C**). These data indicate that caspase-10 is functionally activated in DMSO-differentiated HL-60 cells.

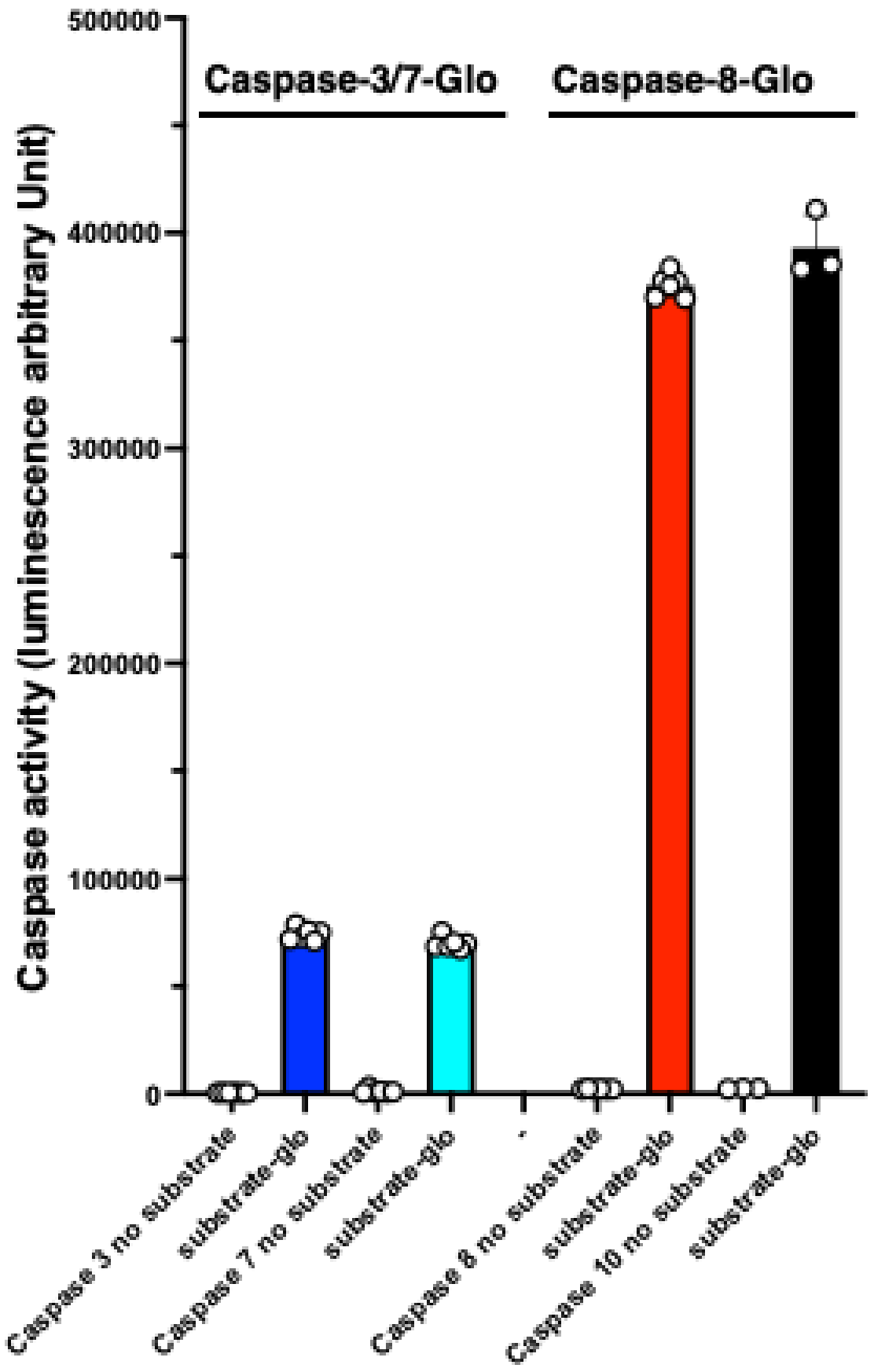

Rebuttal letter figure 8: Validation of the protease activity of caspases 3/7 and caspases 8/10 using caspase-3/7-glo (#G8090, Promega) or caspase-8-glo (#G8200, Promega) assays, respectively.

Establishing a direct role for caspase-10 in OTULIN cleavage is challenging because, as noted by the referee, caspase-8 and caspase-10 act together *in cellulo* and activate downstream effector caspases such as caspase-3 and -7, making it difficult to specifically identify the protease responsible for a given protein cleavage.

To address this issue, we developed a cell free assay to identify the proteases cleaving Otulin at D54 (**Figures 5E-F**). For this assay, HEK/293T cells were transfected with FLAG-tagged wild type or D54E OTULIN construct. These constructs were then immunoprecipitated, beads were extensively washed and incubated with pure and activated initiator caspases 8 and 10 or effector caspases 3 and 7 (**Figures 5D and 5E**). **These experiments established that caspase-10 was the only caspase able to cleave**

otulin at D54. On the other hand, caspases 3, 7 and 10 cleave OTULIN at D31. Caspase-8 did not cleave OTULIN.
In these experiments, we validated that each caspase was functional using the Caspase-3/7 and Caspase-8 Glo assays (**rebuttal letter Figure 7**).

2. Alternative caspases, particularly caspase-8, are not systematically ruled out. This is especially important given that:

• Both caspase-8 and caspase-10 are expressed in neutrophils.

• Caspase-10 is often present at lower concentrations in these cells compared to caspase-8.

• The manuscript does not clearly explain how the decision between caspase-8 and caspase-10 is regulated in this context.

• The use of specific inhibitors, knockdowns, or rescue approaches would be critical to establish caspase-10-caspase-8 crosstalk in these cells and to more convincingly show caspase-10 specificity in neutrophiles.

Although **Figure 6C** (genetic) and **6D** (pharmacologic) demonstrate that both caspase-8 and caspase-10 are required to activate ROS production in HL60 cells and IL-1β/TNFα-primed neutrophils, our **cell free assay** using commercial caspases definitively established that only caspase-10 cleaves OTULIN at D54 (**Figure 5E and 5F**). Of note, the cell free assay revealed that caspase-8 cannot cleave Otulin, whereas caspases 3 and 7 cleave otulin at D31 as previously reported[22].
Although the caspase-8 aggregation is pivotal role for the activation of caspase-10[23], the decision between the caspase-8-mediated apoptotic signal and the caspase-10-driven non-apoptotic responses remains to be elucidated.

We observed that caspase-10 and caspase-8 knockout HL60 cells exposed to sCD95L do not produce ROS whereas caspase-3 knockout cells did it (**Figure 6C**), indicating that the executioner caspase is not involved in the CD95-mediated ROS production in neutrophils. Similarly, both caspase-8 and -10 activities are required to produce ROS, since the pharmacologic inhibitors of caspase-8 and -10 prevented the ROS production in IL1β/TNFα-treated neutrophils isolated from healthy donors and exposed to sCD95L **(Figure 6D**).

Data from ALPS patients with non-functional caspase-10 further confirmed the key role played by caspase-10 in the sCD95L-mediated ROS production.

3. Specificity of Caspase-Mediated OTULIN Cleavage

The cleavage of OTULIN is attributed to caspase-10, but the possibility that other caspases (e.g., caspase-8 or -3) are involved is not adequately ruled out.

As above mentioned, to unambiguously determine which caspase was responsible for otulin cleavage, we developed a cell free assay (**Figures 5D-E**). This assay in which we only mixed activated caspases with purified wild type or mutated OTULINs, revealed that only caspase-10 was able to cleave otulin and to do it at D54 **(Figure 5D-E**). Despite testing purified and active recombinant caspase-8 (**rebuttal letter Figure 8**) from multiple manufacturers, none of them were able to cleave Otulin, ruling out the implication of caspase-8 in the Otulin processing.

o Please clarify whether the cleavage sites identified are unique to caspase-10

As mentioned above, D54 cleavage was only observed with the purified and active caspase-10 (see **Figures 5D and 5E**). Like caspase-3/7, caspase-10 can also cleave at D31 as shown in **manuscript**

figure 5E. Indeed, two bands below the full length OTULIN appeared when wild type OTULIN was mixed with activated caspase-10 (**Figures 5D and 5E**). In addition, while the D31E mutant impaired the appearance of the upper band in the presence of caspase-10 (**Figure 5E**), the D54E mutant abrogated the generation of the lower band in an anti-OTULIN immunoblot (**Figure 5D-E**), The biological role of this double cleavage in neutrophils remain to be elucidated.

4. Causal Link Between CD95/CD95L Signaling and ROS Remains Correlative

While the link between sCD95L and increased ROS in AAV neutrophils is well described, the pathway leading from CD95 engagement to mitochondrial ROS production via OTULIN cleavage is not convincingly demonstrated as a causative chain.

o Additional data are needed to establish this as a functional pathway rather than an association.

We showed that inhibition of LUBAC activity using the HOIP inhibitor HOIPIN-1[24] markedly reduced sCD95L-mediated ROS production in IL1β/TNFα pre-stimulated neutrophils (**manuscript Fig.6A**). Conversely, Otulin knockout increased the sCD95L-mediated ROS production in HL60 cells (**manuscript Fig.6C**), indicating that the removal of OTULIN, a LUBAC inhibitor, unleashes a robust linear ubiquitination (**see manuscript new figure 5I**) and the CD95-mediated ROS production in neutrophils.
We plan to identify the LUBAC-driven ubiquitinated protein(s) responsible for ROS production however this project is clearly beyond the scope of the present study.

5. Recombinant Caspases: Missing Methodological Details

A major concern is the lack of a clear description of recombinant caspase preparation in the Materials and Methods section:

o The manuscript does not specify how recombinant caspases were purified, nor how their activity was verified or normalized across experiments. Specifically, this information is essential for the interpretation of the data in Figure 5

o Since conclusions regarding OTULIN cleavage rely heavily on these enzymes, this missing information is critical and must be included in the revised manuscript.

We apologize for the incomplete description of the caspase assay. We used purified caspases from R&D which were incubated with immunoprecipitated OTULIN constructs (wild type, D54E and D31E constructs, **manuscript Figures 5D and 5E**). The activity of each caspase was verified prior to the assay using the generic caspase substrates of caspase-3/7 and caspase-8 (caspase-glo assays) (**rebuttal letter Figure 8**). These details have been added in the Method part of the revised manuscript (**page 33 of the revised manuscript**)

6. Figure 5G: Ubiquitination Data Not Quantitatively Supported

In Figure 5G, M1-linked ubiquitination is assessed using recombinant proteins, and the authors conclude that the Δ54 OTULIN mutant increases M1-ubiquitin levels by approximately 50%. However, this conclusion is not supported by the data, as:

o The Δ54 construct appears to be expressed at much higher levels than both WT and Δ31.
o No normalization is shown to account for differences in protein expression, making it unclear whether the observed increase is due to enhanced activity or simply overexpression.

o As such, the conclusion regarding the 50% increase in M1-ubiquitination is not experimentally justified and should be re-evaluated or removed unless properly controlled.

To address this concern, we performed a dose effect of the different OTULIN constructs in HEK/293T cells. Briefly, we analyzed the dose effect of wild-type and truncated Δ54 and Δ31 OTULIN constructs in HEK293T cells co-transfected with the LUBAC complex, including HOIP, HOIL-1, and SHARPIN (**new manuscript Figure 5I**). While the wild-type and Δ31 OTULIN constructs efficiently inhibited LUBAC-mediated linear ubiquitination in these cells even at low doses, the Δ54 construct was less efficient, confirming that cleavage of OTULIN at D54 impairs its deubiquitinase activity. This has now been added to the Results section (**pages 16 and 17 in the revised manuscript**).

6. Limited OTULIN Cleavage in Patient Samples Challenges the Proposed Mechanism The data presented in Figure 6 showing OTULIN cleavage in patient samples is valuable and adds clinical relevance to the study.
However, a notable contradiction arises:

Only approximately 10% of OTULIN is cleaved, while 90% remains uncleaved in these samples. This raises an important question about the threshold of OTULIN cleavage required to meaningfully impair its function and to trigger the downstream effects on LUBAC activity and ROS production. It also suggests that OTULIN cleavage may not be the sole mechanism controlling ROS generation upon sCD95L treatment. Additional or complementary pathways may contribute, which should be acknowledged and explored.

We agree that the cleavage of OTULIN is not a black-and-white phenomenon. However, we can envision that distinct pools of OTULIN exist within the cell, and that CD95 gains access to a limited fraction of OTULIN, which is necessary and sufficient to activate LUBAC activity responsible for mitochondrial ROS production. This hypothesis is discussed in the manuscript (**page 25 of the revised version**).

7. Typographical and grammatical errors:

The manuscript contains language issues that should be addressed through proofreading.

** See Nature Portfolio’s author and referees' website at www.nature.com/authors for information about policies, services and author benefits.

This email has been sent through the Springer Nature Tracking System NY-610A-NPG&MTS

Confidentiality Statement:

This e-mail is confidential and subject to copyright. Any unauthorised use or disclosure of its contents is prohibited. If you have received this email in error please notify our Manuscript Tracking System Helpdesk team at http://platformsupport.nature.com .

Details of the confidentiality and pre-publicity policy may be found here http://www.nature.com/authors/policies/confidentiality.html

Privacy Policy | Update Profile

References

1. Koenderman L, Tesselaar K, Vrisekoop N. Human neutrophil kinetics: a call to revisit old evidence. *Trends Immunol* **43**, 868–876 (2022).

2. Schreiber A, Luft FC, Kettritz R. Phagocyte NADPH oxidase restrains the inflammasome in ANCA-induced GN. *J Am Soc Nephrol* **26**, 411–424 (2015).

3. Nauseef WM. The phagocyte NOX2 NADPH oxidase in microbial killing and cell signaling. *Curr Opin Immunol* **60**, 130–140 (2019).

4. Cipriano A*, et al.* NADPH Oxidases: From Molecular Mechanisms to Current Inhibitors. *J Med Chem* **66**, 11632–11655 (2023).

5. Dupont PJ, Warrens AN. Fas ligand exerts its pro-inflammatory effects via neutrophil recruitment but not activation. *Immunology* **120**, 133–139 (2007).

6. Croker BA*, et al.* Fas-mediated neutrophil apoptosis is accelerated by Bid, Bak, and Bax and inhibited by Bcl-2 and Mcl-1. *Proc Natl Acad Sci U S A* **108**, 13135–13140 (2011).

7. Falk RJ, Terrell RS, Charles LA, Jennette JC. Anti-neutrophil cytoplasmic autoantibodies induce neutrophils to degranulate and produce oxygen radicals in vitro. *Proc Natl Acad Sci U S A* **87**, 4115–4119 (1990).

8. Tauzin S*, et al.* The naturally processed CD95L elicits a c-yes/calcium/PI3K-driven cell migration pathway. *PLoS Biol* **9**, e1001090 (2011).

9. Poissonnier A*, et al.* CD95-Mediated Calcium Signaling Promotes T Helper 17 Trafficking to Inflamed Organs in Lupus-Prone Mice. *Immunity* **45**, 209–223 (2016).

10. Poissonnier A*, et al.* Disrupting the CD95-PLCgamma1 interaction prevents Th17-driven inflammation. *Nat Chem Biol* **14**, 1079–1089 (2018).

11. Malleter M*, et al.* CD95L cell surface cleavage triggers a prometastatic signaling pathway in triple-negative breast cancer. *Cancer Res* **73**, 6711–6721 (2013).

12. Drachsler M*, et al.* CD95 maintains stem cell-like and non-classical EMT programs in primary human glioblastoma cells. *Cell Death Dis* **7**, e2209 (2016).

13. Teodorczyk M*, et al.* CD95 promotes metastatic spread via Sck in pancreatic ductal adenocarcinoma. *Cell Death Differ* **22**, 1192–1202 (2015).

14. Letellier E*, et al.* CD95-ligand on peripheral myeloid cells activates Syk kinase to trigger their recruitment to the inflammatory site. *Immunity* **32**, 240–252 (2010).

15. Corsini NS, *et al.* The death receptor CD95 activates adult neural stem cells for working memory formation and brain repair. *Cell stem cell* **5**, 178–190 (2009).

16. Kleber S, *et al.* Yes and PI3K bind CD95 to signal invasion of glioblastoma. *Cancer Cell* **13**, 235–248 (2008).

17. Mol S, *et al.* Efficient Neutrophil Activation Requires Two Simultaneous Activating Stimuli. In: *Int J Mol Sci*) (2021).

18. Fortunati E, Kazemier KM, Grutters JC, Koenderman L, Van den Bosch v J. Human neutrophils switch to an activated phenotype after homing to the lung irrespective of inflammatory disease. In: *Clin Exp Immunol*) (2009).

19. Daburon S, *et al.* Functional characterization of a chimeric soluble Fas ligand polymer with in vivo anti-tumor activity. *PLoS One* **8**, e54000 (2013).

20. Rasola A, Geuna M. A flow cytometry assay simultaneously detects independent apoptotic parameters. *Cytometry* **45**, 151–157 (2001).

21. Huang F, Lv Y, Liu S, Wu H, Liu Q. Animal models for anti-neutrophil cytoplasmic antibody-associated vasculitis: Are current models good enough? *Animal Model Exp Med* **6**, 452–463 (2023).

22. Douglas T, Saleh M. Post-translational Modification of OTULIN Regulates Ubiquitin Dynamics and Cell Death. *Cell reports* **29**, 3652–3663 e3655 (2019).

23. Horn S, *et al.* Caspase-10 Negatively Regulates Caspase-8-Mediated Cell Death, Switching the Response to CD95L in Favor of NF-kappaB Activation and Cell Survival. *Cell reports* **19**, 785–797 (2017).

24. Oikawa D, *et al.* Molecular bases for HOIPINs-mediated inhibition of LUBAC and innate immune responses. *Commun Biol* **3**, 163 (2020).

Before we start answering the questions raised by the reviewers, we would like to thank reviewers for the work they accomplished on this study and the constructive remarks that will improve this manuscript.

RESPONSE TO REVIEWERS' COMMENTS

Reviewer #1 (Remarks to the Author):

The authors have provided a clear and thoughtful response to the reviewers' comments, and the revisions have strengthened the manuscript, particularly the inclusion of additional experiments in primary neutrophils. Overall, the authors have addressed my comments satisfactorily and I have only a few minor points:

1. The authors included a detailed analysis of patient characteristics and the association with sCD95L in the response to reviewers. While I appreciate this is not the main focus of the study it would be helpful to add a brief statement to the manuscript to highlight that within the limitations of the sample size no obvious clinical/confounding factors were identified to explain differences in sCD95L.

A statement addressing this point had been incorporated into the revised manuscript (lines 146–149). In addition, we included Supplementary Figure 2, which presents analyses of sCD95L concentrations according to multiple clinical parameters and shows that, within the limitations of the cohort size, no obvious clinical or confounding factors were identified that could explain differences in sCD95L levels.

2. The mouse accelerated NTN study has been moved to the supplementary figures but the main text still contains a relatively detailed description of these data. As this experiment provides some additional information not assessed in the AAV models (e.g., neutrophil Ca^{2+} flux and ROS), it would be helpful to revise the text to more specifically highlight these unique aspects and reduce overlap with the AAV model description. As noted previously, I would also avoid referring to neutrophils from NTN mice as "AAV-like."

As requested, we have revised the main text and reorganized the figures to position this model at the end of the study. We now more clearly explain the rationale for using this model, namely to evaluate the therapeutic effect of DB550 on neutrophil trafficking in a kidney pathology characterized by glomerular crescent formation.

In addition, we have rationalized the description of these data to emphasize the specific insights provided by this model (e.g., neutrophil Ca^{2+} flux and ROS production), while reducing redundancy with the two AAV models.

Finally, the terminology "AAV-like" has been replaced with "anti-GBM mouse model" throughout, and its use has been clarified.

3. In Fig S6F please could you clarify if healthy neutrophils or NTN neutrophils are used?

This panel is now presented as Fig. S8F. Neutrophils were isolated from untreated mice and subsequently primed with TNFα/IL-1β for 15 minutes prior to stimulation with sCD95L (100 ng/mL).

The figure legend has been revised accordingly to clarify this point.

Reviewer #2 (Remarks to the Author):

Concerns have been adequately addressed.

Reviewer #4 (Remarks to the Author):

The authors have responded to all of the three reviewers comments and improved the manuscript.

I was asked to assess the authors responses to the critiques by reviewer #3. These focus in great part on the role of caspase-10 and the level of OTULIN cleavage in patients.

I share this reviewers concerns and would like to specify them a bit more. My understanding is the author believe that the function of caspase-10 is replaced by caspase-8 in mice. That is also consistent with the dependency of ROS production in both human and mice on caspase-8, further supported by the fact that the pan-caspase inhibitor zVAD-fmk does not inhibit caspase-10. So, it is caspase-8 that is critical in both species. What is hard to understand is why caspase-8 would not cleave OTULIN D54 in humans if it is essential for the cleavage of mouse Otulin. Especially because the 14 amino acids around the D54 cleavage site are identical in human and mouse Otulin.

We thank the reviewer for this insightful comment.

We indeed hypothesize that caspase-8 may functionally compensate for the absence of caspase-10 in mice with respect to OTULIN cleavage at D54. This hypothesis is supported by several observations. First, mice lack a caspase-10 ortholog, and murine caspase-8 is the closest functional homolog (mouse caspase-8 shows a sequence identity with human caspase-10 of 35.68%). Second, inhibition of caspase-8 using zIETD-fmk significantly reduces CD95-induced ROS production in mouse neutrophils (Supplementary Fig. 8E), consistent with a central role for this protease in this pathway.
It is noteworthy that, the small three peptide, zVAD-fmk is a pan-caspase inhibitor, which also inhibits caspase-10 activity[1].

However, we would like to emphasize that our data do not directly demonstrate that murine caspase-8 cleaves OTULIN at D54. As the reviewer correctly points out, the conservation of the cleavage site between human and mouse OTULIN (see alignment figure) would suggest that if mouse caspase-8 is involved in OTULIN cleavage, it could perform this function in human cells as well. Nonetheless, our cell-free assays established that human caspase-8 is unable to cleave OTULIN.
One possible explanation is that, activated caspase-8 mainly acts upstream by enabling caspase-10 activation (or other caspases in mice) rather than directly mediating substrate cleavage. Indeed, caspase-8 is known to form activation platforms involving c-FLIP[2, 3] and activating caspase-10[4]. In this context, inhibition of caspase-8 activity, using zIETD-fmk, or elimination of its expression by CRISPR/Cas9, would indirectly prevent OTULIN cleavage by blocking the downstream activation of caspase-10 in human neutrophils or other caspases in mice.

I agree the human and mouse OTULIN sequences are well conserved (90.06% identity) but some differences exist in their N-terminal regions (see alignment figure below). In addition, human and mouse caspase-8 only share 66.74% of identity, and this sequence divergence may contribute to species-specific substrate preferences and functional compensation mechanisms in mice.

Altogether, while our data support a role for caspase-8 in the murine system, the precise identity of the protease responsible for OTULIN cleavage in mice remains to be formally established. Addressing this question would require substantial additional investigation and is beyond the scope of the recent study.

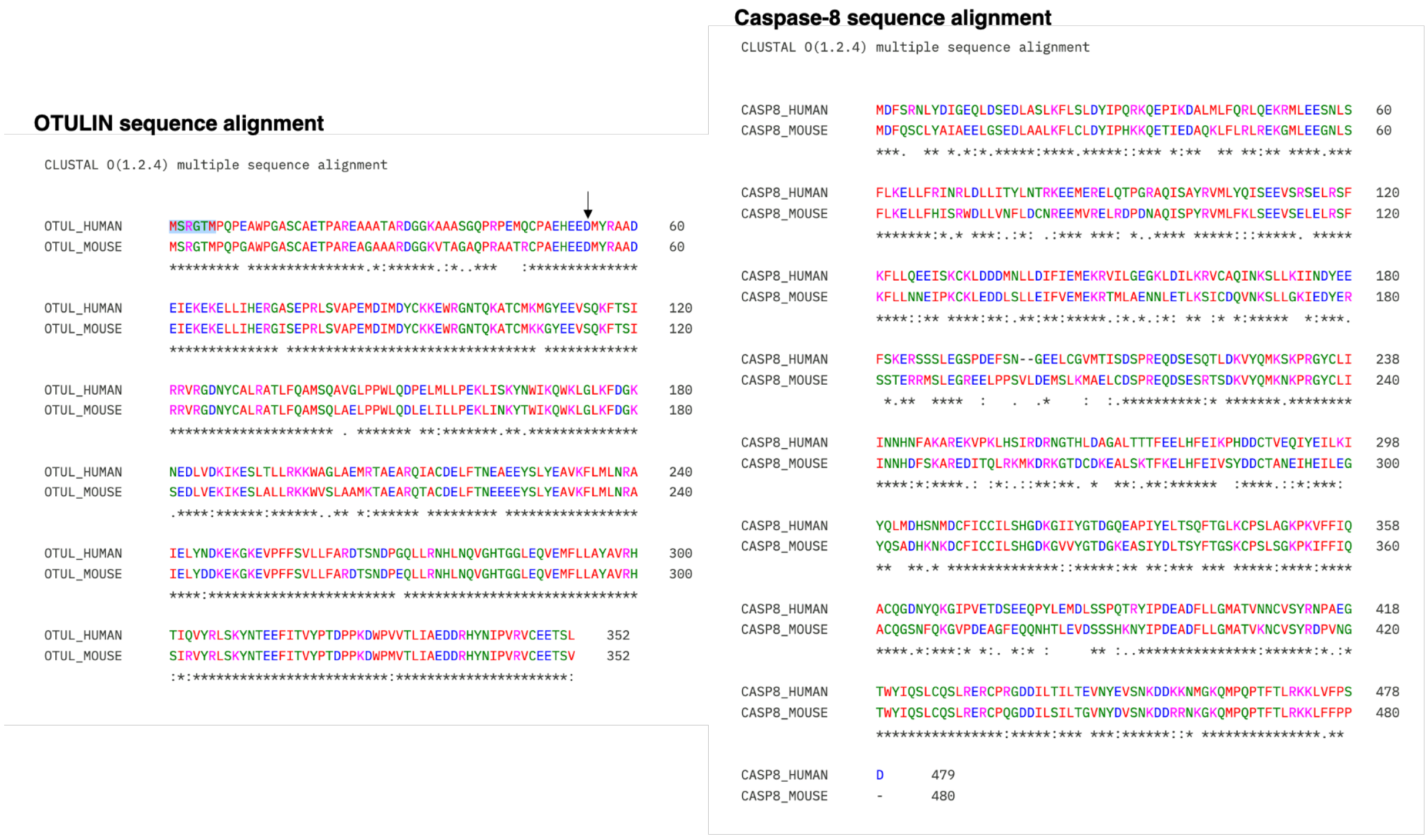

Figure. Alignment of mouse and human OTULIN and caspase-8 proteins.
The arrow indicates the newly identified caspase-driven D54 position.

I appreciate the addition of the data on the cell-free system in Figure 5. The cell-free system provides an opportunity to figure out whether murine caspase-8 cleaves murine OTULIN which has to be predicted. That could be added and would address most of reviewer #3's concerns.

As abovementioned, although our data suggest that caspase-8 may play this role, since zIETD-fmk, a selective inhibitor of caspase-8 activity (Supplementary Figure 8E), inhibits the CD95-mediated ROS production in mouse neutrophils, we cannot rule out the involvement of another caspase to fulfill the role of human caspase-10.

We agree that our cell-free system represents a clean and interesting approach to directly assess whether murine caspase-8 can cleave murine OTULIN. However, we will have also to evaluate the effect of the other mouse caspases (i.e., caspases -3, and -7) and performing and validating such biochemical assays would require substantial additional times and work, including protein purification and optimization of cleavage conditions.

While addressing the caspase involved in OTULIN cleavage in mice is an interesting question, we believe it extends beyond the scope of the current study, which focuses on the functional consequences of CD95 signaling in neutrophils from AAV patients. We therefore consider this an important direction for future investigation.

A few general remarks:

The abstract is not accessible to a nonspecialized reader. ANCA and LUBAC are not explained.

This has been modified.

I do not understand why none of the figures presented to the reviewers in the rebuttal made it into the supplement? Some of these data need to be shown there.

We appreciate this point. While we aimed to keep the number of supplementary figures within the recommendations of the journal, we agree that some of the data shown during the review process are valuable.

Accordingly, we have incorporated key additional datasets into the revised manuscript and supplementary materials (including the immune landscape analysis in AAV mice treated or not with DB550, now presented as new Table 1). We have prioritized inclusion of the most informative data while maintaining clarity and readability.

There is a bit of a disconnect between the in vitro and the in vivo parts of the manuscript. The effects of the CD95 interacting peptide on the disease outcome in the different mouse models are not directly connected to the cleavage of Otulin by caspase-10 or in this case caspase-8.

DB550 selectively inhibits CD95-mediated non-apoptotic Ca^{2+} signaling, which in turn reduces ROS production in neutrophils. While we have identified caspase-dependent mechanisms contributing to ROS generation, the precise molecular interplay between caspase-10-driven LUBAC activation and Ca^{2+} signaling remains to be fully elucidated.
We now explicitly acknowledge this limitation in the revised manuscript and highlight it as an important direction for future research.

** See Nature Portfolio's author and referees' website at www.nature.com/authors for information about policies, services and author benefits

This email has been sent through the Springer Nature Tracking System NY-610A-NPG&MTS

Confidentiality Statement:

This e-mail is confidential and subject to copyright. Any unauthorised use or disclosure of its contents is prohibited. If you have received this email in error please notify our Manuscript Tracking System Helpdesk team at http://platformsupport.nature.com .

Details of the confidentiality and pre-publicity policy may be found here http://www.nature.com/authors/policies/confidentiality.html

Privacy Policy | Update Profile

References

1. Chauvier D, Ankri S, Charriaut-Marlangue C, Casimir R, Jacotot E. Broad-spectrum caspase inhibitors: from myth to reality? In: *Cell Death Differ*) (2007).

2. Fu TM*, et al.* Cryo-EM Structure of Caspase-8 Tandem DED Filament Reveals Assembly and Regulation Mechanisms of the Death-Inducing Signaling Complex. *Mol Cell* **64**, 236–250 (2016).

3. Fox JL*, et al.* Cryo-EM structural analysis of FADD:Caspase-8 complexes defines the catalytic dimer architecture for co-ordinated control of cell fate. *Nat Commun* **12**, 819 (2021).

4. Horn S*, et al.* Caspase-10 Negatively Regulates Caspase-8-Mediated Cell Death, Switching the Response to CD95L in Favor of NF-kappaB Activation and Cell Survival. *Cell reports* **19**, 785–797 (2017).
