# Supplementary material for: Soluble CD95L triggers Caspase-10-driven reactive oxygen species production in neutrophils and aggravates anti-neutrophil cytoplasmic antibody-vasculitis
Source: Nat Commun. 2026 Jun 20;17:7759. doi: 10.1038/s41467-026-74452-8 (PMC13434393; doi:10.1038/s41467-026-74452-8)
Supplement: Supplementary file 3 [file 41467_2026_74452_MOESM3_ESM.pdf]

nature portfolio

Corresponding author(s): Legembre patrick

Last updated by author(s): Jul 10, 2025

Reporting Summary

Nature Portfolio wishes to improve the reproducibility of the work that we publish. This form provides structure for consistency and transparency in reporting. For further information on Nature Portfolio policies, see our Editorial Policies and the Editorial Policy Checklist.

Statistics

For all statistical analyses, confirm that the following items are present in the figure legend, table legend, main text, or Methods section.

n/a	Confirmed	
☐	☒	The exact sample size (*n*) for each experimental group/condition, given as a discrete number and unit of measurement
☐	☒	A statement on whether measurements were taken from distinct samples or whether the same sample was measured repeatedly
☐	☒	The statistical test(s) used AND whether they are one- or two-sided<br>*Only common tests should be described solely by name; describe more complex techniques in the Methods section.*
☒	☐	A description of all covariates tested
☒	☐	A description of any assumptions or corrections, such as tests of normality and adjustment for multiple comparisons
☐	☒	A full description of the statistical parameters including central tendency (e.g. means) or other basic estimates (e.g. regression coefficient) AND variation (e.g. standard deviation) or associated estimates of uncertainty (e.g. confidence intervals)
☐	☒	For null hypothesis testing, the test statistic (e.g. *F*, *t*, *r*) with confidence intervals, effect sizes, degrees of freedom and *P* value noted<br>*Give P values as exact values whenever suitable.*
☒	☐	For Bayesian analysis, information on the choice of priors and Markov chain Monte Carlo settings
☒	☐	For hierarchical and complex designs, identification of the appropriate level for tests and full reporting of outcomes
☐	☒	Estimates of effect sizes (e.g. Cohen's *d*, Pearson's *r*), indicating how they were calculated

Our web collection on statistics for biologists contains articles on many of the points above.

Software and code

Policy information about availability of computer code

Data collection	Not relevant
Data analysis	Not relevant

For manuscripts utilizing custom algorithms or software that are central to the research but not yet described in published literature, software must be made available to editors and reviewers. We strongly encourage code deposition in a community repository (e.g. GitHub). See the Nature Portfolio guidelines for submitting code & software for further information.

Data

Policy information about availability of data

All manuscripts must include a data availability statement. This statement should provide the following information, where applicable:

- Accession codes, unique identifiers, or web links for publicly available datasets
- A description of any restrictions on data availability
- For clinical datasets or third party data, please ensure that the statement adheres to our policy

no restriction on data availability

Research involving human participants, their data, or biological material

Policy information about studies with human participants or human data. See also policy information about sex, gender (identity/presentation), and sexual orientation and race, ethnicity and racism.

Reporting on sex and gender	yes
Reporting on race, ethnicity, or other socially relevant groupings	no (impossible to do that in France)
Population characteristics	no
Recruitment	ANCA vasculitis patients were recruited at Bordeaux and Angers Hospitals. Fresh healthy adult donor blood samples were obtained from a blood bank ("Etablissement Français du Sang Nouvelle-Aquitaine"; EFS-NAQ, Bordeaux, France).
Ethics oversight	no

Note that full information on the approval of the study protocol must also be provided in the manuscript.

Field-specific reporting

Please select the one below that is the best fit for your research. If you are not sure, read the appropriate sections before making your selection.

[x] Life sciences [ ] Behavioural & social sciences [ ] Ecological, evolutionary & environmental sciences

For a reference copy of the document with all sections, see nature.com/documents/nr-reporting-summary-flat.pdf

Life sciences study design

All studies must disclose on these points even when the disclosure is negative.

Sample size	disclosed
Data exclusions	no data exclusion
Replication	For each figure, experiments have been performed independently at least three times.
Randomization	The mice were randomized into different groups before treatment.
Blinding	Blinding has been performed for the immunohistological analyses of mouse experiments.

Behavioural & social sciences study design

All studies must disclose on these points even when the disclosure is negative.

Study description	n/a
Research sample	n/a
Sampling strategy	n/a
Data collection	n/a
Timing	n/a
Data exclusions	n/a
Non-participation	n/a
Randomization	n/a

Ecological, evolutionary & environmental sciences study design

All studies must disclose on these points even when the disclosure is negative.

Study description	n/a

Research sample	n/a
Sampling strategy	n/a
Data collection	n/a
Timing and spatial scale	n/a
Data exclusions	n/a
Reproducibility	n/a
Randomization	n/a
Blinding	n/a

Did the study involve field work? ☐ Yes ☒ No

Reporting for specific materials, systems and methods

We require information from authors about some types of materials, experimental systems and methods used in many studies. Here, indicate whether each material, system or method listed is relevant to your study. If you are not sure if a list item applies to your research, read the appropriate section before selecting a response.

Materials & experimental systems

n/a	Involved in the study	
☐	☒	Antibodies
☐	☒	Eukaryotic cell lines
☒	☐	Palaeontology and archaeology
☐	☒	Animals and other organisms
☐	☒	Clinical data
☒	☐	Dual use research of concern
☒	☐	Plants

Methods

n/a	Involved in the study	
☒	☐	ChIP-seq
☐	☒	Flow cytometry
☒	☐	MRI-based neuroimaging

Antibodies

Antibodies used	All commercial antibodies have been used according to the manufacturer's recommendations
Validation	We bought well known and already used antibodies (e.g., caspase-8, anti-CD95L, anti-V5, anti-FLAG...). All commercial antibodies have been used according to the manufacturer's recommendations

Eukaryotic cell lines

Policy information about cell lines and Sex and Gender in Research

Cell line source(s)	ATCC
Authentication	Cell lines were provided by ATCC and no authentication has been done
Mycoplasma contamination	Cell lines were tested each month using PCR tests and contamination detection has been associated with treatment (Plasmocin and Plasmocure from InvivoGen).
Commonly misidentified lines (See ICLAC register)	no use of commonly misidentified lines.

Palaeontology and Archaeology

Specimen provenance	n/a
Specimen deposition	n/a
Dating methods	n/a

☐ Tick this box to confirm that the raw and calibrated dates are available in the paper or in Supplementary Information.

Ethics oversight	n/a

Note that full information on the approval of the study protocol must also be provided in the manuscript.

Animals and other research organisms

Policy information about studies involving animals; ARRIVE guidelines recommended for reporting animal research, and Sex and Gender in Research

Laboratory animals	C57BL/6J mice (6–8 weeks were purchased from Charles River Laboratories (Les Oncins, France).
Wild animals	no wild animals
Reporting on sex	Female
Field-collected samples	No field-collected samples
Ethics oversight	All treatment protocols were reviewed and approved by the Institutional Animal Ethics Committee (authorization numbers: APAFIS #19506 and #48633).

Note that full information on the approval of the study protocol must also be provided in the manuscript.

Clinical data

Policy information about clinical studies

All manuscripts should comply with the ICMJE guidelines for publication of clinical research and a completed CONSORT checklist must be included with all submissions.

Clinical trial registration	The retrospective serum collection came from the Bordeaux University Hospital biobank. To enroll additional patients, a research protocol named VASC-FAS conforming to French and European Ethics standards was reviewed and approved by an independent ethics committee (Bordeaux cohort, ethical approval authorization number: 18/07/23/39007). The VASC-FAS research protocol was registered at ClinicalTrials. gov (number: NCT03698071).<br>To confirm the results obtained in the Bordeaux cohort, sCD95L was assessed in a longitudinal cohort of patients with AAV before and after immunosuppressive treatment (at 0 and 6 months after treatment) from the Angers University Hospital (Cohort Maine-Anjou, Ethical authorization number 2018-MR03-02, Personal Protection Committee CB 2015/09).
Study protocol	The VASC-FAS research protocol was registered at ClinicalTrials. gov (number: NCT03698071).<br>Cohort Maine-Anjou, Ethical authorization number 2018-MR03-02, Personal Protection Committee CB 2015/09
Data collection	For healthy donors: Fresh healthy adult donor blood samples were obtained from a blood bank (“Etablissement Français du Sang Nouvelle-Aquitaine”; EFS-NAQ, Bordeaux, France). The EFS-NAQ displays an agreement with the French Ministry of Health and Research (agreement number AC 2018-3149) to collect additional blood volume from healthy donors who do not oppose the use of these samples for research purposes.<br>Patients with ANCA-associated vasculitis were recruited from the rheumatology, nephrology, and internal medicine departments of Bordeaux University Hospital. The patients were diagnosed with vasculitis using the 2012 Revised International Chapel Hill Consensus Conference Nomenclature criteria.
Outcomes	In the two patient cohorts, AAV activity was assessed using the 3.0 version of the Birmingham Vasculitis Activity Index (BVAS 3.0), which is the most commonly used AAV activity score

Dual use research of concern

Policy information about dual use research of concern

Hazards

Could the accidental, deliberate or reckless misuse of agents or technologies generated in the work, or the application of information presented in the manuscript, pose a threat to:

No	Yes	
☒	☐	Public health
☒	☐	National security
☒	☐	Crops and/or livestock
☒	☐	Ecosystems
☒	☐	Any other significant area

Experiments of concern

Does the work involve any of these experiments of concern:

No	Yes	
☒	☐	Demonstrate how to render a vaccine ineffective
☒	☐	Confer resistance to therapeutically useful antibiotics or antiviral agents
☒	☐	Enhance the virulence of a pathogen or render a nonpathogen virulent
☒	☐	Increase transmissibility of a pathogen
☒	☐	Alter the host range of a pathogen
☒	☐	Enable evasion of diagnostic/detection modalities
☒	☐	Enable the weaponization of a biological agent or toxin
☒	☐	Any other potentially harmful combination of experiments and agents

Plants

Seed stocks	n/a
Novel plant genotypes	n/a
Authentication	n/a

ChIP-seq

Data deposition

☐ Confirm that both raw and final processed data have been deposited in a public database such as GEO.

☐ Confirm that you have deposited or provided access to graph files (e.g. BED files) for the called peaks.

Data access links<br>*May remain private before publication.*	n/a
Files in database submission	n/a
Genome browser session<br>(e.g. UCSC)	n/a

Methodology

Replicates	n/a
Sequencing depth	n/a
Antibodies	n/a
Peak calling parameters	n/a
Data quality	n/a
Software	n/a

Flow Cytometry

Plots

Confirm that:

- [x] The axis labels state the marker and fluorochrome used (e.g. CD4-FITC).
- [x] The axis scales are clearly visible. Include numbers along axes only for bottom left plot of group (a 'group' is an analysis of identical markers).
- [x] All plots are contour plots with outliers or pseudocolor plots.
- [x] A numerical value for number of cells or percentage (with statistics) is provided.

Methodology

Sample preparation	Human neutrophils were isolated from healthy donors or patients with AAV through negative selection by incubating whole blood with a cocktail of magnetically-labelled antibodies targeting non-target cells (#130-104-434, Miltenyi Biotec, Bergisch-Gladbach, Germany). The cells were removed by immunomagnetic depletion using a MACSxpress Separator (Miltenyi Biotec, Bergisch-Gladbach, Germany). The remaining cells were recovered and erythrocytes were eliminated using a MACSxpress Erythrocyte Depletion Kit (#130-098-196, Miltenyi Biotec). Neutrophil-enriched supernatant was collected, centrifuged and resuspended in RPMI complemented with 10% fetal calf serum (FCS), 2 mmol/L L-glutamine and penicillin-streptomycin. Isolated PNN were stained in PBS with viability marker (Fixable Viability Dye eFluor780, #13539140, eBiosciences, Santa Clara, CA, USA), anti-CD95 (CD95-Pacific Blue, #305619 BioLegend), or control isotype (IgG1k-Pacific Blue, #400151, BioLegend) in 5% BSA-PBS for 15 min (dark, room temperature) before washing and flow cytometry (Canto II, BD Biosciences) analysis.
Instrument	Canto II (BD Biosciences)
Software	Flowjo et Kaluza (Beckman coulter)
Cell population abundance	not mentioned
Gating strategy	Gating on living cells based on SSC/FSC parameters and singlet cells using FSC-H vs FSC-W parameters

- [x] Tick this box to confirm that a figure exemplifying the gating strategy is provided in the Supplementary Information.

Magnetic resonance imaging

Experimental design

Design type	n/a
Design specifications	n/a
Behavioral performance measures	n/a

Acquisition

Imaging type(s)	n/a
Field strength	n/a
Sequence & imaging parameters	n/a
Area of acquisition	n/a

Diffusion MRI [ ] Used [x] Not used

Preprocessing

Preprocessing software	n/a
Normalization	n/a
Normalization template	n/a
Noise and artifact removal	n/a
Volume censoring	n/a

Statistical modeling & inference

Model type and settings	n/a

Effect(s) tested | n/a

Specify type of analysis: ☐ Whole brain ☐ ROI-based ☐ Both

Statistic type for inference | n/a

(See Eklund et al. 2016)

Correction | n/a

Models & analysis

n/a	Involved in the study
☐	☐ Functional and/or effective connectivity
☐	☐ Graph analysis
☐	☐ Multivariate modeling or predictive analysis

Functional and/or effective connectivity | n/a

Graph analysis | n/a

Multivariate modeling and predictive analysis | n/a
