# Supplementary material for: Soluble CD95L triggers Caspase-10-driven reactive oxygen species production in neutrophils and aggravates anti-neutrophil cytoplasmic antibody-vasculitis
Source: Nat Commun. 2026 Jun 20;17:7759. doi: 10.1038/s41467-026-74452-8 (PMC13434393; doi:10.1038/s41467-026-74452-8)
Supplement: Supplementary file 4 [file 41467_2026_74452_MOESM4_ESM.pdf]

Description of Additional Supplementary Files

File name: Supplementary Data 1

Description: Shotgun proteomic analyses of peptides lost in DMSO-differentiated HL60 cells exposed to sCD95L or Ig-CD95L (100 ng/mL) in the presence or absence of z-VAD-fmk (50 microM).

File name: Supplementary Data 2

Description: Identification of CD95-driven caspase cleavage substrates in HL60 cells.
